# Quantifying research interests in 7,521 mammalian species with *h*-index: a case study

**DOI:** 10.1093/gigascience/giac074

**Published:** 2022-08-13

**Authors:** Jessica Tam, Malgorzata Lagisz, Will Cornwell, Shinichi Nakagawa

**Affiliations:** Evolution & Ecology Research Centre and School of Biological, Earth and Environmental Sciences, University of New South Wales, Sydney 2052, Australia; Evolution & Ecology Research Centre and School of Biological, Earth and Environmental Sciences, University of New South Wales, Sydney 2052, Australia; Evolution & Ecology Research Centre and School of Biological, Earth and Environmental Sciences, University of New South Wales, Sydney 2052, Australia; Evolution & Ecology Research Centre and School of Biological, Earth and Environmental Sciences, University of New South Wales, Sydney 2052, Australia

**Keywords:** bibliometrics, research bias, meta-research, scientific mapping, research on research, topic modeling

## Abstract

**Background:**

Taxonomic bias is a known issue within the field of biology, causing scientific knowledge to be unevenly distributed across species. However, a systematic quantification of the research interest that the scientific community has allocated to individual species remains a big data problem. Scalable approaches are needed to integrate biodiversity data sets and bibliometric methods across large numbers of species. The outputs of these analyses are important for identifying understudied species and directing future research to fill these gaps.

**Findings:**

In this study, we used the species *h*-index to quantity the research interest in 7,521 species of mammals. We tested factors potentially driving species *h*-index, by using a Bayesian phylogenetic generalized linear mixed model (GLMM). We found that a third of the mammals had a species *h*-index of zero, while a select few had inflated research interest. Further, mammals with higher species *h*-index had larger body masses; were found in temperate latitudes; had their humans uses documented, including domestication; and were in lower-risk International Union for Conservation of Nature Red List categories. These results surprisingly suggested that critically endangered mammals are understudied. A higher interest in domesticated species suggested that human use is a major driver and focus in mammalian scientific literature.

**Conclusions:**

Our study has demonstrated a scalable workflow and systematically identified understudied species of mammals, as well as identified the likely drivers of this taxonomic bias in the literature. This case study can become a benchmark for future research that asks similar biological and meta-research questions for other taxa.

## Introduction

Effective conservation of the earth's amazing biodiversity requires sound knowledge of species’ biology and ecology, with the addition of adequate communication from scientists [1]. However, such knowledge is often not only missing [2] but also biased. Some species receive disproportionally more research interest while others very little, reflected in scientific publications—known as taxonomic bias [3]. Although taxonomic bias in the scientific literature is prevalent [4, 5], there has been little effort to rectify the problem. Even worse, this problem seemed to have become more extreme in the last few decades [6,7]. To work toward reducing the gaps of knowledge in the literature, one first needs to understand what is causing such inequality in research interest among species.

Many potential drivers exist for taxonomic bias. For instance, there is a human preference to study and conserve iconic or “charismatic” taxa, which are usually large mammals such as the African bush elephant (*Loxodonta africana*) and black rhinoceros (*Diceros bicornis*) [8]. Indeed, large mammalian vertebrates are overrepresented in the conservation literature [9, 10]. Of relevance, the anthropomorphic stimuli hypothesis posits that humans are attracted to species that are more phylogenetically related to us [11]. Such human tendencies likely explain the inflated research effort toward vertebrate taxa [5]. This hypothesis is also related to the reason why we have much (bio)medical research, using rodent model systems such as rats (*Rattus norvegicus*) and mice (*Mus musculus*), because of our shared physiological traits [12]. Studying species closer to scientists’ proximity [5,13], where the animals live in accessible locations, and for economic reasons, such as agriculture and aquaculture research, can also exacerbate taxonomic bias in the literature. Consequently, these drivers have over time created strong unevenness in the taxonomic distribution of scientific knowledge.

Researchers have investigated such taxonomic bias in the academic literature, but these studies appeared to have 2 main shortcomings. First, because of the previous difficulties constructing scalable workflows, the coverage of these studies is often not comprehensive. While several studies have quantified species-level bias among plants [14], mammals [15–18], birds [19], fish [20], and amphibians [21], respectively, their sample sizes remain no more than a few hundred species, encompassing only small portions of species in a given taxonomic group. Until now, only 2 studies have evaluated species-level taxonomic bias for the thousands of species and across multiple clades [4,22]. However, these studies focused solely on species included on the International Union for Conservation of Nature (IUCN) Red List, therefore potentially failing to provide more comprehensive and holistic understanding of the drivers of taxonomic bias in research.

Second, there are currently no standardized methods to quantify taxonomic bias at the level of individual species. Publication count is one of the most commonly used proxies to gauge taxonomic bias [4, 5,7, 15, 18, 20–24]. However, while the total number of publications could capture the total research effort on a given species, it does not capture research interest per se (i.e., how much attention from the research community these publications received). A logical alternative would be to use citation count [25], as it captures the total research interest. Nonetheless, high-impact papers can easily inflate this number [26] and give a false impression that a species is receiving more interest than in reality. Hirsch's *h*-index [26] kills two birds with one stone by taking into account both the number of publications and number of citations. So far, there exist only a handful of studies that have adapted the “species” *h*-index for measuring and comparing research interests among different species [14,16, 17, 19, 27].

This study seeks to quantify the research interest in mammals, using the species *h*-index [14, 16, 17, 19]. We introduce a workflow demonstrating how to obtain species *h*-index for any species and how to ask relevant meta-science as well as biological questions on research interest. As a case study, we choose the class Mammalia, which consists of over 7,500, species, since they are one of the most well-studied taxonomic groups, with extensive data readily available. Then, we test how our surrogate for research interest, species *h*-index, could be related to the following 6 potential drivers: (i) body size, (ii) location of natural habitat, (iii) phylogenetic relatedness, (iv) human uses and domestication, (v) IUCN Red List status, and (vi) general interest (encompassing drivers i–v, quantified via Google Trends; see below). We outline our hypothesis and rationale for each potential driver in Table 1.

**Table 1:** Details of hypotheses

Potential driver	Hypothesis and rationale	Statistical surrogate	Data source
Size of species	We predict that higher body masses correlate with higher species *h*-index. Larger mammals (i.e., megafaunal species such as elephants and rhinoceroses) receive more research interest because they are generally considered more “charismatic” [8, 46].	Body mass (transformed with log_10_)	Wilman et al. [65]
Location of natural habitat	We predict that species found in temperate latitudes have higher species *h*-index. Mammals near the temperate zones attract more research interest as more researchers originate from these areas, such as North America, Europe, Australia, New Zealand, and southern Africa [5]. Thus, mammals whose natural habitat are within these regions are better studied.	Median latitude	GBIF [66]
Phylogenetic relatedness	We predict that there are phylogenetic signals present in the data set. Mammals that are more phylogenetically related receive similar species *h*-index because related species share similar traits that may influence the propensity of researchers to study members of a given clade [67]. Furthermore, species closer to humans will be overrepresented in species with high *h*-index values [11].	Branch lengths of phylogenetic tree	Upham et al. [35]
Human use & domestication	We predict that mammals with more human uses and domesticated mammals have higher species *h*-index. Some examples of human uses include transportation (e.g., horses and elephants), companionship (e.g., cats and dogs), food products (e.g., sheep and cattle), etc. Lab animals (e.g., rabbits and rodents) are likely to receive most research interest since the main purpose of keeping these animals is for scientific research [68, 69].	IUCN Red List human use categories & Wikipedia list of domesticated species	IUCN Red List [70] & Wikipedia [71]
Demography	We predict a U-shaped distribution of species *h*-index, where species in the “Least Concern” and “Critically Endangered” categories receive higher species *h*-index. Previous studies showed no correlations between the mammals’ IUCN Red List status and their research interest [16,17, 19].	IUCN Red List status	IUCN Red List [70]; cleaned with *rredlist* [72]
General interest	We predict that more general interest correlates with higher species *h*-index. Research and general interests are highly correlated since we tend to be more attracted to “charismatic” species, such as lions and elephants [46], and are more willing to donate for their conservation causes [73], resulting in more research interest.	Google Trends index	Google Trends [74]; extracted with *gtrendsR* [75]

We predicted that species *h*-index can be influenced by body sizes, location of natural habitat, phylogeny, human uses and domestication, demography, and general interest.

## Methods

### Data collection and processing

For much of data collection and cleaning as well as all statistical analyses (see below), we used the R language version 4.0.2 [28] in the RStudio environment version 1.3.1093 [29]. The source code of this article can be found on GitHub [30] and the data can be found on Zenodo [31,32].

We first collected a list of mammalian species from the Open Tree of Life (OTL) database [33] using the R package *rotl* version 3.0.12 [34] to create a complete mammalian species list. We removed subspecies from the list and only kept species with binomial names, resulting in 6,952 species. Next, we obtained lists characteristics of mammalian species represented as 7 statistical surrogates of the 6 potential drivers of research interest (Table 1): (i) body mass (*n* = 5,400; in grams, log_10_ transformed), (ii) median latitude of species range (*n* = 4,721; obtained from centroids of all occurrence records from GBIF (Global Biodiversity Information Facility)), (iii) phylogenetic trees with branch lengths (*n* = 5,911 [35]), (iv) IUCN Red List human use categories (*n* = 1,472; a binary categorical variable where a species was categorized into at least 1 of 19 human uses), (v) Wikipedia list of domesticated species (*n* = 159; a 3-level categorical variable: domesticated, partially domesticated, and wild), (vi) IUCN Red List status (*n* = 5,584; an ordinary variable with 6 levels: “Least Concern,” “Near Threatened,” “Vulnerable,” “Endangered,” “Critically Endangered,” and “Extinct in the Wild” excluding “Extinct” and “Data Deficient”; there was some discrepancy with the IUCN Red List statuses when filling in missing data in this category; we suspect this is an issue caused by a mismatch between the data on the IUCN Red List website and their API (Application Programming Interface - an API enables the communication between two applications, in this instance, extracting data from IUCN Red List's database using a local computer) through the *rredlist* R package, and we suggest using data from the website as the package might not be regularly maintained), and (vii) Google Trends index (*n* = 7,521; see Supplementary Fig. S1 for a summary of the data completeness and data-processing details and see the Supplementary information). Synonym matching was performed automatically with *rotl::tnrs_match_names()*, before combining the categories and the list from OTL to form 1 data set. Duplicated names were removed using the functions *unique()* and *duplicate()*. A total of 7,521 unique species remained on the final species list. We obtained the Google Trends index after finalizing the list of species names.

Notably, we added higher taxonomic clades to condense the 30 orders to 5 major clades according to molecular tree reconstructions [35,36]. These 5 high-lever taxa are (i) Afrotheria, representing an African lineage, including sea cows and elephants; (ii) Xenarthra, representing an American lineage that includes sloths and armadillos; (iii) Euarchontoglires, representing widely distributed species such as rodents and primates; (iv) Laurasiatheria, representing species such as whales, carnivores, and bats; and finally, (v) Marsupials and Monotremes, representing the noneutherian mammals. We used these higher taxonomic groupings in visualizations of the results.

#### Data sources and species h-index

We extracted the bibliometric records from Scopus (data collection on 28 April 2021) and calculated the *h*-index of individual mammal species with the R package *specieshindex* [37]. The package connects to the Scopus, Web of Science, and Bielefeld Academic Search Engine (BASE) literature databases. Using either binomial or genus names, the package can count the number of relevant bibliometric records for each species or genus on each database and extract them for local processing and analysis. Bibliometric information that can be extracted include citation count, publication date, authors, and more. *specieshindex* can then calculate the species *h*-index of individual species applying Hirsch's *h*-index [26]. The *h-*index is defined as the largest number of publications (*n*) cited a minimum of the same number (*n*) of times (Supplementary Fig. S2). The *h*-index in this scenario quantifies the research interest each individual species has received. The package has also implemented the calculation of other indices, such as the *m*-index and *h5* index, and plotting functionality.

We used binomial names in Scopus database searches because of the ambiguity and lack of common names for uncommon species. We tackled the issue of species name synonyms by using the Boolean term “OR” between each synonymous binomial name (collected from OTL) in the search string. Articles containing binomial names of mammals in their title, abstract, or keywords were extracted. Since the distribution of *h*-index was right-skewed with more species having a lower species *h*-index, we applied the formula $lo{g_{10}}( {h + 1} )$ for visualization purposes, but we used the original count data for modeling (see below).

#### Imputing missing data

The coverage of data is lower for some predictors (Supplementary Fig. S1) as a result of synonym matching and cleaning. Since some data were missing for body mass, latitude, and IUCN Red List status (Supplementary Fig. S1), we imputed missing values for 5,497 species that were included in the model, to match the shorter length of the phylogenetic tree. We used the multiple imputation approach implemented in the R package *mice* [38]. Multiple imputation creates multiple sets of imputed values before aggregating them to create a single set of data [39]. This is preferred over deletions of data records with missing values, as the latter can result in lowered statistical power and biases in the parameter estimates [40]. We used binomial name, *h*-index, human use, domestication, and Google Trends index to impute 3 variables with missing values (body mass, latitude, and IUCN Red List status), creating 10 complete data sets for statistical analyses.

### Statistical analysis and phylogenetic “heritability”

We ran 2 Bayesian phylogenetically controlled Poisson mixed models with the log link function and the additive dispersion term [41], implemented in the R package *MCMCglmm* version 2.33 [42], and ran using the computation cluster Katana at UNSW Sydney [43]. The first model followed the predictions stated in the hypotheses (Table 1) and used the data set with the sample size of 5,497 species and 50 identical phylogenetic trees with branch lengths chosen randomly from Upham et al. [35]. Fifty trees were selected since it is the minimum number of trees needed to account for uncertainties in phylogenetic data [44]. The second model was the same as the first one but with only 5,343 species after removing domesticated and semi-domesticated species (i.e., 1 less predictor or fixed effect than the first 2 models; see formulae below). We added this model because (semi-)domesticated species are likely to have inflated species *h*-index values that may not be comparable to those of wild species.

We ran 130,000 iterations for the chain with 30,000 burn-ins, drawing 1,000 samples from the imputed data in each iteration, and using a noninformative prior for both fixed and random effects. To obtain more accurate precision of model estimates, we repeated the same model for the 10 imputed data sets and 50 phylogenetic trees, resulting in a total of 500 model runs for each model, respectively. The last 100 of the total 1,000 samples of each model were extracted for the calculation of the model results.

In the first model, we used the following predictor variables: body mass value on log_10_ scale (continuous), the absolute value of median latitude (continuous; converted to absolute value for linear distribution), human use (binomial), domestication (ordinal), IUCN Red List status (ordinal), and Google Trends index on (log_10_ + 1) scale (binomial) to model the outcome variable species *h*-index (count), as in the following formula:

\begin{equation*} h\,\,\sim\,\,{log_{10}}\left( {Body\ mass} \right) + \left| {Latitude} \right| + \ Human\ use + Domestication \nonumber\\ + \ IUCN\ Red\ List\ status + {\left( {IUCN\ Red\ List\ status} \right)^2} \nonumber\\ + lo{g_{10}}\left( {Google\ Trends + 1} \right)\end{equation*}

The second model is in the following formula (without domestication):

\begin{equation*}h\,\,\sim\,\,{log_{10}}\left( {Body\ mass} \right) + \left| {Latitude} \right| + \ Human\ use \nonumber\\ + \ IUCN\ Red\ List\ status + {\left( {IUCN\ Red\ List\ status} \right)^2}\nonumber\\ + lo{g_{10}}\left( {Google\ Trends + 1} \right)\end{equation*}

During our preliminary analysis, we checked for variance inflation factor (VIF) to make sure that the regressors were not correlated to each other. The VIF values ranged between 1.0 and 1.7 (Supplementary Table S3). Low VIF values meant that the predictor variables are not colinear and will not lead to inflated correlations.

We estimated phylogenetic heritability (*H*^2^; [41]) to check for phylogenetic correlations among species, which is equivalent to Pagel's *lambda* (λ). Values of *H*^2^ fall between 0 and 1. The output of the Bayesian model provided the values needed for *H*^2^ calculation using the following formula, from Nakagawa et al. [45]:

\begin{equation*} {H^2}\ = \ \frac{{var\left( {species} \right)}}{{var\left( {species} \right) + \ var\left( {overdispersion} \right) + \ ln\left( {1 + \frac{1}{{mean\left( h \right)}}} \right)}}
\end{equation*}

where *var*(*species*) and *var*(*overdispersion*) are the variance components for phylogenetic effects and the additive overdispersion term, which is equivalent to the residual term in a normal regression, and *mean*(*h*) represents the average *h*-index values.

## Results

### General trends of species’ *h*-index across taxa

**Figure 1:**
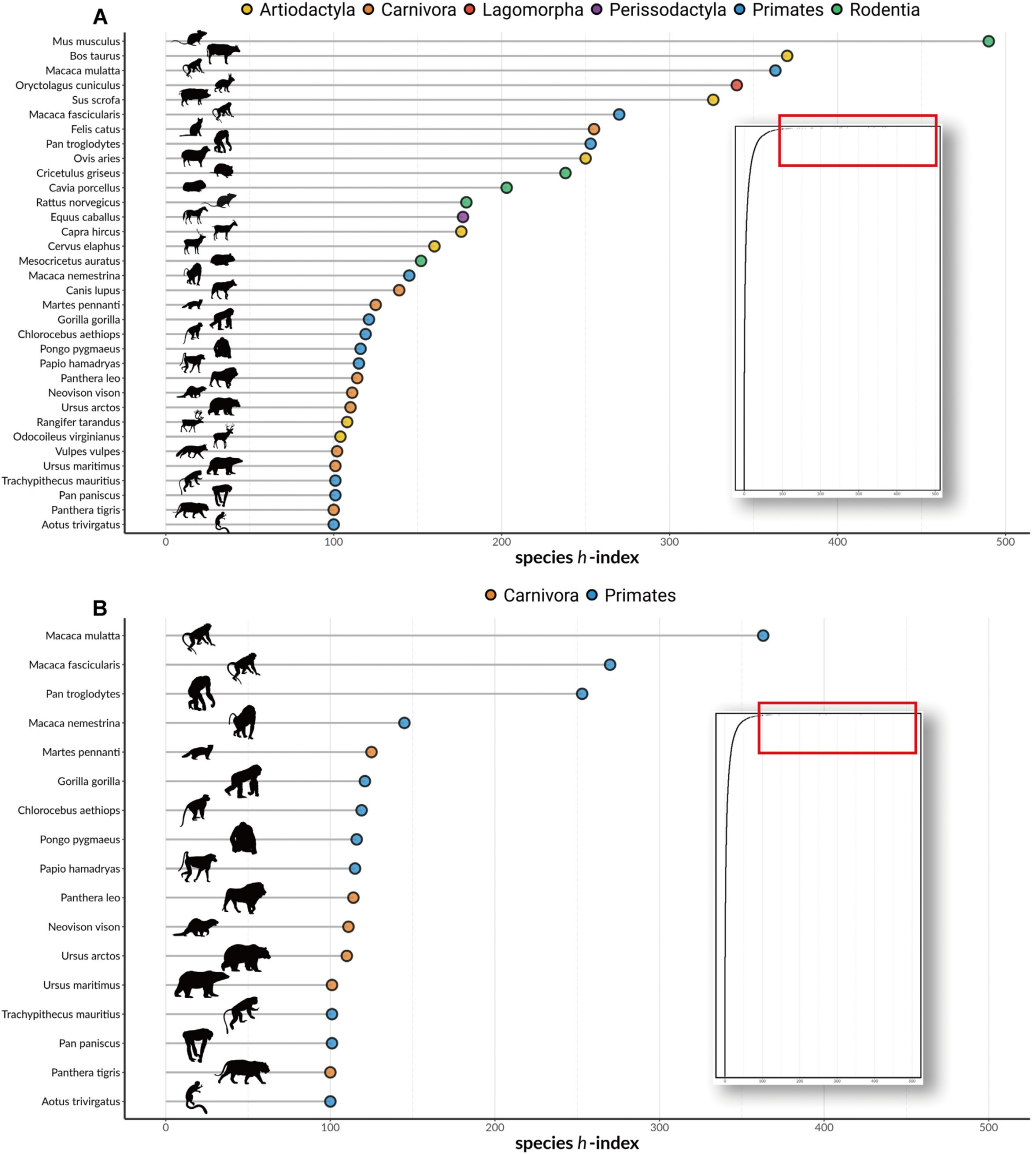
: Species *h*-index of mammals. Panel (A) shows 34 mammals with *h* = 100 or more, representing 6 different orders marked by dots of different colors. Figure in the inset shows the distribution of species *h*-index of all mammals, with the species scoring above *h* = 100 or more marked by the red box. Panel (B) shows the mammals with *h* = 100 or more but removes domesticated species, with 17 species left.

We calculated the species *h*-index for 7,521 species of mammals in total. A species *h*-index of 0 was common in mammals with 32.26% (*n* = 2,426; Supplementary Fig. S4) failing to have even 1 paper cited 1 time (Fig. 1). On the other hand, mammals with a species *h*-index of 100 and higher only included 34 species from across 6 orders (Fig. 1A). The median and mean of the *h*-index for all the species were *h*_median_ = 2 and *h*_mean_ = 7.08, respectively. After removing domesticated (and semi-domesticated) species from the data set (remaining *n* = 7,360), mammals with a *h*-index of 100 and higher only included 17 species from Carnivora and Primates (7 and 10 species, respectively; Fig. 1B). The median and mean of the species *h*-index without the domesticated mammals are *h*_median_ = 2 and *h*_mean_ = 6.16, respectively.

**Figure 2:**
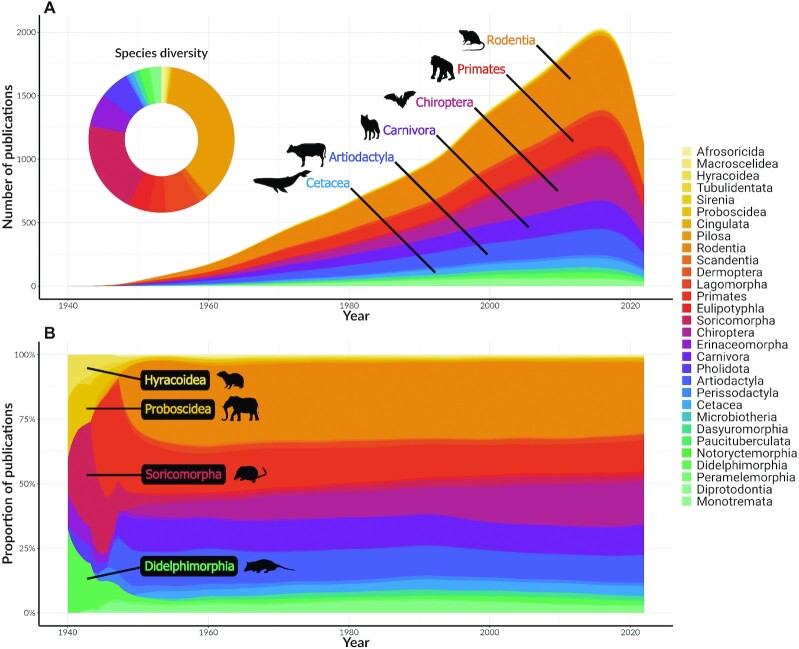
: The changes in mammalian literature from 1940 to 28 April 2021. (A) The number of publications per year for 30 mammalian orders and the proportion of species per order from the collated mammalian data set represented by the doughnut chart, and (B) change in the frequency of publications on 30 mammalian orders present in the data set. Total number of mammalian species analyzed is 7,521.

**Figure 3:**
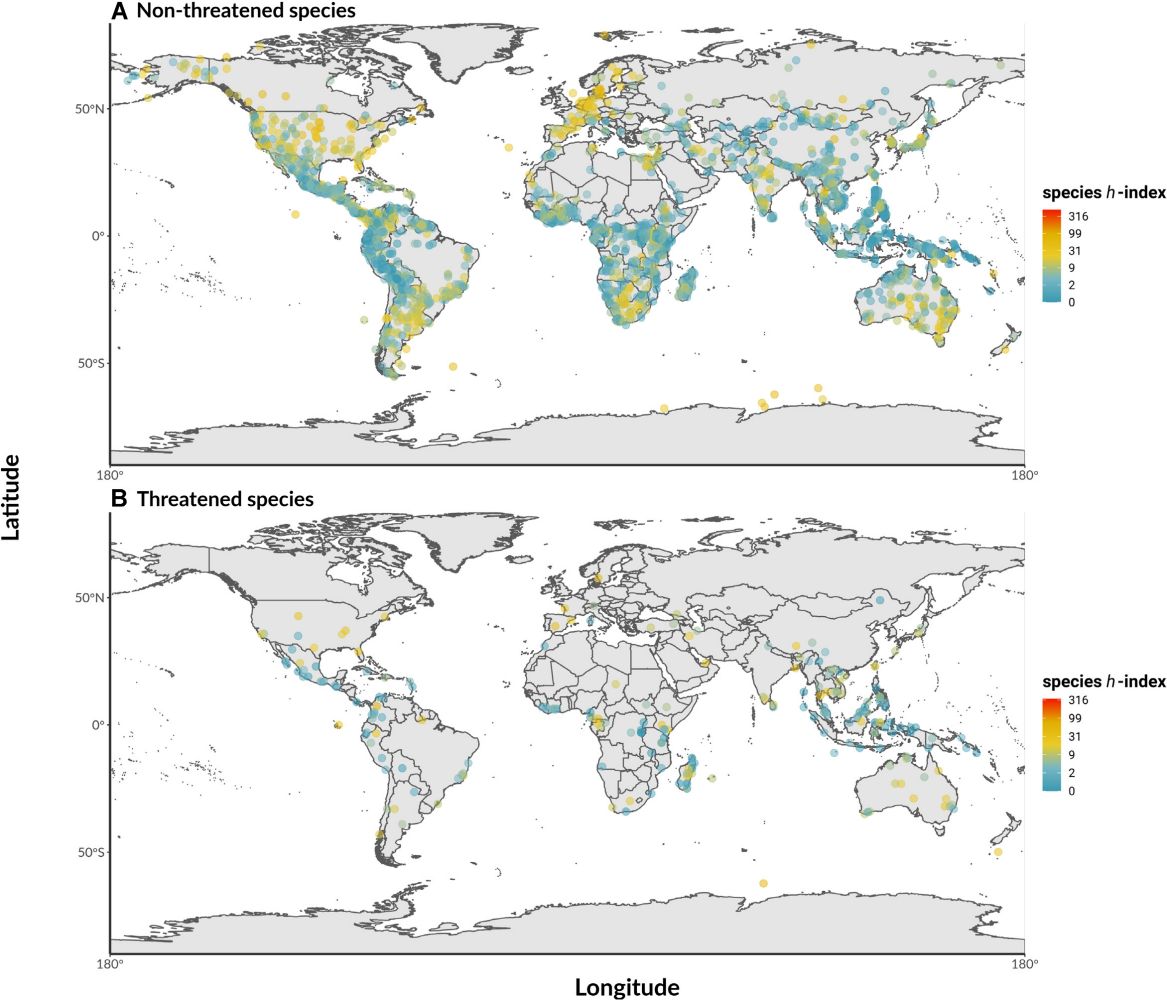
: Centroids of global distributions of 4,744 mammalian species. (A) The distribution of nonthreatened species listed as “Least Concern”. (B) The distribution of threatened species listed as “Vulnerable”, “Near Threatened”, “Endangered”, “Critically Endangered”, and “Extinct in the Wild”. The species’ corresponding *h*-index values are illustrated by dot color.

**Figure 4:**
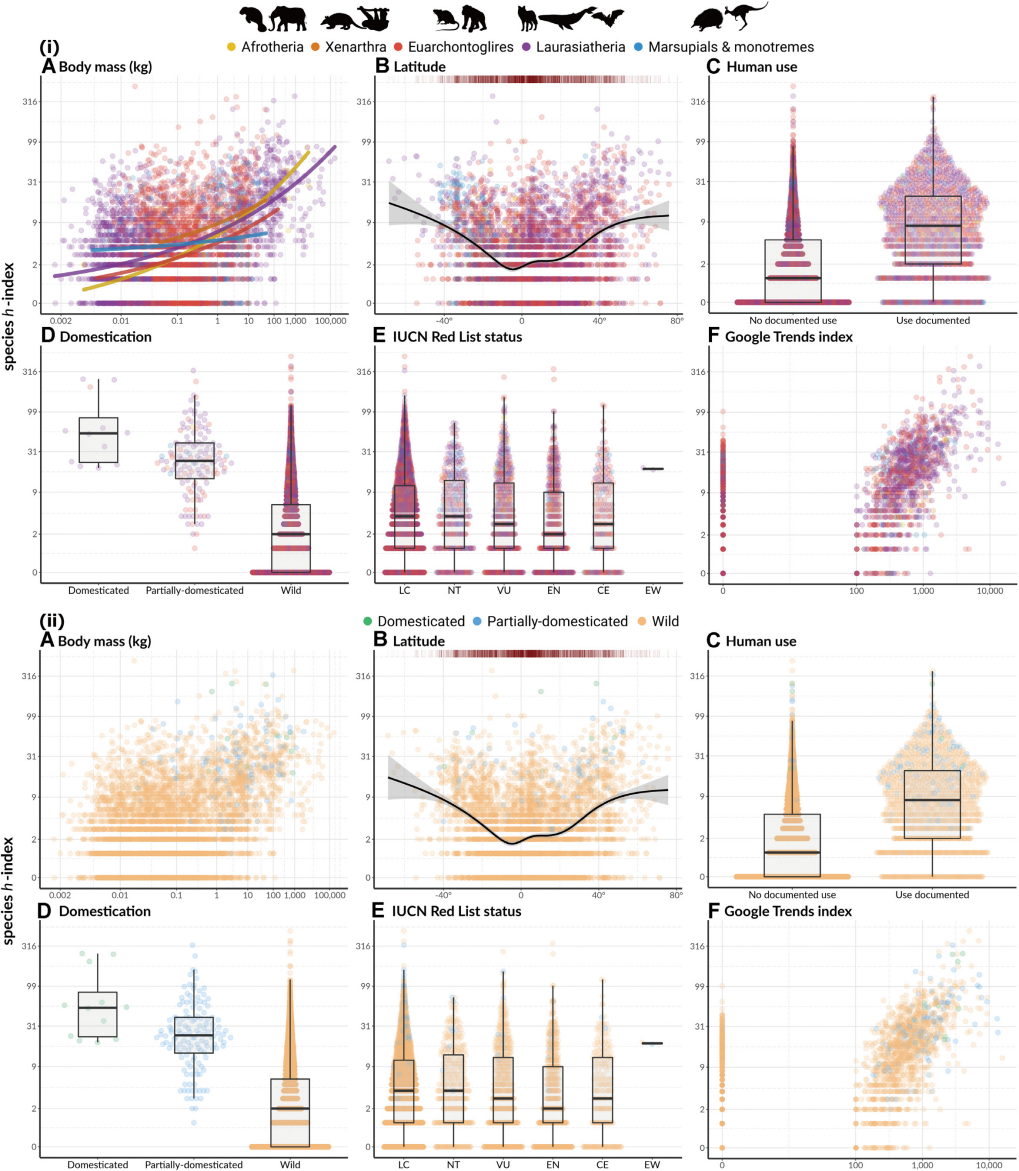
: Relationship between predictor variables and species *h*-index values. (i) (A) Species average body mass (*n* = 5,158 species, fitted curves represent 50% quantile for each clade). (B) Median latitude of species geographical distribution (*n* = 4,435 species, fitted curve from generalized additive model [GAM] with shaded gray area representing 95% confidence interval; density bar on top of the plot illustrates the number of species at each latitude). (C) Human use categories (*n* = 7,521, No documented use = 6,124 and Use documented = 1,397). (D) Domestication status (*n* = 7,521 species, Domesticated = 12, Partially domesticated = 136, and Wild = 7,373). (E) IUCN Red List status (*n* = 5,584 species, Least Concern = 3,152, Near Threatened = 340, Vulnerable = 530, Endangered = 512, Critically Endangered = 208, and Extinct in the Wild = 2). (F) Google Trends index summed for each species (*n* = 7,521 species, Google Trends index > 0 = 1,323, and Google Trends index = 0 = 6,124 species). Box plots in (C), (D), and (E) show the median, 25th and 75th percentiles, and lower and upper extremes. (ii) Showing the same data as (i), except each species is colored according to their domestication status.

There were also pronounced shifts in research interest through time. Publications in the early 1940s were largely on the orders Hyracoidea (hyraxes), Proboscidea (elephants), Soricomorpha (dissolved paraphyletic taxa of shrews—combined with Erinaceidae to form Eulipotyphla), and Didelphimorphia (opossums) (Fig. 2B). Upon skimming the titles of some articles (around 10 titles), we noted that early publications in these species appeared to be mostly comparative anatomy studies. In the 1950s, the mammalian literature took on its modern structure, with research focused largely on 6 orders (Fig. 2A)—rodents (Rodentia, 1950–2021 mean = 30.94% of the yearly article count); primates (1950–2021 mean = 13.98%); bats (Chiroptera, 1950–2021 mean = 11.16%); carnivores (Carnivora, 1950–2021 mean = 11.61%); pigs, sheep, cattle, and other even-toed ungulates (Artiodactyla, 1950–2021 mean = 11.83%); and whales and dolphins (Cetacea, 1950–2021 mean = 3.15%). Higher species *h*-index was generally associated with larger body sizes (Fig. 4A), intermediate latitudes (Fig. 3, Fig. 4B), more human uses (Fig. 4C) and domestication (Fig. 4D), lower extinction risk (Fig. 4E), and higher general interest (Fig. 4F).

### Statistical predictors of species’ *h*-index and phylogenetic signal

We included 5,497 species of mammals in the first (Table 2) and 5,343 species (after excluding domesticated animals) in the second (Table 3) Bayesian generalized linear mixed model (BGLMM). In both models, body size positively and significantly predicted species *h*-index (Tables 2 & 3; Fig. 4A). While mammalian diversity was highest in the tropics, species found here had significantly lower species *h*-indices compared to those in the temperate regions and near the poles, which was again supported in the models (Tables 2 & 3; Fig. 3; Fig. 4B). Although most mammals had a Google Trends index of 0, species *h*-index significantly increased with the Google Trends index in all models (Tables 2 & 3; Fig. 4F). There was a U-shaped distribution across the IUCN Red List statuses (Fig. 4E) with a statistically significant quadratic effect in both models (i.e., with and without domesticated animals; Tables 2 & 3; see also Supplementary Fig. S5 for IUCN Red List statuses not included in the model). These models also showed a statistically significant linear decline of species *h*-index with increasing extinction risk (IUCN Red List status). Further, species *h*-index significantly increased with human use in both models (Tables 2 & 3; Fig. 4C; see Supplementary Fig. S6 for all human use categories). The first 2 models showed that domestication status was a significant positive predictor of species *h*-index (Tables 2 & 3; Fig. 4D). Finally, phylogenetic signal was present in species *h*-index in both models (Tables 2 & 3; see Supplementary Fig. S7 for the phylogenetic tree).

**Table 2:** Summary of statistical results from the Bayesian Generalized Linear Mixed Model (BGLMM)

Estimate	Mean	95% Credible interval (CI)
*Fixed effects*		
Intercept	1.310	−0.119, 2.743
log_10_(body mass)	0.097	0.037, 0.155
Latitude (absolute value)	0.022	0.019, 0.025
IUCN Red List status (first-degree polynomial)	−16.397	−19.323, −13.569
IUCN Red List status (second-degree polynomial)	2.759	0.323, 5.206
Human use	0.268	0.166, 0.371
Domestication status	−0.376	−0.547, −0.206
log_10_(Google Trends)	0.490	0.457, 0.522
*Random effects*		
Phylogeny	1.601	1.080, 2.230
Nonphylogeny	0.807	0.745, 0.871
Phylogenetic heritability (*H*^2^)	0.637 (*0.642)	0.000, 0.660 (*0.516, 0.660)

*Phylogenetic signal after removing 1,124 species (20.4%) from the tree that showed no signals.

The distributions here follow the distributions stated in the hypothesis.

**Table 3:** Summary of statistical results from the Bayesian Generalized Linear Mixed Model (BGLMM)

Estimate	Mean	95% Credible interval (CI)
*Fixed effects*		
Intercept	0.149	−1.163, 1.451
log_10_(body mass)	0.105	0.043, 0.165
Latitude (absolute value)	0.022	0.019, 0.025
IUCN Red List status (first-degree polynomial)	−16.539	−19.453, −13.692
IUCN Red List status (second-degree polynomial)	3.021	0.581, 5.469
Human use	0.277	0.171, 0.382
log_10_(Google Trends)	0.501	0.469, 0.534
*Random effects*		
Phylogeny	1.573	1.055, 2.209
Nonphylogeny	0.818	0.755, 0.882
Phylogenetic heritability (*H*^2^)	0.626 (*0.633)	0.000, 0.652 (*0.510, 0.653)

*Phylogenetic signal after removing 1,124 species (21.0%) from the tree that showed no signals.

The distributions here follow the distributions stated in the hypothesis, except we removed domestication status and domesticated and partially domesticated species.

## Discussion

Scientific research is not spread evenly across mammal species: we found strong bias in “research interest” in the literature, quantified by species *h*-index. A small group of species (*n* = 34 with all species and *n* = 17 without domestication species) had a species *h*-index above 100, while one-third of the species (*n* = 2,426 with or without domestication species) received no scientific interest at all (*h* = 0) (Fig. 1). The modern mammalian literature was dominated by the orders Rodentia, Primates, Carnivora, Artiodactyla, Chiroptera, and Cetacea (Fig. 2), which resulted in a high value of phylogenetic heritability in the model (*H*^2^ = 64%; Table 2). Overall, our analyses confirmed our predictions (Table 1). The bias toward a few orders also appeared in species with high species *h*-indices (Fig. 1) and these commonly found in the high latitudes (Fig. 3). Mammals with high species *h*-indices were more likely to be large, be less endangered, and have their utility documented (Fig. 4). These “research superstars” include farmed animals, pets, and small laboratory mammals, as expected.

### Low research interest in endangered small mammals

The relationship between IUCN Red List status and species *h*-index (Fig. 4D) resembled a U-shaped distribution, and this trend was statistically significant in both models with and without domesticated animals (Table 3). Also, we found a significant decline (a significant linear effect) in research interest (species *h*-index) with conservation status (i.e., for more endangered mammals). Collectively, these quadratic and linear effects indicate that some endangered species may enjoy higher species *h*-indices, such as the lion (*Panthera leo*) and the orangutan (*Pongo pygmaeus*) (Fig. 1).

We also found that species *h*-index is positively related to increasing body mass (Fig. 4A). These findings could jointly indicate that larger mammals that are less endangered could be attracting more research attention than smaller mammals that are severely endangered. Since taxa with larger mammals, such as the big cats and African megafauna, are typically considered more charismatic [8, 46], larger mammals may receive more research interest than smaller mammals, regardless of whether or not they are threatened (Fig. 3B). We found that taxa with smaller mammals in the IUCN Red List categories “Endangered” and “Critically Endangered” were likely to have slightly lower species *h*-indices. This indicates a lack of research focus on smaller species, especially those endangered, possibly because they are rarer in the wild and comparatively harder to research.

### High research interest with domestication and phylogenetic relatedness

Domesticated species were among the top ranks of mammals with the highest species *h*-indices (Fig. 1A, Fig. 4D). Mammals with human uses documented also had higher species *h*-indices than species with no documented human uses (Fig. 4C). However, some species lack documentation on human uses because the data on human uses are patchy and not reliable for locally used species. The strong focus on pets and livestock animals can be explained by their global proximity to humans as well as our needs and preferences. Among all mammals on earth, wild mammals make up only 4% of the total mammalian biomass, while humans and livestock combine to form the other 96% [50], and this corresponds with their widespread occurrence due to the globalization of a small number of animal husbandry systems [51]. Our need to make our animal use more efficient has clearly driven high volumes of research on these animals.

For example, the literature on cattle or sheep can have contributions and interested readers from all over the world. The broad readership creates academic rewards for researchers and thus positive feedback toward an ever-expanding literature on these animals. In contrast, the research on the grizzled tree-kangaroo, a vulnerable wild species, can only be done on New Guinea and surrounding islands, severely limiting both the pool of potential researchers and potential readers of that research. Thus, not only is it financially and logistically difficult to research grizzled tree-kangaroos, but the readership and academic rewards for doing research in species without any direct human uses are very limited.

We also found phylogenetic signals in species *h*-indices (Tables 2 & 3), meaning some taxonomic groups usually had higher *h*-indices than others (Fig. 1). Many livestock animals are phylogenetically related, such as the pig (*Sus scrofa*), the sheep (*Ovis aries*), and the cow (*Bos taurus*) (Fig. 1A), all of which belong to the order Artiodactyla. Furthermore, several primates had relatively high species *h*-indices compared to those from other taxa. Indeed, when we removed the domesticated species, around 65% of the species with *h* = 100 or more were primates (Fig. 1B). This finding strongly supports the anthropomorphic stimuli hypothesis [11], where humans tend to be more attracted to species that are phylogenetically similar to us.

### Geographical bias toward species in developed countries

We found that mammals with higher species *h*-indices were congregated in clusters centered at the temperate latitudes (Fig. 3, Fig. 4B). Some of these locations—in the United States, Europe, and Australia—are regions with high gross domestic product values, GDP [52], characteristic for developed countries. Not only are scientists in developed countries able to carry out more research activities with better funding, but they have better access to the infrastructure, such as laboratories, transport, and equipment. Higher education is also better implemented in these regions, which is largely lagging in developing countries [53,54]. Developing countries often require even more research funding to compensate for the scarcity of resources [55]. Since developed countries dominate global publication output [56], the geographical biases revealed in our analyses therefore reflect the research interests of scientists in wealthier countries.

Academic preferences toward certain mammal species also suggest that convenience is often prioritized. This trend is evident in Fig. 3, where species near the tropics had much lower species *h*-indices than those in temperate zones, regardless of their extinction risk. Such preference toward species in the temperate zone is not unique to Mammalia. Scientific literature on species across all taxa, both vertebrates and invertebrates, is biased toward the temperate environment [57]. This is alarming given that 55% of species in the tropics are at risk of local extinctions from climate change, which is higher than that of temperate species, at 39% [58]. At the same time, tropical regions are biodiversity hotspots because of their high species richness [59]. However, considering that funding in science is often limited, projects that yield the best results with the lowest cost may receive more resources and support.

### Potential limitations and future perspectives

This study has 4 major limitations. First, the data sources included varying lists of mammals with available information, resulting in missing values in some of our predictors (body mass, latitude, and IUCN Red List status) (Supplementary Fig. S1). Although this issue was mitigated by imputing values, the results of our study would be more reliable if complete data were available. Further, some species may have been dropped from the analyses as their binomial names were spelled differently from the current consensus name. Although we attempted to incorporate synonyms and remove species that went extinct during the prehistorical and historical times, some synonyms with different spellings and extinct species might still be present in the data set. This can potentially explain why the sample size of this study is 7,521 species of mammals, much higher than Burgin et al.’s [60] resolved list with only 6,495 species. The issue of unresolved taxonomy is likely going to affect similar studies that attempt to gather high volumes of data for multiple species from other taxa [61].

Second, we used the *h*-index [26] as a measurement of research interest since it takes into account both number of publications and numbers of citations. However, there are other similar indices that can be used to quantify research output and influence, including the *h5* index, *m*-index, and *i10* index. The *h5* index is the *h*-index of publications that were published in the past 5 years [62]. The *m*-index is the *h*-index divided by the number of years since the first publication [26], which directly scales for time (Supplementary Fig. S8). Indirectly, the *h*-index can also indicate the time dimension, assuming that more time associates with more publications and more citations. The *i10* index is the total number of articles with 10 or more citations; it is currently used by Google Scholar [63]. Future studies can compare these indices and investigate how they differ with *specieshindex* R package, which can calculate these other indices.

Third, we used species *h*-index here to characterize the distribution of research interest across mammalian species. More research interest does not inform us on the kinds of research that has been done for a given species. Text mining could be used on full-text publications to single out studies on a given topic (e.g., conservation, behavior, ecology, or biomedical use) in future studies, although such an endeavor would require access to full texts.

Finally, although a proxy for general interest in species, presence in Google searches, was a strong and statistically significant predictor of species *h*-index (Fig. 4C, Supplementary Table 2), members of the public, in general, are unlikely to use binomial names of species, which we used in this study. We decided against the use of common names for our analyses as many species have multiple common names and many common species names are often used the name of products or companies, such as “Tiger Corporation”, which is a Japanese manufacturer of household products, and our searches would result in very messy data. Therefore, we require a better proxy for quantifying public interest in different species.

## Conclusion

This study has quantified species *h*-index for all available mammalian species as a case study and asked meta-scientific and biological questions. We have elucidated the current patchiness and biases in the mammalian research landscape using potential drivers of such biases that have been hypothesized before, but perhaps at the largest and finest scale than previously done. More importantly, we have demonstrated potential of addressing meta-research and biological questions by combining available online data sets and species *h*-indices calculated from a bibliometric database. Therefore, future studies can ask a rich set of similar and extended questions to quantify the research landscape of any taxa.

## Data Availability

The source code of this article can be found on GitHub [30] and the data can be found on Zenodo [31, 32]. Additional information is available at the end of the article as supplementary material. An archival copy of the data sets and GitHub Repository is available via the GigaScience database, GigaDB [64].

## Abbreviations

IUCN: International Union for Conservation of Nature

OTL: Open Tree of Life

GBIF: Global Biodiversity Information Facility

API: Application Programming Interface

MCMCGLMM / GLMM: Markov chain Monte Carlo Generalised Linear Mixed Model

VIF: Variance Inflation Factor

## Supplementary Material

giac074_GIGA-D-21-00396_Original_Submission

giac074_GIGA-D-21-00396_Revision_1

giac074_GIGA-D-21-00396_Revision_2

giac074_Response_to_Reviewer_Comments_Original_Submission

giac074_Response_to_Reviewer_Comments_Revision_1

giac074_Response_to_Reviewer_Comments_Revision_2

giac074_Reviewer_1_Report_Original_Submission Emilio Berti -- 12/17/2021 Reviewed

giac074_Reviewer_2_Report_Original_Submission Louise McRae -- 1/14/2022 Reviewed

giac074_Reviewer_2_Report_Revision_1 Louise McRae -- 5/18/2022 Reviewed

giac074_Supplemental_Files

## Additional Files

**Supplementary Fig. S1**. Summary of the data collected and treatments assigned for analysis. Completeness of data is the proportion of available data out of *n* = 7,521.

**Supplementary Fig. S2**. Graphical explanation of the *h*-index. The *h*-index is obtained by ranking the citations of each paper in descending order and then finding the number of papers (*h*) with *h* number of citations. The sixth most cited publication has been cited at least 6 times, as indicated by the blue dotted lines. Hence, the *h*-index is 6.

**Supplementary Fig. S4**. Frequency of species with each species *h*-index. n_all_ = 7,521, n*_h_*  _= 0_ = 2,426.

**Supplementary Fig. S5**. Distribution of individual species *h*-index for all IUCN Red List categories. Box plots show the median, 25th and 75th percentiles, and lower and upper extremes.

**Supplementary Fig. S6**. Distribution of individual species *h*-index for all human use categories. Box plots show the median, 25th and 75th percentiles, and lower and upper extremes.

**Supplementary Fig. S7**. Phylogenetic tree of 5,497 mammalian species included in the analyses. Five major clades are shown in different colors. Silhouettes representing the top 9 mammals with the highest species *h*-index.

**Supplementary Fig. S8**. Species *m*-index of mammals. The plot shows the mammals with *m* > 1, representing 9 different orders marked by dots of different colors. Figure in the inset shows the distribution of species *m*-index of all mammals, with the species scoring above *m* > 1 or more marked by the red box.

**Supplementary Table S3**. Variance inflation factors of the moderators from the quasi-Poisson generalized linear model (GLM).
