# Supplementary material for: Quantifying research interests in 7,521 mammalian species with h-index: a case study
Source: Gigascience. 2022 Aug 13;11:giac074. doi: 10.1093/gigascience/giac074 (PMC9375528; doi:10.1093/gigascience/giac074)
Supplement: giac074_GIGA-D-21-00396_Revision_1 [file giac074_giga-d-21-00396_revision_1.pdf]

GigaScience

Quantifying research interests in 7,521 mammalian species with h-index: a case study

--Manuscript Draft--

**Manuscript Number:**	GIGA-D-21-00396R1
**Full Title:**	Quantifying research interests in 7,521 mammalian species with h-index: a case study
**Article Type:**	Research
**Funding Information:**	
**Abstract:**	Background<br><br>Taxonomic bias is a known issue within the field of biology, causing scientific knowledge to be unevenly distributed across species. However, a systematic quantification of the research interest that the scientific community has allocated to individual species remains a big data problem. Scalable approaches are needed to integrate biodiversity datasets and bibliometric methods across large numbers of species. The outputs of these analyses are important for identifying understudied species and directing future research to fill these gaps.Findings<br><br>In this study, we used the species h-index to quantity the research interest in 7,521 species of mammals. We tested factors potentially driving species h-index, by using a Bayesian phylogenetic generalised linear mixed model (GLMM). We found that a third of the mammals had a species h-index of zero, while a select few had inflated research interest. Further, mammals with higher species h-index had larger body masses, were found in temperate latitudes, had more humans uses, including domestication, and were in lower risk IUCN Red List categories. These results surprisingly suggested that critically endangered mammals are understudied. A higher interest in domesticated species suggested that human use rather than conservation drives mammalian scientific literature.Conclusion<br><br>Our study has demonstrated a scalable workflow and systematically identified understudied species of mammals, as well as identified the likely drivers of this taxonomic bias in the literature. This case study can become a benchmark for future research that asks similar biological and meta-research questions for other taxa.
**Corresponding Author:**	Jessica Tam<br>University of New South Wales - Kensington Campus: University of New South Wales<br>Kensington, New South Wales AUSTRALIA
**Corresponding Author Secondary Information:**	
**Corresponding Author's Institution:**	University of New South Wales - Kensington Campus: University of New South Wales
**Corresponding Author's Secondary Institution:**	
**First Author:**	Jessica Tam
**First Author Secondary Information:**	
**Order of Authors:**	Jessica Tam
	Malgorzata Lagisz
	Will Cornwell
	Shinichi Nakagawa
**Order of Authors Secondary Information:**	
**Response to Reviewers:**	We have compiled the comments into a PDF documented. It has been uploaded as a 'Personal cover', with the description of 'Addressing reviewers' comments', and document name 'Manuscript comments'.
**Additional Information:**	

Powered by Editorial Manager® and ProduXion Manager® from Aries Systems Corporation

Question	Response
Are you submitting this manuscript to a special series or article collection?	No
**Experimental design and statistics**<br><br>Full details of the experimental design and statistical methods used should be given in the Methods section, as detailed in our Minimum Standards Reporting Checklist. Information essential to interpreting the data presented should be made available in the figure legends.<br><br>Have you included all the information requested in your manuscript?	Yes
**Resources**<br><br>A description of all resources used, including antibodies, cell lines, animals and software tools, with enough information to allow them to be uniquely identified, should be included in the Methods section. Authors are strongly encouraged to cite Research Resource Identifiers (RRIDs) for antibodies, model organisms and tools, where possible.<br><br>Have you included the information requested as detailed in our Minimum Standards Reporting Checklist?	Yes
**Availability of data and materials**<br><br>All datasets and code on which the conclusions of the paper rely must be either included in your submission or deposited in publicly available repositories (where available and ethically appropriate), referencing such data using a unique identifier in the references and in the "Availability of Data and Materials" section of your manuscript.	Yes

Powered by Editorial Manager® and ProduXion Manager® from Aries Systems Corporation

Have you have met the above requirement as detailed in our Minimum Standards Reporting Checklist?	

Manuscript

Click here to access/download;Manuscript;Biases_in_mammalian_research_2

Quantifying research interests in 7,521 mammalian species with *h*-index: a case study

Jessica Tam[1]*, Malgorzata Lagisz[1%], Will Cornwell[1%] and Shinichi Nakagawa[1%]

Add affiliation

1 Evolution & Ecology Research Centre and School of Biological, Earth and Environmental Sciences, University of New South Wales, Sydney, Australia

*Correspondence: j.tam@student.unsw.edu.au

%: these authors shared supervision responsibilities equally

Abstract

Background

Taxonomic bias is a known issue within the field of biology, causing scientific knowledge to be unevenly distributed across species. However, a systematic quantification of the research interest that the scientific community has allocated to individual species remains a big data problem. Scalable approaches are needed to integrate biodiversity datasets and bibliometric methods across large numbers of species. The outputs of these analyses are important for identifying understudied species and directing future research to fill these gaps.

Findings

In this study, we used the species *h*-index to quantity the research interest in 7,521 species of mammals. We tested factors potentially driving species *h*-index, by using a Bayesian phylogenetic generalised linear mixed model (GLMM). We found that a third of the mammals had a species *h*-index of zero, while a select few had inflated research interest. Further, mammals with higher species *h*-index had larger body masses, were found in temperate latitudes, had their humans uses documented, including domestication, and were in lower risk IUCN Red List categories. These results surprisingly suggested that critically endangered mammals are understudied. A higher interest in domesticated species suggested that human use is a major driver and focus in mammalian scientific literature.

Conclusion

Our study has demonstrated a scalable workflow and systematically identified understudied species of mammals, as well as identified the likely drivers of this taxonomic bias in the literature. This case study can become a benchmark for future research that asks similar biological and meta-research questions for other taxa.

KEYWORDS: bibliometrics, research bias, meta-research, scientific mapping, research on research, topic modeling

Introduction

Effective conservation of the earth's amazing biodiversity requires sound knowledge of species' biology and ecology, with the addition of adequate communication from scientists [1]. However, such knowledge is often not only missing [2], but also biased. Some species receive disproportionally more research interest while others very little, reflected in scientific publications – known as taxonomic bias [3]. Although taxonomic bias in the scientific literature is prevalent [4,5], there has been little effort to rectify the problem. Even worse, this problem seemed to have become more extreme in the last few decades [6,7]. To work towards reducing the gaps of knowledge in the literature, one first need to understand what is causing such inequality in research interest among species.

Many potential drivers exist for taxonomic bias. For instance, there is a human preference to study and conserve iconic or 'charismatic' taxa, which are usually large mammals such as the African bush elephant (*Loxodonta africana*) and black rhinoceros (*Diceros bicornis*) [8]. Indeed, large mammalian vertebrates are over-represented in the conservation literature [9,10]. Of relevance, the anthropomorphic stimuli hypothesis posits that humans are attracted to species that are more phylogenetically related to us [11]. Such human tendencies likely explain the inflated research effort towards vertebrate taxa [5]. This hypothesis is also related to the reason why we have much (bio)medical research, using rodent model systems such as rats (*Rattus norvegicus* and mice *Mus musculus*), because of our shared physiological traits [12]. Studying species closer to scientists' proximity [5,13], where the animals live in accessible locations, and for economic reasons, such as agriculture and aquaculture research, can also

exacerbate taxonomic bias in the literature. Consequently, these drivers have over time created strong unevenness in the taxonomic distribution of scientific knowledge.

Researchers have investigated such taxonomic bias in the academic literature, but these studies appeared to have two main shortcomings. First, because of the previous difficulties constructing scalable workflows, the coverage of these studies is often not comprehensive. While several studies have quantified species-level bias among plants [14], mammals [15–18], birds [19], fish [20], and amphibians [21] respectively, their sample sizes remain no more than a few hundred species, encompassing only small portions of species in a given taxonomic group. Until now, only two studies have evaluated species-level taxonomic bias for the thousands of species and across multiple clades [4,22]. However, these studies focused solely on species included on the International Union for Conservation of Nature (IUCN) Red List, therefore, potentially failing to provide more comprehensive and holistic understanding of the drivers of taxonomic bias in research.

Second, there are currently no standardized methods to quantify taxonomic bias at the level of individual species. Publication count is one of the most commonly used proxy to gauge taxonomic bias [4,5,7,15,18,20–24]. However, while the total number of publications could capture the total research effort on a given species, it does not capture research interest per se (i.e., how much attention from research community these publications received). A logical alternative would be to use citation count [25], as it captures the total research interest. Nonetheless, high impact papers can easily inflate this number [26] and give a false impression that a species is receiving more interest than in reality. Hirsch's *h*-index [26] kills two birds with

one stone by taking into account both the number of publications and number of citations. So far, there exist only a handful of studies that have adapted the 'species' *h*-index' for measuring and comparing research interests among different species [14,16,17,19,27].

This study seeks to quantify the research interest in mammals, using the species *h*-index [14,16,17,19]. We introduce a workflow demonstrating how to obtain species *h*-index for any species and how to ask relevant meta-science as well as biological questions on research interest. As a case study, we choose the class Mammalia, which consists of over 7,500, species, since they are one of the most well-studied taxonomic groups, with extensive data readily available. Then, we test how our surrogate for research interest, species *h*-index, could be related to the following six potential drivers: 1) body size, 2) location of natural habitat, 3) phylogenetic relatedness, 4) human uses and domestication, 5) (IUCN Red List status, and 6) general interest (encompassing drivers 1 - 5, quantified via Google Trends; see below). We outline our hypothesis and rationale for each potential driver in Table 1.

Methods

Data collection and processing

We first collected a list of mammalian species from the Open Tree of Life (OTL) database [28] using the R package *rotl* version 3.0.12 [29] to create a complete mammalian species list. We removed sub-species from the list and only kept species with binomial names, resulting in 6,952 species. Next, we obtained lists characteristics of mammalian species represented as 7

statistical surrogate of the 6 potential drivers of research interest (Table 1): 1) body mass (n = 5,400; in grams, $\log_{10}$ transformed) 2) median latitude of species range (n = 4,721; obtained from centroids of all occurrence records from GBIF), 3) phylogenetic trees with branch lengths (n = 5,911 [30]), 4) IUCN Red List human use categories (n = 1,472; a binary categorial variable where a species was categorized into at least one of 19 human uses), 5) Wikipedia list of domesticated species (n = 159; a 3-level categorical variable: domesticated, partially domesticated & wild), 6) IUCN Red List status (n = 5,934; an ordinary variable with 5 levels: 'Least Concern, 'Vulnerable', 'Endangered', 'Critically Endangered', and 'Extinct in the Wild' excluding extinct and data deficient; there were no 'Near Threatened' species after combining and cleaning the data, likely removed during synonym matching), and 7) Google Trends index (n = 7,521; see Appendix Fig. S1 for a summary of the data completeness and data processing details and see the Supplementary information). Synonym matching was performed automatically with *rotl::tnrs_match_names()*, before combining the categories and the list from OTL to form 1 dataset. Duplicated names were removed using the functions *unique()* and *duplicate()*. A total of 7,521 unique species remained on the final species list. We obtained the Google Trends index after finalising the list of species names.

Notably, we added higher taxonomic clades to condense the 30 orders to 5 major clades according to molecular tree reconstructions [30,31]. These five high-lever taxa are: 1) Afrotheria representing an African lineage, including sea cows and elephants, 2) Xenarthra representing an American lineage that includes sloths and armadillos, 3) Euarchontoglires representing widely distributed species such as rodents and primates, 4) Laurasiatheria representing species such as whales, carnivores, and bats, and finally 5) Marsupials &

Monotremes representing the non-eutherian mammals. We used these higher taxonomic groupings in visualizations of the results.

For much of data collection and cleaning as well as all statistical analyses (see below), we used the R language version 4.0.2 [32] in the RStudio environment version 1.3.1093 [33]. All processing and analysis scripts were found at GitHub (https://github.com/jessicatytam/biases_in_mammalian_research).

Data sources and species *h*-index

We extracted the bibliometric records from Scopus (data collection on 28 April 2021) and calculated the *h*-index of individual mammal species with the R package *specieshindex* [34]. The package connects to the Scopus, Web of Science, and Bielefeld Academic Search Engine (BASE) literature databases. Using either binomial or genus names, the package can count the number of relevant bibliometric records for each species or genus on each database and extract them for local processing and analysis. Bibliometric information that can be extracted include citation count, publication date, authors, and more. *specieshindex* can then calculate the species *h*-index of individual species applying Hirsch's *h*-index [26]. The *h*-index is defined as the largest number of publications (n) cited a minimum of the same number (n) of times (Appendix Fig. S2). The *h*-index in this scenario quantifies the research interest each individual species has received. The package has also implemented the calculation of other indices, such as the *m*-index, and *h5* index, and plotting functionality.

We used binomial names in Scopus database searches because of the ambiguity and lack of common names for uncommon species. We tackled the issue of species name synonyms by using the Boolean term ‘OR’ between each synonymous binomial name (collected from Open Tree of Life) in the search string. Articles containing binomial names of mammals in their title, abstract, or keywords, were extracted. Since the distribution of h-index was right-skewed with more species having a lower species h-index, we applied the formula $log_{10}(h+1)$ for visualization purposes, but we used the original count data for modeling (see below).

Imputing missing data

The coverage of data is lower for some predictors (Appendix Fig. S1) as a result of synonym matching and cleaning. Since some data was missing for body mass, latitude, and IUCN Red List status (Appendix Fig. S1), we imputed missing values for 5,497 species that were include in the model, to match the shorter length of the phylogenetic tree. We used the multiple imputation approach implemented in the R package *mice* [35]. Multiple imputation creates multiple sets of imputed values before aggregating them to create a single set of data [36]. This is preferred over deletions of data records with missing values, as the latter can result in lowered statistical power and biases in the parameter estimates [37]. We used binomial name, h-index, human use, domestication, and Google Trends index to impute 3 variables with missing values (body mass, latitude, and IUCN Red List status), creating 10 complete datasets for statistical analyses.

Statistical analysis and phylogenetic 'heritability'

We ran three Bayesian phylogenetically controlled Poisson mixed models with the log link function and the additive dispersion term [38], implemented in the R package *MCMCglmm* version 2.33 [39]. The first model followed the predictions stated in the hypotheses (Table 1), and the second was identical except for modeling a linear effect of the IUCN Red List status rather than a quadratic effect. The first two models used the same datasets with the sample size of 5,497 species, and 50 identical phylogenetic trees with branch lengths chosen randomly from Upham et al. [30]. Fifty trees were selected since it is the minimum number of trees needed to account for uncertainties in phylogenetic data [40]. The third model was the same as the first one but with only 5,343 species after removing domesticated and semi-domesticated species (i.e., one less predictor or fixed effect than the first two models; see formulae below). We added this model because (semi-)domesticated species are likely to have inflated species *h*-index values which may not be comparable to those of wild species. We note that in this third model, the quadratic effect of the IUCN Red List status was statistically significant (see Results) and, therefore, we did not run another model with only the linear effect.

We ran 130,000 iterations for the chain with 30,000 burn-ins, drawing 1,000 samples from the imputed data in each iteration, and using a non-informative prior for both fixed and random effects. To obtain more accurate precision of model estimates, we repeated the same model for the 10 imputed datasets and 50 phylogenetic trees, resulting in a total of 500 model runs for each model respectively. The last 100 of the total 1,000 samples of each model were extracted for the calculation of the model results.

In the first model, we used the following predictor variables: body mass value on log_{10} scale (continuous), the absolute value of median latitude (continuous; converted to absolute value for linear distribution), human use (binomial), domestication (ordinal), IUCN Red List status (ordinal), and Google Trends index on (log_{10} + 1) scale (binomial) to model the outcome variable species *h*-index (count), as in the following formula:

$$h \sim log_{10}(Body\ mass) + |Latitude| + \ Human\ use + Domestication$$

$$+ IUCN\ Red\ List\ status + (IUCN\ Red\ List\ status)^2 + log_{10}(Google\ Trends + 1).$$

The second model in the following formula:

$$h \sim log_{10}(Body\ mass) + |Latitude| + Human\ use + Domestication$$

$$+ IUCN\ Red\ List\ status + log_{10}(Google\ Trends + 1).$$

The third model in the following formula (without Domestication):

$$h \sim log_{10}(Body\ mass) + |Latitude| + \ Human\ use$$

$$+ IUCN\ Red\ List\ status + (IUCN\ Red\ List\ status)^2 + log_{10}(Google\ Trends + 1).$$

During our preliminary analysis, we checked for variance inflation factor (VIF) to make sure that the regressors were not correlated to each other. The VIF values ranged between 1.0 – 1.7 (Appendix Table S3). Low VIF values meant that the predictor variables are not co-linear and will not lead to inflated correlations.

We estimated phylogenetic heritability (H^2; [38]) to check for phylogenetic correlations among species, which is equivalent to Pagel's *lambda* (λ). Values of H^2 fall between 0 and 1. The output of the Bayesian model provided the values needed for H^2 calculation using the following formula, from Nakagawa et al. [41]:

$$H^2 = \frac{var(species)}{var(species) + var(overdispersion) + ln\left(1 + \frac{1}{mean(h)}\right)}$$

where *var*(*species*) and *var*(*overdispersion*) are the variance components for phylogenetic effects and the additive overdispersion term, which is equivalent to the residual term in a normal regression and *mean*(*h*) represents the average *h*-index values.

Results

General trends of species' *h*-index across taxa

We calculated the species *h*-index for 7,521 species of mammals in total. A species *h*-index of 0 was common in mammals with 32.26% (n = 2,426; Fig. S4) failing to have even one paper cited one time (Fig. 1). On the other hand, mammals with a species *h*-index of 100 and higher only included 34 species from across 6 orders (Fig. 1a). The median and mean of the h-index for all the species were h_{median} = 2 and h_{mean} = 7.08 respectively. After removing domesticated (and semi-domesticated) species from the dataset (remaining n = 7,360), mammals with a *h*-index of 100 and higher only included 17 species from Carnivora and Primates (7 and 10 species

respectively; Fig. 1b). The median and mean of the species *h*-index without the domesticated mammals are h_{median} = 2 and h_{mean} = 6.16 respectively.

There were also pronounced shifts in research interest through time. Publications in the early 1940s were largely on the orders Hyracoidea (hyraxes), Proboscidea (elephants), Soricomorpha (dissolved paraphyletic taxa of shrews – combined with Erinaceidae to form Eulipotyphla), and Didelphimorphia (opossums) (Fig. 2b). Upon skimming the titles of some articles, we noted that early publications in these species were mostly comparative anatomy studies. In the 1950's, the mammalian literature took on its modern structure, with research focused largely on 6 orders (Fig. 2a) – rodents (Rodentia, 1950-2021 mean = 30.94% of the yearly article count), Primates (1950-2021 mean = 13.98%), bats (Chiroptera, 1950-2021 mean = 11.16%), carnivores (Carnivora, 1950-2021 mean = 11.61%), pigs, sheep, cattle and other even toed ungulates (Artiodactyla, 1950-2021 mean = 11.83%), and whales and dolphins (Cetacea, 1950-2021 mean = 3.15%). Higher species *h*-index was generally associated with larger body sizes (Fig. 4a), intermediate latitudes (Fig. 3, Fig. 4b), more human uses (Fig. 4c) and domestication (Fig. 4d), lower extinction risk (Fig. 4e), and higher general interest (Fig. 4f).

Statistical predictors of species' *h*-index and phylogenetic signal

We included 5,497 species of mammals in the first (Table 2) and second (Table 3), and 5,343 species (after excluding domesticated animals) in the third (Table 4) Bayesian generalized linear mixed model (BGLMM). In all models, body size positively and significantly predicted species *h*-index (Tables 2-4; Fig. 4a). While mammalian diversity was highest in the tropics, species found

here had significantly lower species *h*-indices compared to those in the temperate regions and near the poles, which was again supported in all models (Tables 2-4; Fig. 3; Fig. 4b). Although most mammals had a Google Trends index of 0, species *h*-index significantly increased with the Google Trends index in all models (Tables 2-4; Fig. 4f). Although there seemed to be a hint of u-shape across IUCN Red List status (Fig. 4e), the quadratic effect in the first model was not statically significant (Table 2; see also Appendix Fig. S5 for IUCN Red List statuses not included in the model) whereas this quadratic effect was statistically significant in the third model without domesticated animals (Table 4). All models showed a statistically significant linear decline of species *h*-index with increasing extinction risk (IUCN Red List status). Further, species *h*-index significantly increased with human use in all models (Tables 2-4; Fig. 4c; see Appendix Fig. S6 for all human use categories). The first two models showed that domestication status was a significant positive predictor of species *h*-index (Tables 2-3; Fig. 4d). Finally, phylogenetic signal was present in species *h*-index across all models (Tables 2-4; see Appendix Fig. S7 for the phylogenetic tree).

Discussion

Scientific research is not spread evenly across mammal species: we found strong bias in ‘research interest’ in the literature, quantified by species *h*-index. A small group of species (n = 34 with all species and n = 17 without domestication species) had a species *h*-index above 100, while one-third of the species (n = 2,426 with or without domestication species) received no scientific interest at all (h = 0) (Fig. 1). The modern mammalian literature was dominated by the

orders Rodentia, Primates, Carnivora, Artiodactyla, Chiroptera, and Cetacea (Fig. 2), which resulted in a high value of phylogenetic heritability in the model (H^2 = 64%; see also Appendix Table 2). Overall, our analyses confirmed many of our predictions (Table 1). The bias towards a few orders also appeared in species with high species *h*-indices (Fig. 1) and these commonly found in the high latitudes (Fig. 3). Mammals with high species *h*-indices were more likely to be large, less endangered, and have their utility documented (Fig. 4). These 'research superstars' include farmed animals, pets, and laboratory small mammals, as expected.

Low research interest in endangered small mammals

The relationship between IUCN Red List status and species *h*-index (Fig. 4d) resembled a u-shaped distribution and this trend was statistically significant only in the model without domesticated animals (Table 4). Nonetheless, this model was the most suitable to test for the effect of IUCN Red List status, since most domesticated animals are not endangered and are usually not considered to have high conservation value. In the other models with domesticated species, we found a significant decline (a significant linear effect) in research interest (species *h*-index) with conservation status (i.e., for more endangered mammals). Collectively, these quadratic and linear effects indicate that some endangered species may enjoy higher species *h*-indices, such as the lion (*Panthera leo*) and the orangutan (*Pongo pygmaeus*) (Fig. 1).

We also found that species *h*-index is positively related to increasing body mass (Fig. 4a). These findings could jointly indicate that larger mammals that are less endangered could be attracting more research attention than smaller mammals that are severely endangered. However, since

taxa with larger mammals, such as the big cats and African megafauna, are typically considered more charismatic [8,42] and have higher proportions of threatened species than that of smaller ones [9]. Hence, larger mammals may receive more research interest than smaller mammals, regardless of whether or not they are threatened (Fig. 3b). We found that taxa with smaller mammals in the IUCN Red List categories 'Endangered' and 'Critically Endangered' were likely to have slightly lower species *h*-indices. This indicates a lack of research focus on smaller species, especially those endangered, possibly because they are rarer in the wild and comparatively harder to research.

High research interest with domestication and phylogenetic relatedness

Domesticated species were among the top ranks of mammals with the highest species *h*-indices (Fig. 1a, Fig. 4d). Mammals with human uses documented also had higher species *h*-indices than species with no documented human uses (Fig. 4c). However, some species lack documentation on human their uses because the data on human uses are patchy and not reliable for locally-used species. The strong focus on pets and livestock animals can be explained by their global proximity to humans as well as our needs and preferences. Among all mammals on earth, wild mammals only make up 4% of the total mammalian biomass, while humans and livestock combine to form the other 96% [46], and this corresponds with their widespread occurrence due to the globalization of a small number of animal husbandry systems

[47]. Our need to make our animal use more efficient has clearly driven high volumes of research on these animals.

For example, the literature on cattle or sheep can have contributions and interested readers from all over the world. The broad readership creates academic rewards for researchers and thus positive feedback towards an ever-expanding literature on these animals. In contrast, the research on the grizzled tree-kangaroo, a vulnerable wild species, can only be done on New Guinea and surrounding islands, severely limiting both the pool of potential researchers and potential readers of that research. Thus, not only is it financially and logistically difficult to research grizzled tree-kangaroos, but the readership and academic rewards for doing research in species without any direct human uses are very limited.

We also found phylogenetic signals in species *h*-indices (Tables 2-4), meaning some taxonomic groups usually had higher *h*-indices than others (Fig. 1). Many livestock animals are phylogenetically related, such as the pig (Sus scrofa), the sheep (*Ovis Aries*), and the cow (*Bos taurus*) (Fig 1a), all of which belong to the order Artiodactyla. Furthermore, several primates had relatively high species *h*-indices compared to those from other taxa. Indeed, when we removed the domesticated species, around 65% of the species with *h* = 100 or more were primates (Fig 1b). This finding strongly supports the anthropomorphic stimuli hypothesis [11], where humans tend to be more attracted to species that are phylogenetically similar to us.

Geographical bias towards species in developed countries

We found that mammals with higher species *h*-indices were congregated in clusters centered at the temperate latitudes (Fig. 3, Fig. 4b). Some of these locations – in the USA, Europe, and Australia – are regions with high gross domestic product values, GDP [48], characteristic for developed countries. Not only are scientists in developed countries able to carry out more research activities with better funding, but they have better access to the infrastructure, such as laboratories, transport, and equipment. Higher education is also better implemented in these regions, which is largely lagging in developing countries [49,50]. Developing countries not often require even more research funding to compensate for the scarcity of resources [51]. Since developed countries dominate global publication output [52], the geographical biases revealed in our analyses therefore reflect the research interests of scientists in wealthier countries.

Academic preferences towards certain mammal species also suggest that convenience is often prioritized. This trend is evident in Fig. 3, where species near the tropics had much lower species *h*-indices than those in temperate zones, regardless of their extinction risk. Such preference towards species in the temperate zone is not unique to Mammalia. Scientific literature on species across all taxa, both vertebrates and invertebrates, is biased towards the temperate environment [53]. This is alarming given that 55% of species in the tropics are at risk of local extinctions from climate change, which is higher than that of temperate species, at 39% [54]. At the same time, tropical regions are biodiversity hotspots because of their high species

richness [55]. However, considering that funding in science is often limited, projects that yield the best results with the lowest cost may receive more resources and support.

Potential limitations and future perspectives

This study has four major limitations. Firstly, the data sources included varying lists of mammals with available information, resulting in missing values in some of our predictors (body mass, latitude, and IUCN Red List status) (Appendix Fig. S1). Although this issue was mitigated by imputing values, the results of our study would be more reliable if complete data was available. Further, some species may have been dropped from the analyses as their binomial names were spelled differently from the current consensus name. Although we attempted to incorporate synonyms and remove species that went extinct during the prehistorical and historical times, some synonyms with different spellings and extinct species might still be present in the dataset. This can potentially explain why the sample size of this study is 7,521 species of mammals, much higher than Burgin et al.'s [56] resolved list with only 6,495 species. The issue of unresolved taxonomy is likely going to affect similar studies that attempt to gather high volumes of data for multiple species from other taxa [57].

Secondly, we used the *h*-index [26] as a measurement of research interest since it takes into account both number of publications and numbers of citations. However, there are other similar indices that can be used to quantify research output and influence, including the *h5* index, *m*-index, and *i10* index. The *h5* index is the *h*-index of publications that were published in the past 5 years [58]. The *m*-index is the *h*-index divided by the number of years since the first

publication [26], which directly scales for time (Appendix Fig. S8). Indirectly, the *h*-index can also indicate the time dimension, assuming that more time associates with more publications and more citations. The *i10* index is the total number of articles with 10 or more citations; it is currently used by Google Scholar [59]. Future studies can compare these indices and investigate how they differ with *specieshindex* R package, which can calculate these other indices.

Third, we used species *h*-index here to characterize the distribution of research interest across mammalian species. More research interest does not inform us on the kinds of research that has been done for a given species. Text mining could be used on full-text publications to single out studies with a given topic (e.g., conservation, behaviour, ecology or biomedical use) in future studies, although such endeavor would require access to full-texts.

Finally, although a proxy for general interest in species, presence in Google searches, was a strong and statistically significant predictor of species *h*-index (Fig. 4c, Appendix Table 2), members of the public, in general, are unlikely to use binomial names of species, which we used in this study. We decided against the use of common names for our analyses as many species have multiple common names and many common species names are often used the name of products or companies, and our searches would result in very messy data. Therefore, we require a better proxy for quantifying public interest in different species.

Conclusion

This study has quantified species *h*-index for all available mammalian species as a case study and asked meta-scientific and biological questions. We have elucidated the current patchiness

and biases in the mammalian research landscape using potential drivers of such biases that have been hypothesized before, but perhaps at the largest and finest scale than previously done. More importantly, we have demonstrated potential of addressing meta-research and biological questions by combining available online datasets and species *h*-indices calculated from a bibliometric database. Therefore, future studies can ask a rich set of similar and extended questions to quantify the research landscape of any taxa.

Acknowledgements

We are grateful for comments from Prof. Ian Suthers and A/Prof. Tracy Ainsworth.

Data availability

Additional information is available at the end of the article as Appendix. Complete datasets and code can be retrieved from https://github.com/jessicatytam/biases_in_mammalian_research.

References

1. Rudd MA. How Research-Prioritization Exercises Affect Conservation Policy. *Conserv Biol*. 2011; doi: 10.1111/j.1523-1739.2011.01712.x.

2. Gerlach J, Samways MJ, Hochkirch A, Seddon M, Cardoso P, Clausnitzer V, et al.. Prioritizing non-marine invertebrate taxa for Red Listing. *J Insect Conserv*. 2014; doi: 10.1007/s10841-014-9660-6.

3. Bonnet X, Shine R, Lourdais O. Taxonomic chauvinism. *Trends Ecol Evol*. 2002; doi: 10.1016/S0169-5347(01)02381-3.

4. Donaldson MR, Burnett NJ, Braun DC, Suski CD, Hinch SG, Cooke SJ, et al.. Taxonomic bias and international biodiversity conservation research. Hutchings J, editor. *FACETS*. 2017; doi: 10.1139/facets-2016-0011.

5. Titley MA, Snaddon JL, Turner EC. Scientific research on animal biodiversity is systematically biased towards vertebrates and temperate regions. Schierwater B, editor. *PLOS ONE*. 2017; doi: 10.1371/journal.pone.0189577.

6. Troudet J, Grandcolas P, Blin A, Vignes-Lebbe R, Legendre F. Taxonomic bias in biodiversity data and societal preferences. *Sci Rep*. 2017; doi: 10.1038/s41598-017-09084-6.

7. Rosenthal MF, Gertler M, Hamilton AD, Prasad S, Andrade MCB. Taxonomic bias in animal behaviour publications. *Anim Behav*. 2017; doi: 10.1016/j.anbehav.2017.02.017.

8. Berti E, Monsarrat S, Munk M, Jarvie S, Svenning J-C. Body size is a good proxy for vertebrate charisma. *Biol Conserv*. 2020; doi: 10.1016/j.biocon.2020.108790.

9. Ripple WJ, Wolf C, Newsome TM, Hoffmann M, Wirsing AJ, McCauley DJ. Extinction risk is most acute for the world’s largest and smallest vertebrates. *Proc Natl Acad Sci*. 2017; doi: 10.1073/pnas.1702078114.

10. Seddon PJ, Soorae PS, Launay F. Taxonomic bias in reintroduction projects. *Anim Conserv*. 2005; doi: 10.1017/S1367943004001799.

11. Miralles A, Raymond M, Lecointre G. Empathy and compassion toward other species decrease with evolutionary divergence time. *Sci Rep*. Nature Publishing Group; 2019; doi: 10.1038/s41598-019-56006-9.

12. Bryda EC. The Mighty Mouse: The Impact of Rodents on Advances in Biomedical Research. *Mo Med*. 110:207–112013;

13. Di Marco M, Chapman S, Althor G, Kearney S, Besancon C, Butt N, et al.. Changing trends and persisting biases in three decades of conservation science. *Glob Ecol Conserv*. 2017; doi: 10.1016/j.gecco.2017.01.008.

14. Adamo M, Chialva M, Calevo J, Bertoni F, Dixon K, Mammola S. Plant scientists' research attention is skewed towards colourful, conspicuous and broadly distributed flowers. *Nat Plants*. 2021; doi: 10.1038/s41477-021-00912-2.

15. dos Santos JW, Correia RA, Malhado ACM, Campos-Silva JV, Teles D, Jepson P, et al.. Drivers of taxonomic bias in conservation research: a global analysis of terrestrial mammals. *Anim Conserv*. 2020; doi: 10.1111/acv.12586.

16. Fleming PA, Bateman PW. The good, the bad, and the ugly: which Australian terrestrial mammal species attract most research? *Mammal Rev*. 2016; doi: 10.1111/mam.12066.

17. Robertson PA, McKenzie AJ. The scientific profiles of terrestrial mammals in Great Britain as measured by publication metrics: Publication metrics of mammals in Great Britain. *Mammal Rev*. 2015; doi: 10.1111/mam.12038.

18. Tensen L. Biases in wildlife and conservation research, using felids and canids as a case study. *Glob Ecol Conserv*. 2018; doi: 10.1016/j.gecco.2018.e00423.

19. McKenzie AJ, Robertson PA. Which Species Are We Researching and Why? A Case Study of the Ecology of British Breeding Birds. Margalida A, editor. *PLOS ONE*. 2015; doi: 10.1371/journal.pone.0131004.

20. Ducatez S. Which sharks attract research? Analyses of the distribution of research effort in sharks reveal significant non-random knowledge biases. *Rev Fish Biol Fish*. 2019; doi: 10.1007/s11160-019-09556-0.

21. Schiesari L, Grillitsch B, Grillitsch H. Biogeographic Biases in Research and Their Consequences for Linking Amphibian Declines to Pollution. *Conserv Biol*. 2007; doi: 10.1111/j.1523-1739.2006.00616.x.

22. Trimble MJ, van Aarde RJ. Species Inequality in Scientific Study. *Conserv Biol*. 2010; doi: 10.1111/j.1523-1739.2010.01453.x.

23. da Silva AF, Malhado ACM, Correia RA, Ladle RJ, Vital MVC, Mott T. Taxonomic bias in amphibian research: Are researchers responding to conservation need? *J Nat Conserv*. 2020; doi: 10.1016/j.jnc.2020.125829.

24. Watkins HV, Yan HF, Dunic JC, Côté IM. Research biases create overrepresented "poster children" of marine invasion ecology. *Conserv Lett*. 2021; doi: https://doi.org/10.1111/conl.12802.

25. Wang Z, Zeng J, Meng W, Lohman DJ, Pierce NE. Out of sight, out of mind: public and research interest in insects is negatively correlated with their conservation status. *Insect Conserv Divers*. 2021; doi: 10.1111/icad.12499.

26. Hirsch JE. An index to quantify an individual's scientific research output. *Proc Natl Acad Sci*. 2005; doi: 10.1073/pnas.0507655102.

27. Cox R, McIntyre KM, Sanchez J, Setzkorn C, Baylis M, Revie CW. Comparison of the h-Index Scores Among Pathogens Identified as Emerging Hazards in North America. *Transbound Emerg Dis*. 2016; doi: 10.1111/tbed.12221.

28. McTavish EJ, Hinchliff CE, Allman JF, Brown JW, Cranston KA, Holder MT, et al.. Phylesystem: a git-based data store for community-curated phylogenetic estimates. *Bioinformatics*. 2015; doi: 10.1093/bioinformatics/btv276.

29. Michonneau F, Brown JW, Winter DJ. rotl: an R package to interact with the Open Tree of Life data. Fitzjohn R, editor. *Methods Ecol Evol*. 2016; doi: 10.1111/2041-210X.12593.

30. Upham NS, Esselstyn JA, Jetz W. Inferring the mammal tree: Species-level sets of phylogenies for questions in ecology, evolution, and conservation. Tanentzap AJ, editor. *PLOS Biol*. 2019; doi: 10.1371/journal.pbio.3000494.

31. dos Reis M, Inoue J, Hasegawa M, Asher RJ, Donoghue PCJ, Yang Z. Phylogenomic datasets provide both precision and accuracy in estimating the timescale of placental mammal phylogeny. *Proc R Soc B Biol Sci*. 2012; doi: 10.1098/rspb.2012.0683.

32. R Core Team. R: A language and environment for statistical computing. Vienna, Austria.: R Foundation for Statistical Computing;

33. RStudio Development Team. RStudio: Integrated Development for R. RStudio, PBC, Boston, MA;

34. Tam J. specieshindex: How (scientifically) popular is a given species?

35. van Buuren S van, Groothuis-Oudshoorn K. mice: Multivariate Imputation by Chained Equations in R. *J Stat Softw*. 2011; doi: 10.18637/jss.v045.i03.

36. Nakagawa S. Missing data: mechanisms, methods, and messages. *Ecol Stat Contemp Theory Appl*. Oxford University Press; p. 81–105.

37. Rubin DB. Inference and missing data. *Biometrika*. 1976; doi: 10.1093/biomet/63.3.581.

38. Hadfield JD, Nakagawa S. General quantitative genetic methods for comparative biology: phylogenies, taxonomies and multi-trait models for continuous and categorical characters. *J Evol Biol*. 2010; doi: 10.1111/j.1420-9101.2009.01915.x.

39. Hadfield JD. MCMC Methods for Multi-Response Generalized Linear Mixed Models: The MCMCglmm R Package. *J Stat Softw*. 2010; doi: 10.18637/jss.v033.i02.

40. Nakagawa S, De Villemereuil P. A General Method for Simultaneously Accounting for Phylogenetic and Species Sampling Uncertainty via Rubin's Rules in Comparative Analysis. *Syst Biol*. 2019; doi: 10.1093/sysbio/syy089.

41. Nakagawa S, Johnson PCD, Schielzeth H. The coefficient of determination R2 and intra-class correlation coefficient from generalized linear mixed-effects models revisited and expanded. *J R Soc Interface*. Royal Society; 2017; doi: 10.1098/rsif.2017.0213.

42. Albert C, Luque GM, Courchamp F. The twenty most charismatic species. Maldonado JE, editor. *PLOS ONE*. 2018; doi: 10.1371/journal.pone.0199149.

43. Driscoll CA, Macdonald DW, O'Brien SJ. From wild animals to domestic pets, an evolutionary view of domestication. *Proc Natl Acad Sci*. 2009; doi: 10.1073/pnas.0901586106.

44. Perri AR, Feuerborn TR, Frantz LAF, Larson G, Malhi RS, Meltzer DJ, et al.. Dog domestication and the dual dispersal of people and dogs into the Americas. *Proc Natl Acad Sci*. 2021; doi: 10.1073/pnas.2010083118.

45. vonHoldt BM, Shuldiner E, Koch IJ, Kartzinel RY, Hogan A, Brubaker L, et al.. Structural variants in genes associated with human Williams-Beuren syndrome underlie stereotypical hypersociability in domestic dogs. *Sci Adv*. American Association for the Advancement of Science; 2017; doi: 10.1126/sciadv.1700398.

46. Bar-On YM, Phillips R, Milo R. The biomass distribution on Earth. *Proc Natl Acad Sci*. 2018; doi: 10.1073/pnas.1711842115.

47. Diamond JM. Guns, germs, and steel: the fates of human societies. 1st ed. New York: W.W. Norton & Co;

48. The World Bank, World Development Indicators: GDP (current US$) | Data. https://data.worldbank.org/indicator/NY.GDP.MKTP.CD (2019). Accessed 2021 Jun 27.

49. Harris E. Building scientific capacity in developing countries. *EMBO Rep*. 2004; doi: 10.1038/sj.embor.7400058.

50. Vose PB, Cervellini A. Problems of scientific research in developing countries. *IAEA Bull*. 25:37–401983;

51. van Helden P. The cost of research in developing countries. *EMBO Rep*. 2012; doi: 10.1038/embor.2012.43.

52. Jaffe K, Horst E ter, Gunn LH, Zambrano JD, Molina G. A network analysis of research productivity by country, discipline, and wealth. *PLOS ONE*. Public Library of Science; 2020; doi: 10.1371/journal.pone.0232458.

53. Culumber ZW, Anaya-Rojas JM, Booker WW, Hooks AP, Lange EC, Pluer B, et al.. Widespread Biases in Ecological and Evolutionary Studies. *BioScience*. 2019; doi: 10.1093/biosci/biz063.

54. Wiens JJ. Climate-Related Local Extinctions Are Already Widespread among Plant and Animal Species. Barnosky A, editor. *PLOS Biol*. 2016; doi: 10.1371/journal.pbio.2001104.

55. Ceballos G, Ehrlich PR. Global mammal distributions, biodiversity hotspots, and conservation. *Proc Natl Acad Sci*. 2006; doi: 10.1073/pnas.0609334103.

56. Burgin CJ, Colella JP, Kahn PL, Upham NS. How many species of mammals are there? *J Mammal*. 2018; doi: 10.1093/jmammal/gyx147.

57. Remsen D. The use and limits of scientific names in biological informatics. *ZooKeys*. 2016; doi: 10.3897/zookeys.550.9546.

58. Crotty D. Other Metrics: beyond the Impact Factor. *Eur Heart J*. 2017; doi: 10.1093/eurheartj/ehx446.

59. Noruzi A. Impact Factor, h-index, i10-index and i20-index of Webology. *Webology*. 13:1–42016;

60. Wilman H, Belmaker J, Simpson J, Rosa C de la, Rivadeneira MM, Jetz W. EltonTraits 1.0: Species-level foraging attributes of the world's birds and mammals. *Ecology*. 2014; doi: https://doi.org/10.1890/13-1917.1.

61. Global Biodiversity Information Facility: GBIF. https://www.gbif.org/ (2021). Accessed 2021 May 2.

62. Borges R, Machado JP, Gomes C, Rocha AP, Antunes A. Measuring phylogenetic signal between categorical traits and phylogenies. Hancock J, editor. *Bioinformatics*. 2019; doi: 10.1093/bioinformatics/bty800.

63. Sulzner K, Fiorello C, Ridgley F, Garelle D, Deem SL. Conservation medicine and One Health in zoos: Scope, obstacles, and unrecognized potential. *Zoo Biol*. 2021; doi: 10.1002/zoo.21572.

64. Tuck N. Animals in Moral Limbo: How Literary Pigs May Help Lab-Generated Ones. *Animals*. 2020; doi: 10.3390/ani10040629.

65. IUCN: The IUCN Red List of Threatened Species. Version 2021-1. IUCN Red List Threat. Species. https://www.iucnredlist.org/en (2021). Accessed 2021 May 2.

66. Wikipedia. List of domesticated animals. Wikipedia.

67. Chamberlain S. rredlist: “IUCN” Red List Client.

68. Colléony A, Clayton S, Couvet D, Saint Jalme M, Prévot A-C. Human preferences for species conservation: Animal charisma trumps endangered status. *Biol Conserv*. 2017; doi: 10.1016/j.biocon.2016.11.035.

69. Google: Google Trends. Google Trends. https://trends.google.com/trends/?geo=AU (2021). Accessed 2021 May 2.

70. Massicotte P, Eddelbuettel D. gtrendsR: Perform and Display Google Trends Queries.

Tables

TABLE 1 Details of hypotheses. We predicted that species *h*-index can be influenced by body sizes, location of natural habitat, phylogeny, human uses and domestication, demography, and general interest.

Potential driver	Hypothesis and rationale	Statistical surrogate	Data source
Size of species	We predict that higher body masses correlate with higher species *h*-index. Larger mammals, i.e. megafaunal species such as elephants and rhinoceroses, receive more research interest because they are generally considered as more 'charismatic' [8,42].	Body mass (transformed with $\log_{10}$)	Wilman et al. [60]
Location of natural habitat	We predict that species found in temperate latitudes have higher species *h*-index. Mammals near the temperate zones attract more research interest as more researchers originate from these areas, such as	Median latitude	GBIF [61]

	North America, Europe, Australia, New Zealand, and southern Africa [5]. Thus, mammals whose natural habitat are within these regions are better studied.		
Phylogenetic relatedness	We predict that there are phylogenetic signals present in the dataset. Mammals that are more phylogenetically related receive similar species *h*-index because related species share similar traits that may influence the propensity of researchers to study members of a given clade [62]. Furthermore, species closer to humans will be over-represented in species with high *h*-index values [11].	Branch lengths of phylogenetic tree	Upham et al. [30]
Human use & Domestication	We predict that mammals with more human uses and domesticated mammals have higher species *h*-index. Some examples of human uses include transportation (e.g. horses and elephants),	IUCN Red List human use categories & Wikipedia list of domesticated species	IUCN Red List [65], & Wikipedia [66]

	companionship (e.g. cats and dogs), food products (e.g. sheep and cattle), etc. Lab animals (e.g. rabbits and rodents) are likely to receive most research interest since the main purpose of keeping these animals is for scientific research [63,64].		
Demography	We predict a u-shaped distribution of species *h*-index, where species in the 'Least Concern' and 'Critically Endangered' categories receive higher species *h*-index. Previous studies showed no correlations between the mammals' IUCN Red List status and their research interest [16,17,19].	IUCN Red List status	IUCN Red List [65]; cleaned with *rredlist* [67]
General interest	We predict that more general interest correlates with higher species *h*-index. Research and general interests are highly correlated since we tend to be more attracted to 'charismatic' species, such as lions	Google Trends index	Google Trends [69]; extracted with *gtrendsR* [70]

	and elephants [42], and are more willing to donate for their conservation causes [68], resulting in more research interest.

TABLE 2 Summary of statistical results from the Bayesian generalized linear mixed model (BGLMM). The distributions here follow the distributions stated in the hypothesis.

**Estimate**	**Mean**	**95% Credible Interval (CI)**
Fixed effects		
Intercept	1.333	-0.082, 2.751
$\log_{10}$(Body mass)	0.094	0.025, 0.157
Latitude (absolute value)	0.022	0.019, 0.025
IUCN Red List status (1st degree polynomial)	-16.463	-19.509, -13.367
IUCN Red List status (2nd degree polynomial)	2.356	-0.227, 5.083
Human use	0.277	0.175, 0.378
Domestication status	-0.377	-0.549, -0.205

$\log_{10}$(Google Trends)	0.490	0.458, 0.522
Random effects		
Phylogeny	1.592	1.076, 2.222
Non-phylogeny	0.806	0.745, 0.868
Phylogenetic heritability (H^2)	0.636 (*0.641)	0.000, 0.659 (*0.515, 0.659)

*Phylogenetic signal after removing 1124 species (20.4%) from the tree that showed no signals.

TABLE 3 Summary of statistical results from the Bayesian generalized linear mixed model (BGLMM). The distributions here follow the distributions stated in the hypothesis, except with IUCN Red List status set to a linear relationship.

**Estimate**	**Mean**	**95% Credible Interval (CI)**
Fixed effects		
Intercept	1.720	0.301, 3.149
$\log_{10}$(Body mass)	0.093	0.025, 0.156
Latitude (absolute value)	0.022	0.019, 0.025
IUCN Red List status	-0.255	-0.303, -0.206
Human use	0.273	0.172, 0.376
Domestication status	-0.381	-0.552, -0.208
$\log_{10}$(Google Trends)	0.491	0.459, 0.524

Random effects		
Phylogeny	1.594	1.075, 2.218
Non-phylogeny	0.806	0.745, 0.870
Phylogenetic heritability (H^2)	0.636 (*0.641)	0.000, 0.659 (*0.515, 0.660)

*Phylogenetic signal after removing 1124 species (20.4%) from the tree that showed no signals.

TABLE 4 Summary of statistical results from the Bayesian generalized linear mixed model (BGLMM). The distributions here follow the distributions stated in the hypothesis, except we removed domestication status and domesticated and partially-domesticated species.

**Estimate**	**Mean**	**95% Credible Interval (CI)**
Fixed effects		
Intercept	0.165	-1.142, 1.483
$\log_{10}$(Body mass)	0.101	0.031, 0.165
Latitude (absolute value)	0.022	0.019, 0.025
IUCN Red List status (1st degree polynomial)	-16.696	-19.810, -13.637
IUCN Red List status (2nd degree polynomial)	2.606	0.016, 5.327
Human use	0.280	0.178, 0.383

$\log_{10}$(Google Trends)	0.501	0.468, 0.533
Random effects		
Phylogeny	1.562	1.050, 2.189
Non-phylogeny	0.821	0.760, 0.886
Phylogenetic heritability (H^2)	0.624 (*0.630)	0.000, 0.650 (*0.508, 0.650)

*Phylogenetic signal after removing 1124 species (21.0%) from the tree that showed no signals.

Figures

(a)

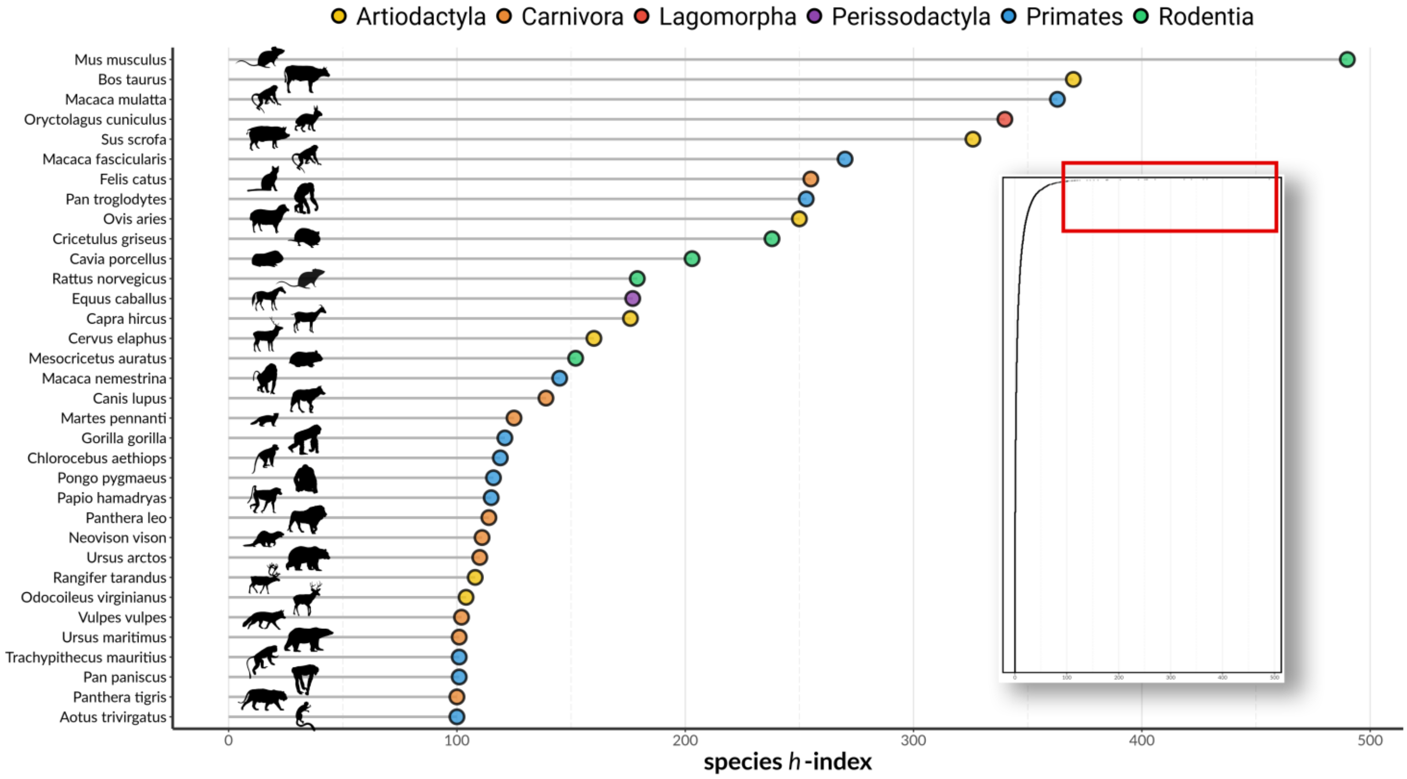
Artiodactyla Carnivora Lagomorpha Perissodactyla Primates Rodentia
Mus musculus
Bos taurus
Macaca mulatta
Oryctolagus cuniculus
Sus scrofa
Macaca fascicularis
Felis catus
Pan troglodytes
Ovis aries
Cricetulus griseus
Cavia porcellus
Rattus norvegicus
Equus caballus
Capra hircus
Cervus elaphus
Mesocricetus auratus
Macaca nemestrina
Canis lupus
Martes pennanti
Gorilla gorilla
Chlorocebus aethiops
Pongo pygmaeus
Papio hamadryas
Panthera leo
Neovison vison
Ursus arctos
Rangifer tarandus
Odocoileus virginianus
Vulpes vulpes
Ursus maritimus
Trachypithecus mauritius
Pan paniscus
Panthera tigris
Aotus trivirgatus
0
100
200
300
400
500
species h-index

(b)

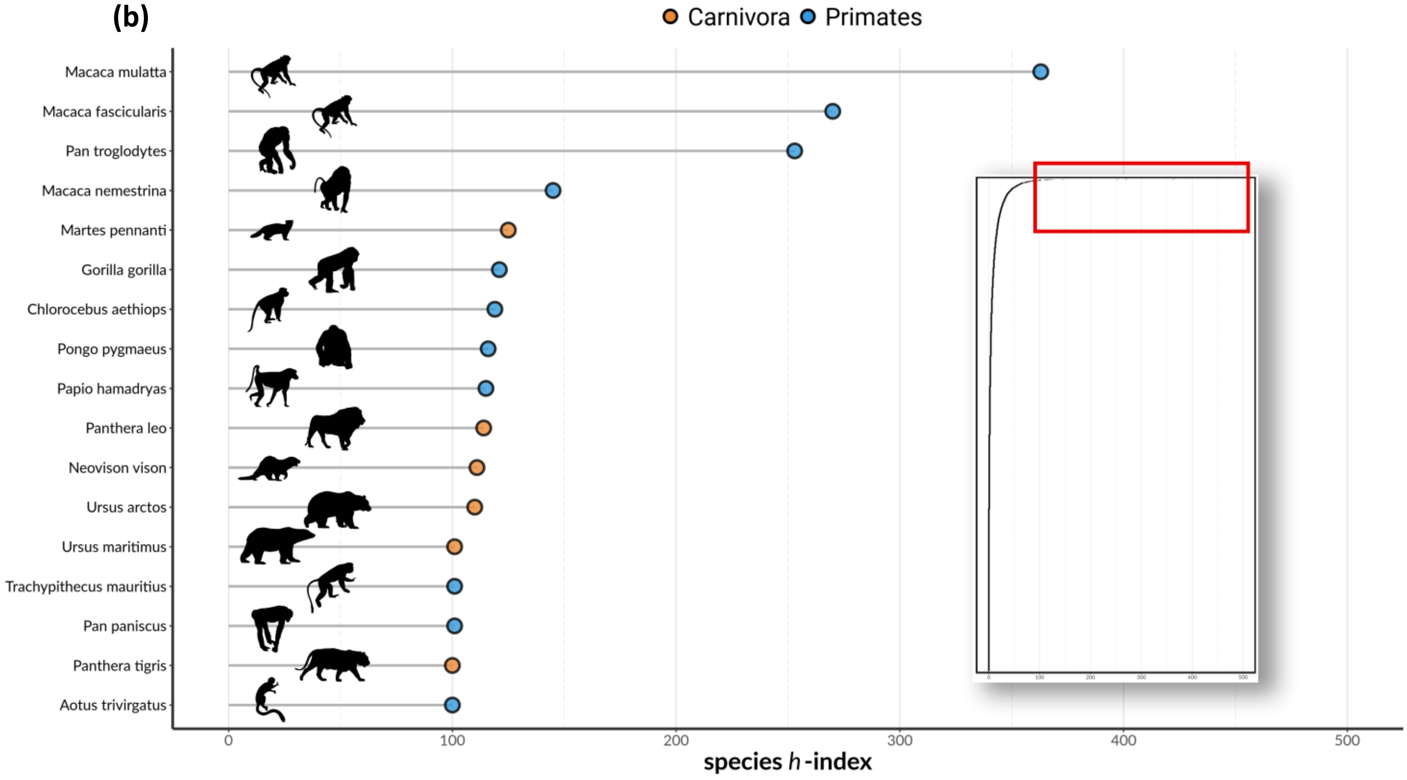
Carnivora Primates
Macaca mulatta
Macaca fascicularis
Pan troglodytes
Macaca nemestrina
Martes pennanti
Gorilla gorilla
Chlorocebus aethiops
Pongo pygmaeus
Papio hamadryas
Panthera leo
Neovison vison
Ursus arctos
Ursus maritimus
Trachypithecus mauritius
Pan paniscus
Panthera tigris
Aotus trivirgatus
0
100
200
300
400
500
species h-index

FIGURE 1 Species *h*-index of mammals. Plot (a) shows 34 mammals with h = 100 or more, representing 6 different orders marked by dots of different colours. Figure in the inset shows the distribution of species *h*-index of all mammals, with the species scoring above h = 100 or more marked by the red box. Plot (b) also shows the mammals with h = 100 or more, but removes domesticated species, with 17 species left.

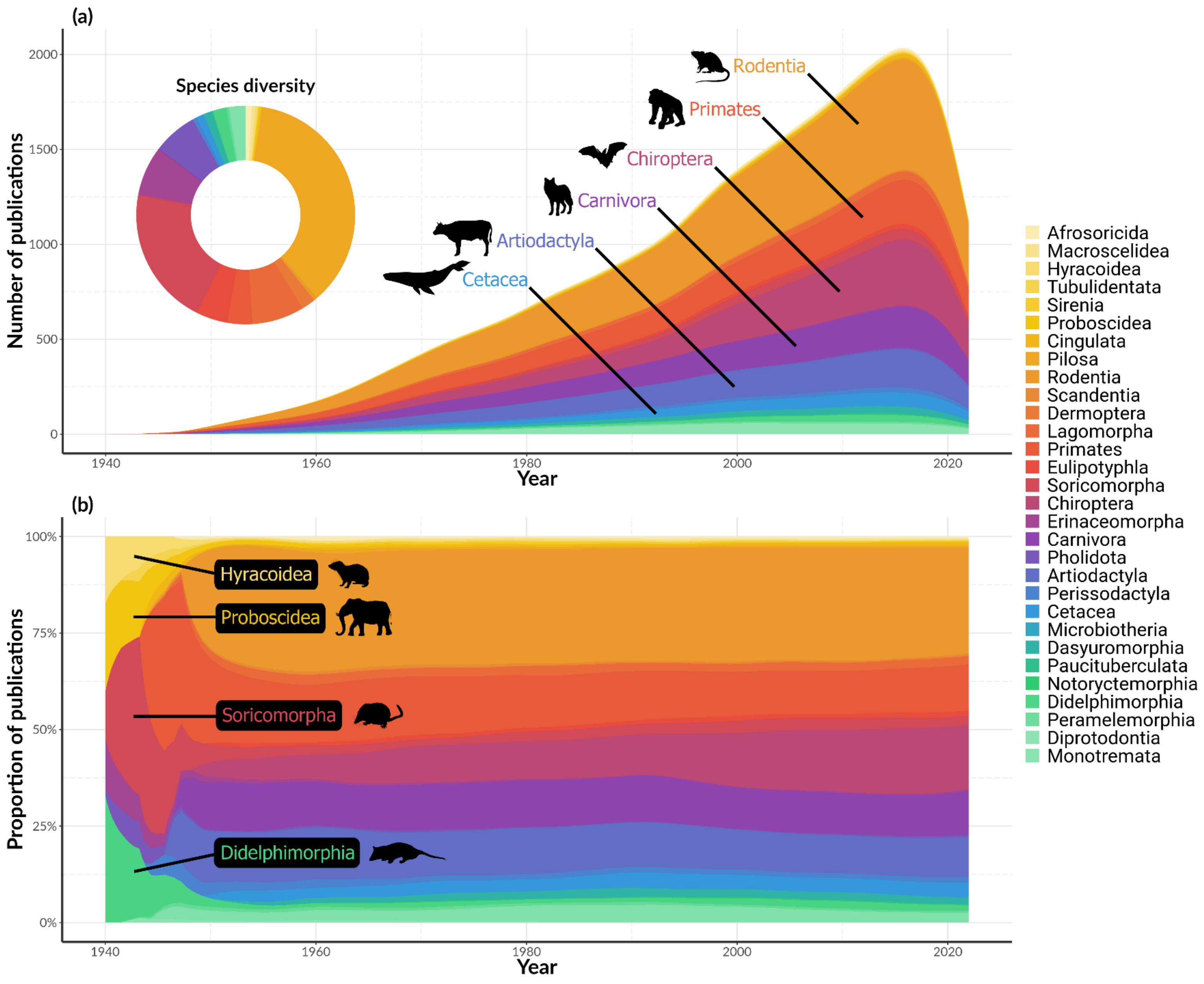

FIGURE 2 The changes in mammalian literature from 1940 to 28 April 2021. (a) The number of publications per year for 30 mammalian orders and the proportion of species per order from the collated mammalian dataset represented by the doughnut chart, and (b) change in the frequency of publications on 30 mammalian orders present in the dataset. Total number of mammalian species analysed is 7,521.

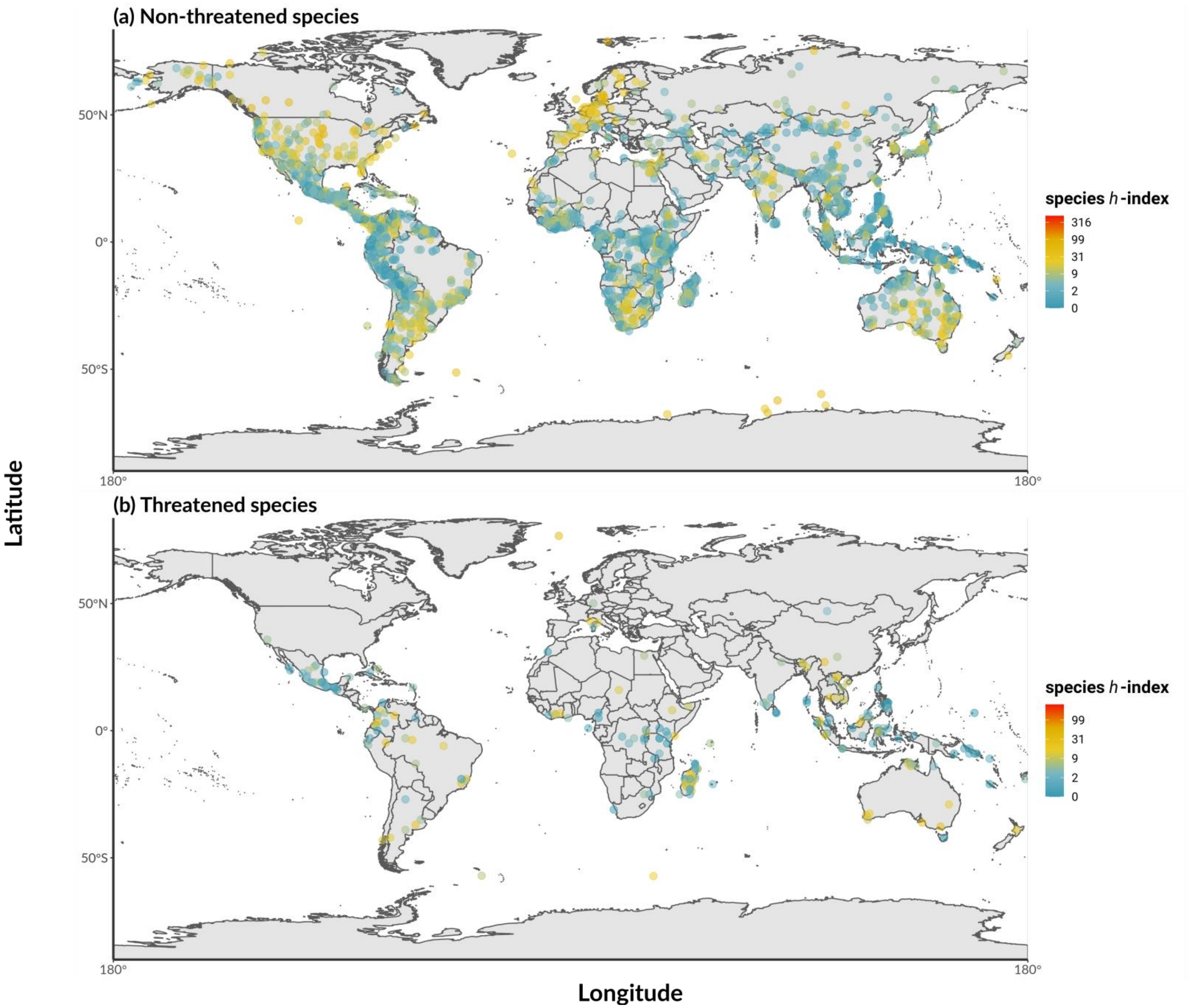

FIGURE 3 Centroids of global distributions of 4,435 mammalian species. (a) The distribution of non-threatened species listed as ‘Least Concern’. (b) The distribution of threatened species listed as ‘Vulnerable’, ‘Endangered’, ‘Critically Endangered’, and ‘Extinct in the Wild’. The species’ corresponding *h*-index values are illustrated by dot colour.

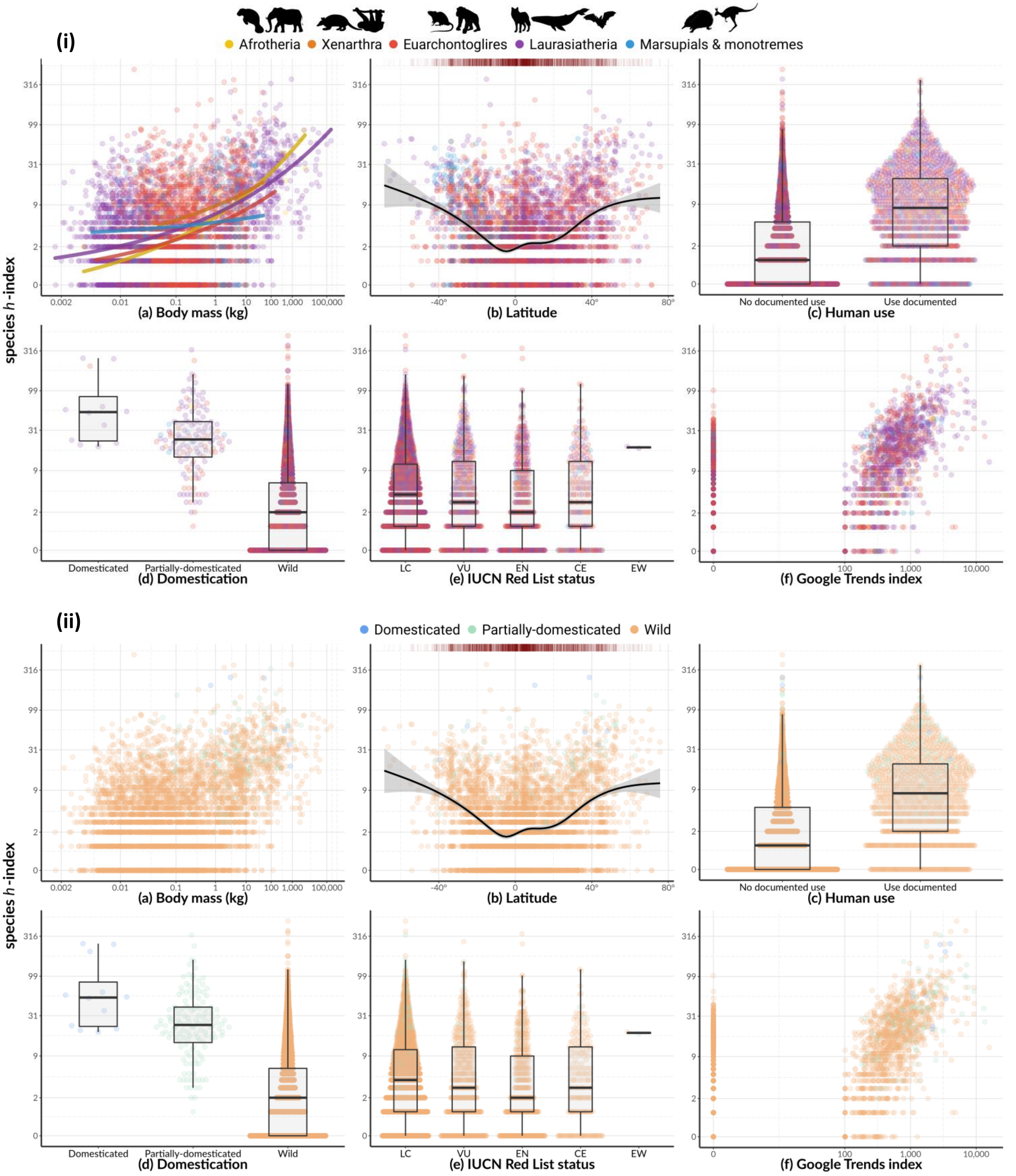
(i)
Afrotheria
Xenarthra
Euarchontoglires
Laurasiatheria
Marsupials & monotremes
species h-index
(a) Body mass (kg)
(b) Latitude
(c) Human use
No documented use
Use documented
(d) Domestication
Domesticated
Partially-domesticated
Wild
(e) IUCN Red List status
LC
VU
EN
CE
EW
(f) Google Trends index
(ii)
Domesticated
Partially-domesticated
Wild
species h-index
(a) Body mass (kg)
(b) Latitude
(c) Human use
No documented use
Use documented
(d) Domestication
Domesticated
Partially-domesticated
Wild
(e) IUCN Red List status
LC
VU
EN
CE
EW
(f) Google Trends index

FIGURE 4 Relationship between predictor variables and species *h*-index values. **(i)** (a) Species average body mass (n = 5,158 species, fitted curves represent 50% quantile for each clade), (b) Median latitude of species geographical distribution (n = 4,435 species, fitted curve from generalised additive model (GAM) with shaded grey area representing 95% confidence interval; density bar on top of the plot illustrates the number of species at each latitude). (c) Human use categories (n = 7,521, $n_{\text{No documented}}$ use = 6,124, and $n_{\text{Use documented}}$ = 1,397). (d) Domestication status (n = 7,521 species, $n_{\text{Domesticated}}$ = 12, $n_{\text{Partially-domesticated}}$ = 136, and n_{Wild} = 7373). (e) IUCN Red List status (n = 5,244 species, $n_{\text{Least Concern}}$ = 3152, $n_{\text{Vulnerable}}$ = 530, $n_{\text{Endangered}}$ = 512, $n_{\text{Critically Endangered}}$ = 208, and $n_{\text{Extinct in the Wild}}$ = 2). (f) Google Trends Index summed for each species (n = 7,521 species, $n_{\text{Google Trends Index} > 0}$ = 1,323, and $n_{\text{Google Trends Index} = 0}$ = 6,124 species). Box plots in (c), (d), and (e) show the median, 25th and 75th percentiles, and lower and upper extremes. **(ii)** showing the same data as (i), but each species is coloured according to their domestication status.

Click here to access/download
Supplementary Material
Additional_files_2.pdf

Click here to access/download;Personal Cover;Manuscript_comments.pdf

GIGA-D-21-00396

Quantifying research interests in 7,521 mammalian species with h-index: a case study

Jessica Tam; Malgorzata Lagisz; Will Cornwell; Shinichi Nakagawa

GigaScience

Dear Mx Tam,

Your manuscript "Quantifying research interests in 7,521 mammalian species with h-index: a case study" (GIGA-D-21-00396) has been assessed by our reviewers. Although it is of interest, we are unable to consider it for publication in its current form. The reviewers have raised a number of points which we believe would improve the manuscript and may allow a revised version to be published in GigaScience.

In particular, please carefully consider the more major points raised in review # 2 (which is a group review by Louise McRae together with 3 colleagues). As the reviewers point out, many mammals are used for biomedical research or as domesticated species, and thus unsurprisingly generate a large research output - and this may confound your conclusions regarding conservation issues. I recommend that you take a closer look at this, as suggested by the reviewers; likewise with respect to the temporal component in the data.

Reply 1

Thank you for reviewing our manuscript and making suggestions. We have used these suggestions to improve the manuscript. We re-ran some models and double-checked the data to clarify on the points raised below that were causing confusion. Below, we have addressed the points raised by the reviewers (in blue) with quoted text from the manuscript (in purple) and highlighted the major changes (in yellow).

Both reviews mention a couple of other methodological points that should be clarified.

The reports, together with any other comments, are below. Please also take a moment to check our website at https://www.editorialmanager.com/giga/ for any additional comments that were saved as attachments.

If you are able to fully address these points, we would encourage you to submit a revised manuscript to GigaScience. Once you have made the necessary corrections, please submit online at:

https://www.editorialmanager.com/giga/

If you have forgotten your username or password please use the "Send Login Details" link to get your login information. For security reasons, your password will be reset.

Please include a point-by-point within the 'Response to Reviewers' box in the submission system. Please ensure you describe additional experiments that were carried out and include a detailed rebuttal of any criticisms or requested revisions that you disagreed with. Please also ensure that your revised manuscript conforms to the journal style, which can be found in the Instructions for Authors on the journal homepage. If the data and code has been modified in the revision process please be sure to update the public versions of this too.

The due date for submitting the revised version of your article is 17 Apr 2022.

I look forward to receiving your revised manuscript soon.

Best wishes,

Hans Zauner

GigaScience

www.gigasciencejournal.com

Reviewer reports:

Reviewer #1: I have finished the review of the manuscript entitled "Quantifying research interests in 7,521 mammalian species with h-index: a case study". I found this to be a great contribution that advances current knowledge and provides a much needed approach to study issues related to taxonomic bias and their implications for research and conservation.

In particular, I can see two major contributions: 1) the study adds to current knowledge of taxonomic biases in mammals, highlighting the problems in quantifying and studying such biases and delivering new results that highlight how widespread such biases are and what they imply for basic research and conservation of biodiversity. 2) the study provides a very well-thought and flexible approach, together with code to perform similar analyses and to expand them. I can see this approach to set a new standard on how to investigate taxonomic biases for future studies.

The manuscript is very well-written and I enjoyed reading it very much. All conclusions are supported by the data and all statistical analyses are clear and fit well the questions asked. In particular, I really enjoyed how the authors structured the Discussion, which clearly put the results in a broad context and that is easy to understand. The authors also acknowledged all potential limitations of the approach used very honestly, provided several alternatives to account for them in future studies and proposed many interesting and important suggestions to advance the field.

I do not have any major suggestion that could add to the quality and importance of the study. I have few minor suggestions, listed below, to improve small parts of the manuscript.

Reply 2

Thank you for the kind comments!

I have been studying taxonomic harmonization quite a lot lately and I was very glad to see that the authors are also concerned of the issues with taxonomic backbones. In particular, the authors acknowledge in the Discussion that taxonomic backbones are not fixed and that they change over time and have sometimes unresolved synonyms. I think this is a very well-written paragraph and address the major potential issues with taxonomic backbones. I believe these potential biases to be largely negligible for this study, considering its aims and the thorough workflow that seemed to have been put in this step. I would, however, expand briefly in the Method section on how taxonomic names of species were obtained in order to give more details of this step. This would help to understand better how this was performed, which may also help many readers to perform similar

analyses. Specifically, names were obtained from the Open Tree of Life (OTL) accessed through the R package rcol (Catalogue of Life). I would suggest to briefly expand here on how this search was performed and how synonyms were resolved, e.g. by stating which functions were used and how synonyms were combined (e.g. is there a direct function to retrieve an accepted name, or was this done with a custom function?). I would also include the version of rcol used and the access time (year/month) to OTL (through COL?), as taxonomic backbones can change over time and results from queries can also change depending on the version of the package.

Reply 3

We have added much more details in the methods section, including the names of packages and functions that we used during data cleaning. We further explained how taxonomic harmonisation will likely not affect the results in this study significantly. Our revised text is quoted below:

Methods

"We first collected a list of mammalian species from the Open Tree of Life (OTL) database [28] using the R package *rotl* version 3.0.12 [29] to create a complete mammalian species list. We removed sub-species from the list and only kept species with binomial names, resulting in 6,952 species. Next, we obtained lists of characteristics of mammalian specie represented as 7 statistical surrogates of the 6 potential drivers of research interest (Table 1): 1) body mass (n = 5,400; in gram, $\log_{10}$ transformed) 2) median latitude of species range (n = 4,721; obtained from centroids of all occurrence records from GBIF), 3) phylogenetic trees with branch lengths (n = 5,911 [30]), 4) IUCN Red List human use categories (n = 1,472; a binary categorial variable where a species was categorized into at least one of 19 human uses), 5) Wikipedia list of domesticated species (n = 159; a 3-level categorical variable: domesticated, partially domesticated & wild), 6) IUCN Red List status (n = 5,934; an ordinary variable with 5 levels: 'Least Concern, 'Vulnerable', 'Endangered', 'Critically Endangered', and 'Extinct in the Wild', excluding extinct and data deficient; there were no 'Near Threatened' species after combining and cleaning the data, likely removed during synonym matching), and 7) Google Trends index (n = 7,521; see Appendix Fig. S1 for a summary of the data completeness and data processing details and see the Supplementary information). Synonym matching was performed automatically with *rotl::tnrs_match_names()*, before combining the categories and the list from OTL to form 1 dataset. Duplicated names were removed using the functions *unique()* and *duplicate()*. A total of 7,521 unique species remained on the final species list. We obtained the Google Trends index after finalising the list of species names."

Discussion

"This study has four major limitations. Firstly, the data sources included varying lists of mammals with available information, resulting in missing values in some of our predictors (body mass, latitude, and IUCN Red List status) (Appendix Fig. S1). Although this issue was mitigated by imputing values, the results of our study would be more reliable if complete data was available. Further, some species may have been dropped from the analyses as their binomial names were spelled differently from the current consensus name. Although we attempted to incorporate synonyms and remove species that went extinct during the prehistorical and historical times, some synonyms with different spellings and extinct species might still be present in the dataset. This, can potentially explain why the sample size of this study is 7,521 species of mammals, much higher than Burgin et al.'s [56] resolved list with only 6,495 species. The issue of unresolved taxonomy is likely going to affect similar studies that attempt to gather high volumes of data for multiple species from other taxa [57]."

In the first paragraph of the Results, the authors give some interesting statistics, namely how many species have an h-index = 0 and how many species have an h-index >= 100. These are very intuitive statistics that tell much to the reader. I wonder if including also the median would be a nice addition, as it will give an additional metric that is also intuitive to understand and that would complement the two already given.

Reply 4

Thank you for this great suggestion. We have added the median to the text. We also provided the mean to provide more context. Since we ran a third model (without domesticated species), we've also added the mean and median without the domesticated species. The results of the third model was similar to that of the existing first and second model. Changes in the text is quoted below:

"We calculated the species *h*-index for 7,521 species of mammals in total. A species h-index of 0 was common in mammals, with 32.26% (n = 2,426; Fig. S4) failing to have even one paper cited one time (Fig. 1). On the other hand, mammals with a species *h*-index of 100 and higher only included 34 species from across 6 orders (Fig. 1a). The median and mean of the *h*-index for all the species were h_{median} = 2 and h_{mean} = 7.08 respectively. After removing domesticated species from the dataset (remaining n = 7,360), mammals with a *h*-index of 100 and higher only included 17 species from Carnivora and Primates (7 and 10, respectively; Fig. 1b). The median and mean of the species h-index without the domesticated mammals were h_{median} = 2 and h_{mean} = 6.16, respectively."

Below is a new plot of the species with a h-index of 100 and above:

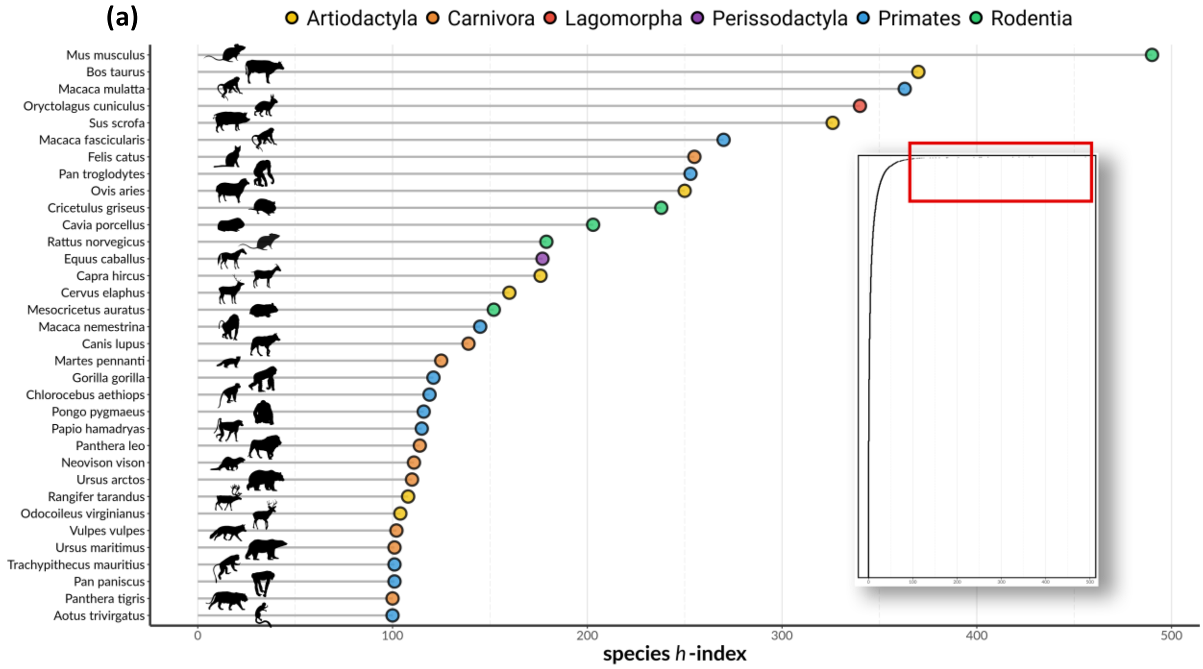

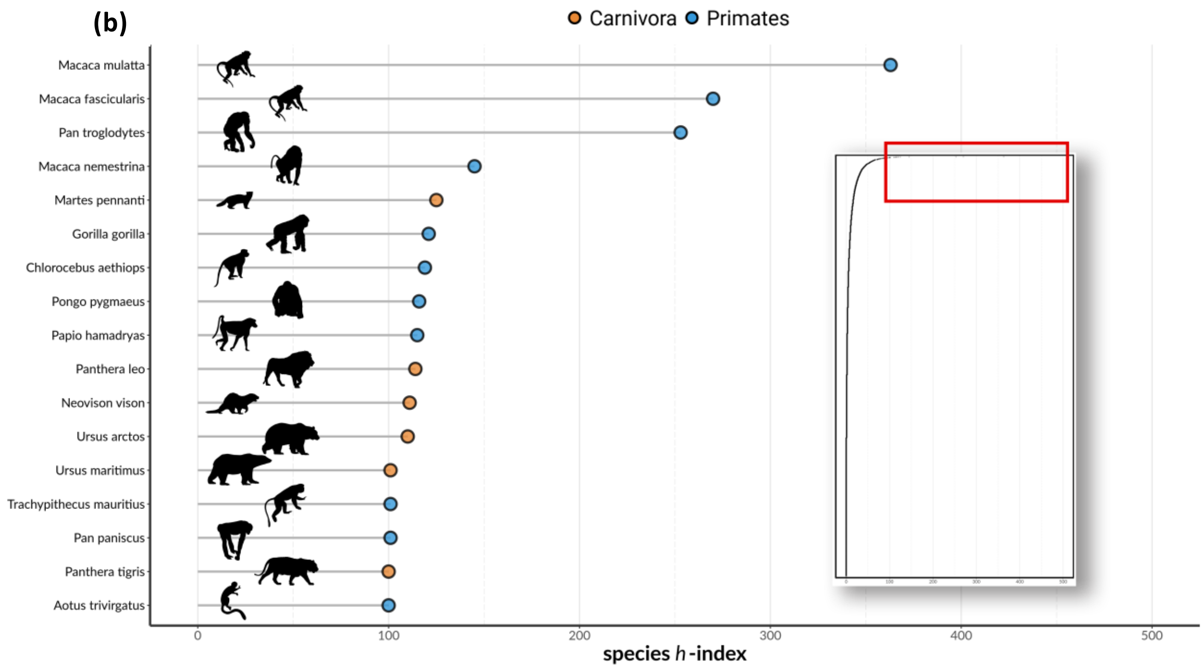

FIGURE 1 Species h-index of mammals. Plot (a) shows 34 mammals with h = 100 or more, representing 6 different orders marked by dots of different colours. Figure in the inset shows the distribution of species h-index of all mammals, with the species scoring above h = 100 or more marked by the red box. Plot (b) also shows the mammals with h = 100 or more, but removes domesticated species, with 17 species left.

In the Results, I think 'Appendix Table 2' was moved to the main text and should be 'Table 2'.

Reply 5

Thank you for pointing this out! It has now been fixed.

I found Figure 3 (map plot with species h-index and IUCN status) to be a bit hard to follow. In particular, I cannot distinguish very well among IUCN status, which are here represented by different

point sizes. As points often overlap, the size is sometimes hard to understand. Perhaps faceting it by IUCN status could help, maybe also grouping VU, EN, CE into a 'threaten' class to avoid having five maps.

Reply 6

Thank you for the suggestion. We have replaced the previous figure with the one shown below. This new figure separates the LC species (a) from the ones in threatened classes (b), with a new figure caption.

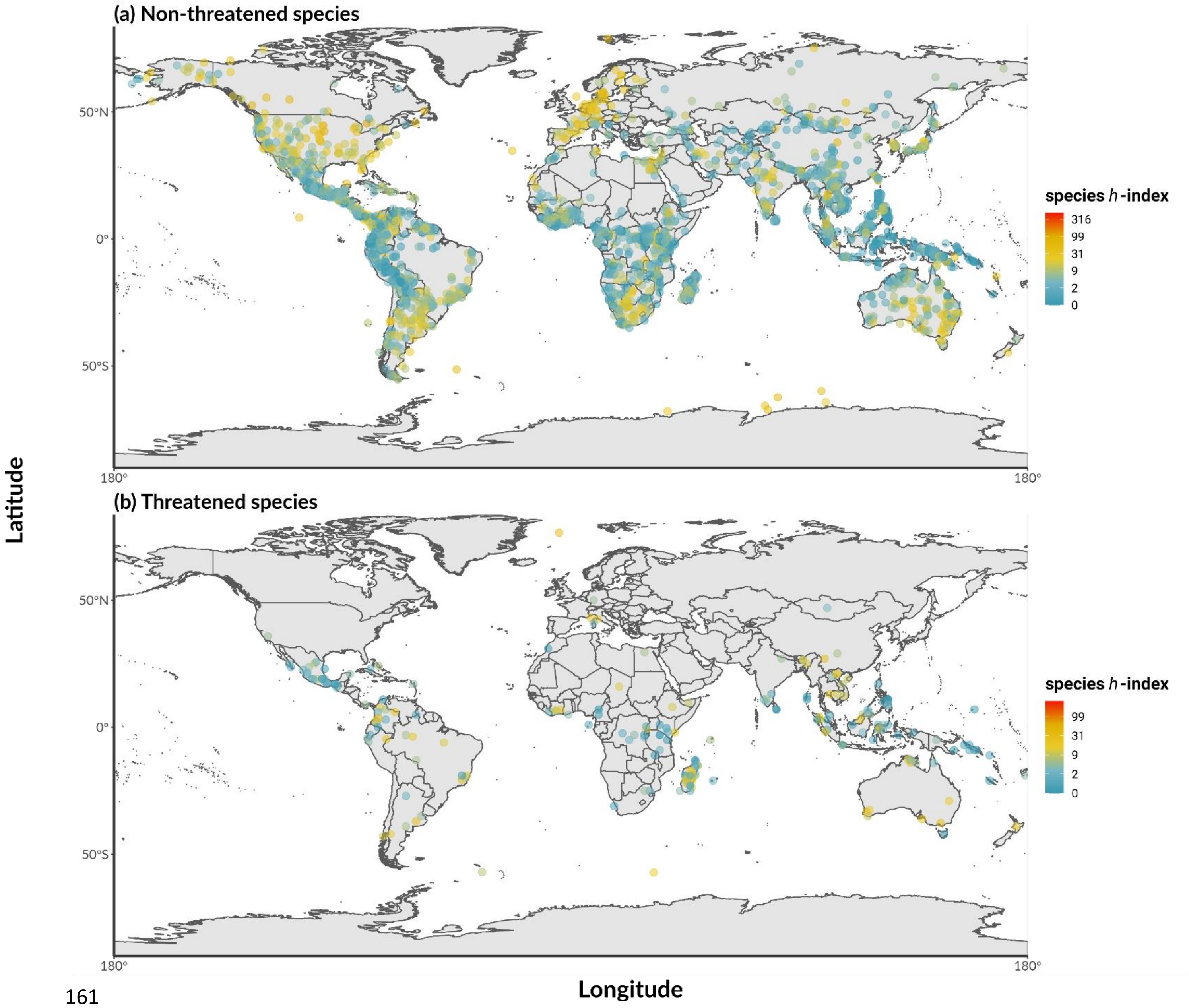

FIGURE 3 Centroids of global distributions of 4,435 mammalian species. (a) The distribution of non-threatened species listed as 'Least Concern'. (b) The distribution of threatened species listed as 'Vulnerable', 'Endangered', 'Critically Endangered', and 'Extinct in the Wild'. The species' corresponding *h*-index values are illustrated by dot colour.

Sincerely,

Emilio Berti

German Centre for Integrative Biodiversity Research (iDiv) Halle-Jena-Leipzig

Theory in Biodiversity Science

04103 Leipzig, Germany

emilio.berti@idiv.de

Reviewer #2: Tam et al tackle an interesting and important topic of taxonomic bias in research and extend the research by including more species than in previous studies and use a different metric for assessing the amount of research. They use bibliometric records to assemble a large data set of mammal publications and assess research interest using the h-index as a metric. They identify 6 potential drivers affecting research interest and used a Bayesian generalised linear mixed model to assess the importance of these factors. They found higher h-index was associated with larger body size, temperate latitudes, lower extinction risk, with higher human uses. Bringing together large data sets in this way is hugely valuable and challenging, but we had a few concerns and questions on the process and conclusions drawn.

General points

Narrative around research interest

We were confused by the focus of the manuscript at times. The results seem to have a broad scope of any research but the introduction and discussion seemed to take a conservation slant to some degree. Given that a lot of the research is on mammals used for biomedical research, pets and other domesticated species (human focussed), the conservation narrative does not seem like it should be as relevant in explaining the patterns observed (species focussed). For example, the prevalence of certain mammal species (e.g. Mus musculus) in the literature arguably has more to do with what research gets funded, what the priorities are in terms of medical research, rather than the interest of the individual researcher themselves. However, the argument about charismatic species and logistics of geography (needing to conduct fieldwork) would seem to only be relevant to wild species and not domesticated ones. Would it be useful to divide the data set up to scrutinise some of the findings further e.g. separate by broad discipline or by removing domesticated species and/or ones which are commonly used in research? You mention in limitations about looking at topics of research if full text was available, so perhaps the first suggestion would be hard, but perhaps journal title could help with that. That would be interesting to see the biases in the remaining species which would give a better picture of biases in conservation and ecological research, if that is the main interest of the authors.

Reply 7

We removed domesticated species from our list (remaining n = 7,360) and re-ran the model with the same variables as in Table 2 (see the results table below). While the majority of the results are qualitatively similar, we now found IUCN Red List status (2nd degree polynomial) to be statistically significant. The table below has now been incorporated into the main text as Table 4.

Estimate	Mean	95% Credible Interval (CI)
Fixed effects		
Intercept	0.165	-1.142, 1.483
$\log_{10}$(Body mass)	0.101	0.031, 0.165
Latitude (absolute value)	0.022	0.019, 0.025
IUCN Red List status (1st degree polynomial)	-16.696	-19.810, -13.637
IUCN Red List status (2nd degree polynomial)	2.606	0.016, 5.327
Human use	0.280	0.178, 0.383
$\log_{10}$(Google Trends)	0.501	0.468, 0.533
Random effects		
Phylogeny	1.562	1.050, 2.189
Non-phylogeny	0.821	0.760, 0.886
Phylogenetic heritability (H^2)	0.624 (*0.630)	0.000, 0.650 (*0.508, 0.650)
*Phylogenetic signal after removing 1124 species (21.0%) from the tree that showed no signals.		

We have therefore rewritten our methods and results sections. We have included the names of the packages and functions that we used during data cleaning. We further explained how taxonomic harmonisation will likely not affect the results in this study significantly. Quoted as follows:

Methods

Statistical analysis and phylogenetic 'heritability'

"We ran three Bayesian phylogenetically controlled Poisson mixed models with the log link function and the additive dispersion term [38], implemented in the R package *MCMCglmm* version 2.33 [39]. The first model followed the predictions stated in the hypotheses (Table 1), and the second was identical except for modeling a linear effect of the IUCN Red List status rather than a quadratic effect. The first two models used the same datasets with the sample size of 5,497 species and 50 identical phylogenetic trees with branch lengths chosen randomly from Upham et al. [30]. Fifty trees were selected since it is the minimum number of trees needed to account for uncertainties in phylogenetic data [40]. The third model was the same as the first one but with only 5,343 species after removing domesticated and semi-domesticated species (i.e., one less predictor or fixed effect than the first two models; see formulae below). We added this model because (semi-)domesticated species are likely to have inflated species h-index values, which may not be comparable to those of wild species. We note that in this third model, the quadratic effect of the IUCN Red List status was statistically significant (see Results) and, therefore, we did not run another model with only the linear effect.

We ran 130,000 iterations for the chain with 30,000 burn-ins, drawing 1,000 samples from the imputed data in each iteration, and using a non-informative prior for both fixed and random effects.

To obtain more accurate precision of model estimates, we repeated the same model for the 10 imputed datasets and 50 phylogenetic trees, resulting in a total of 500 model runs for each model respectively. The last 100 of the total 1,000 samples of each model were extracted for the calculation of the model results.

In the first model, we used the following predictor variables: body mass value on $\log_{10}$ scale (continuous), the absolute value of median latitude (continuous; converted to absolute value for linear distribution), human use (binomial), domestication (ordinal), IUCN Red List status (ordinal), and Google Trends index on ($\log_{10}$ + 1) scale (binomial) to model the outcome variable species h-index (count), as in the following formula:

$$h \sim log_{10}(Body\ mass) + |Latitude| + \ Human\ use + Domestication + IUCN\ Red\ List\ status + (IUCN\ Red\ List\ status)^2 + log_{10}(Google\ Trends + 1).$$

The second model in the following formula:

$$h \sim log_{10}(Body\ mass) + |Latitude| + Human\ use + Domestication + IUCN\ Red\ List\ status + log_{10}(Google\ Trends + 1).$$

The third model in the following formula (without Domestication):

$$h \sim log_{10}(Body\ mass) + |Latitude| + \ Human\ use + IUCN\ Red\ List\ status + (IUCN\ Red\ List\ status)^2 + log_{10}(Google\ Trends + 1).$$

During our preliminary analysis, we checked for variance inflation factor (VIF) to make sure that the regressors were not correlated to each other. The VIF values ranged between 1.0 – 1.7 (Appendix Table S3). Low VIF values meant that the predictor variables are not co-linear and will not lead to inflated correlations.

We estimated phylogenetic heritability (H^2; [38]) to check for phylogenetic correlations among species, which is equivalent to Pagel's lambda (λ). Values of H^2 fall between 0 and 1. The output of the Bayesian model provided the values needed for H^2 calculation using the following formula, from Nakagawa et al. [41]:

$$H^2 = \frac{var(species)}{var(species) + \ var(overdispersion) + \ ln\left(1 + \frac{1}{mean(h)}\right)}$$

where *var(species)* and *var(overdispersion)* are the variance components for phylogenetic effects and the additive overdispersion term, which is equivalent to the residual term in a normal regression and *mean(h)* represents the average h-index values."

Results

General trends of species' h-index across taxa

"We calculated the species h-index for 7,521 species of mammals in total. A species *h*-index of 0 was common in mammals with 32.26% (n = 2,426; Fig. S4) failing to have even one paper cited one time (Fig. 1). On the other hand, mammals with a species h-index of 100 and higher only included 34 species from across 6 orders (Fig. 1a). The median and mean of all the species h-index were h_{median} = 2 and h_{mean} = 7.08 respectively. After removing the domesticaed (and semi-domesticated) species (remaining n = 7,360), mammals with a h-index of 100 and higher only included 17 species from

Carnivora and Primates (7 and 10 species, respectively; Fig. 1b). The median and mean of the species h-index without the domesticated mammals are h_{median} = 2 and h_{mean} = 6.16, respectively.

There were also pronounced shifts in research interest through time. Publications in the early 1940s were largely on the orders Hyracoidea (hyraxes), Proboscidea (elephants), Soricomorpha (dissolved paraphyletic taxa of shrews – combined with Erinaceidae to form Eulipotyphla), and Didelphimorphia (opossums) (Fig. 2b). Upon skimming the titles of some articles, we noted that early publications in these species were mostly comparative anatomy studies. In the 1950's, the mammalian literature took on its modern structure, with research focused largely on 6 orders (Fig. 2a) – rodents (Rodentia, 1950-2021 mean = 30.94% of the yearly article count), Primates (1950-2021 mean = 13.98%), bats (Chiroptera, 1950-2021 mean = 11.16%), carnivores (Carnivora, 1950-2021 mean = 11.61%), pigs, sheep, cattle and other even toed ungulates (Artiodactyla, 1950-2021 mean = 11.83%), and whales and dolphins (Cetacea, 1950-2021 mean = 3.15%). Higher species h-index was generally associated with larger body sizes (Fig. 4a), intermediate latitudes (Fig. 3, Fig. 4b), more human uses (Fig. 4c) and domestication (Fig. 4d), lower extinction risk (Fig. 4e), and higher general interest (Fig. 4f)."

Statistical predictors of species' h-index and phylogenetic signal

"We included 5,497 species of mammals in the first (Table 2) and second (Table 3), and 5,343 species (after excluding domesticated animals) in the third (Table 4) Bayesian generalized linear mixed models (BGLMM). In all models, body size positively and significantly predicted species h-index (Tables 2-4; Fig. 4a). While mammalian diversity was highest in the tropics, species found here had significantly lower species h-indices compared to those in the temperate regions and near the poles, which was again supported in all models (Tables 2 – 4; Fig. 3; Fig. 4b). Although most mammals had a Google Trends index of 0, species h-index significantly increased with the Google Trends index in all models (Tables 2-4; Fig. 4f). Although there seemed to be a hint of U-shape across IUCN Red List status (Fig. 4e), the quadratic effect in the first model was not statically significant (Table 2; see also Appendix Fig. S5 for IUCN Red List statuses not included in the model), whereas this quadratic effect was statistically significant in the third model without domesticated animals (Table 4). All models showed a statistically significant linear decline of species h-index with increasing extinction risk (IUCN Red List status). Further, species h-index significantly increased with human use in all models (Tables 2 – 4; Fig. 4c; see Appendix Fig. S6 for all human use categories). The first two models showed that domestication status was a significant positive predictor of species h-index (Tables 2 – 3; Fig. 4d). Finally, phylogenetic signal was present in species *h*-index across all models (Tables 2 – 4; see Appendix Fig. S7 for the phylogenetic tree)."

Temporal component

Have the authors considered how time may influence their results? Firstly, species which have historically been well studied have had longer to accrue more papers/citations and therefore a higher h-index so could including publications from that far back introduce some bias? Also there have been changes in disciplines from over time particularly within ecology and conservation which could influence the research focus from good model species for ecology (e.g. predator-prey dynamics) to species which are important from a conservation perspective. Is it useful/possible to put year of publication in the model to address this? You mention in limitations that other similar indices, which have temporal components, could be used in the analyses, but do not explain why the h-index was chosen in this study, instead of these other similar indices.

Reply 8

While we did not take the element of time into consideration, the *h*-index takes time into consideration, assuming that more time equates to more published research. To check whether this assumption is true, we calculated and plotted the *m*-index (see figure below, incorporated into the manuscripts as Appendix Fig. S8). In line with Figure 1 from the manuscript, it has many of the same top-ranking species, including species from the clades Artiodactyla, Carnivora, Lagomorpha, Perissodactyla, Primates, and Rodentia. We have also clarified that the *h*-index is indicative of time in the discussion section, as quoted below:

"The *m*-index is the *h*-index divided by the number of years since the first publication [26], which directly scales for time. Indirectly, the *h*-index can also indicate the time dimension, assuming that more time associates with more publications and more citations."

Time was not explicitly considered in our analyses, as it is not part of our original set of questions (rather than post-hoc questions). We have suggested future studies to do comparisons between various research metrics, as quoted below:

"Future studies can compare these indices and investigate how they differ with *specieshindex* R package, which can calculate these other indices."

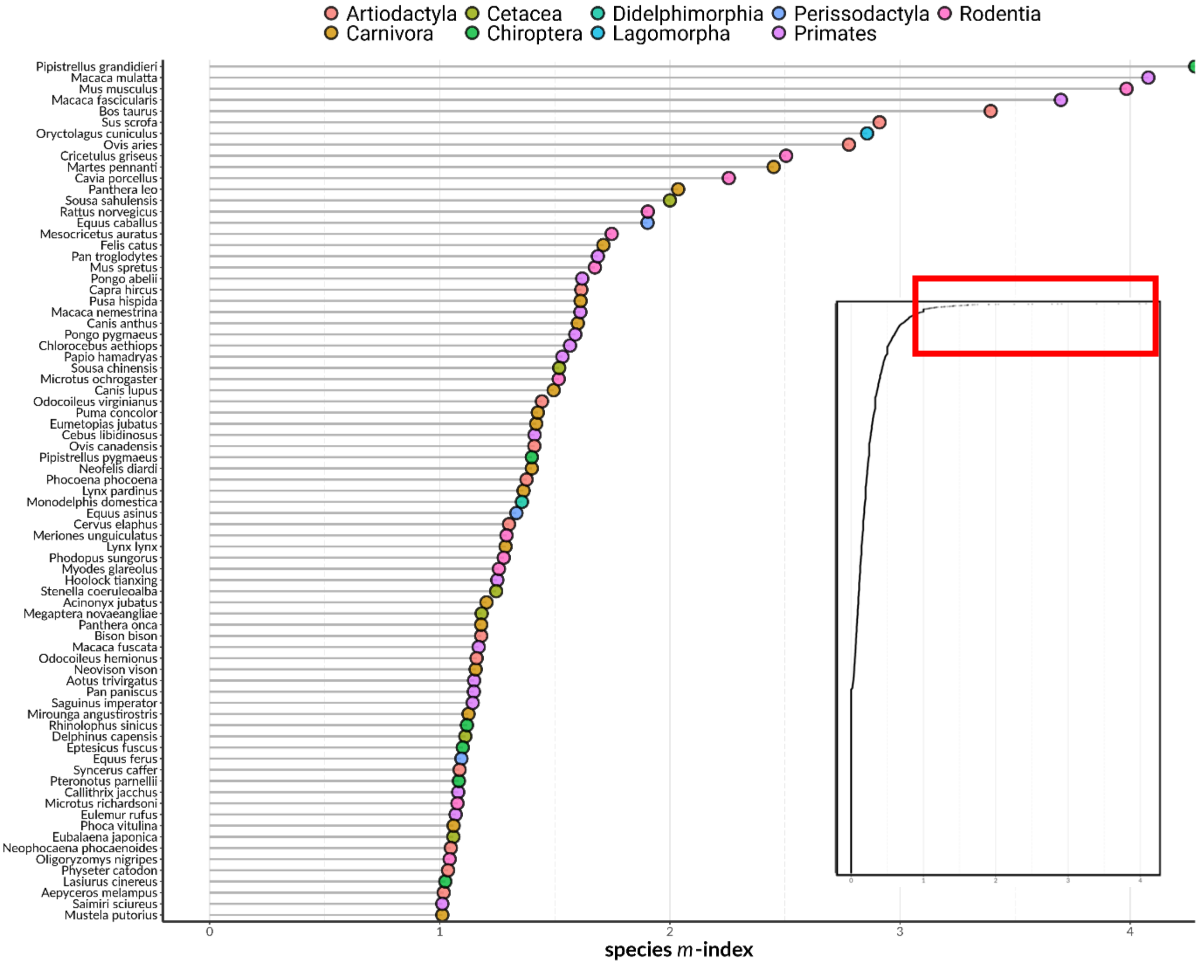

Finally, the Red List status is a snapshot and a fairly recent one so how might that affect the conclusions drawn about extinction risk? Could it be more appropriate to look at papers in the last 30 years or so to make conclusions about red list status and conservation generally?

Reply 9

After checking our data, we found that most (72.7%) of publications were published since 1990. While there is still a sizable portion of papers published before the 90s, we believe that the results of our analyses are likely dominated by recent research trends.

Workflow documentation

We found some aspects missing or hard to follow e.g. number of species in the data set, how the species searches were done and a note on how the workflow could be scalable. Why did the authors use GBIF for the centroids when actual species ranges are available from the Red List, or are these less comprehensive in terms of species coverage?

Reply 10

Number of species: see our Reply 3

Scalable workflow: What we meant by scalability is that in Introduction, we mentioned that previous studies on research interest in species dealt with relatively small numbers of species. Here, we clearly scaled up addressing this limitation. We did not mean that we establish a general workflow for any future research. But we suggested that using a similar workflow, “future studies can ask a rich set of similar and extended questions to quantify the research landscape of any taxa.” In our concluding sentence.

The use of GBIF: We have checked the differences between the latitudes provided by IUCN and GBIF. We found a strong correlation with the correlation coefficient equal to 0.95645. Since the two sets of data are very similar, picking one or the other will likely not affect the results significantly. The coordinate points that differ are for small number of species that have wide ranges, e.g. humpback whale (*Megaptera novaeangliae*), and Blasius’ horseshoe bat (*Rhinolophus blasii*). Given high similarity between GBIF and Red List species ranges, we have kept our GBIF derived locations for the analyses as this was part of our original dataset.

Specific comments:

Findings

Line 24: The authors may need to be more circumspect with regards to the conclusion about human use. Whilst it is accurately defined as ‘use documented’ versus ‘no use documented’, the Red List information recorded is not comprehensive and that particular field on use has been a recommended one so whilst it is a very believable result, it might need to be caveated that those with no use documented are not necessarily not used.

Reply 11

Good point. We’ve elaborated on this point slightly in the discussion section as follows:

“Domesticated species were among the top ranks of mammals with the highest species *h*-indices (Fig. 1a, Fig. 4d). Mammals with human uses documented also had higher species *h*-indices than species with no documented human uses (Fig. 4c). However, some species lack documentation on their human uses because the data on human uses are patchy and not reliable for locally-used species. The strong focus on pets and livestock animals can be explained by their global proximity to humans as well as our needs and preferences. Among all mammals on earth, wild mammals only make up 4% of the total mammalian biomass, while humans and livestock combine to form the other 96% [46], and this corresponds with their widespread occurrence due to the globalization of a

small number of animal husbandry systems [47]. Our need to make our animal use more efficient has clearly driven high volumes of research on these animals."

Introduction

Line 49: this is an interesting hypothesis to introduce here but it isn't picked up again in the discussion. It seems like it should be relevant to come back to

Reply 12

Thank you for pointing this out, this is certainly an important hypothesis that supports our results. We have now added a paragraph in the discussion outlining our results in regards to phylogenetic relatedness as quoted below:

"We also found phylogenetic signal in species *h*-indices (Tables 2-4), meaning some taxonomic groups usually had higher h-indices than others (Fig. 1). Many livestock animals are phylogenetically related, such as the pig (Sus scrofa), the sheep (Ovis Aries), and the cow (Bos taurus) (Fig 1a), all of which belong to the order Artiodactyla. Furthermore, several primates had relatively high species h-indices compared to those from other taxa. Indeed, when we removed the domesticated species, around 65% of the species with h = 100 or more were primates (Fig 1b). This finding strongly supports the anthropomorphic stimuli hypothesis [11], where humans tend to be more attracted to species that are phylogenetically similar to us."

Method

Line 94 and Line 105: how did the number of species change from 6,952 to 7,521? This is implied later in the manuscript as being partly to do with synonyms but should be explicit here in the methods. It is a larger number than known mammal species totals so we think that should be introduced here.

Reply 13

Apologies for the confusion. We have included the names of the packages and functions that we used during data cleaning. We further explained how taxonomic harmonisation will likely not affect the results in this study significantly. Quoted below:

Methods

We first collected a list of mammalian species from the Open Tree of Life (OTL) database [28] using the R package *rotl* version 3.0.12 [29] to create a complete mammalian species list. We removed sub-species from the list and only kept species with binomial names, resulting in 6,952 species. Next, we obtained lists characteristics of mammalian species represented as 7 statistical surrogate of the 6 potential drivers of research interest (Table 1): 1) body mass (n = 5,400; in grams, $\log_{10}$ transformed) 2) median latitude of species range (n = 4,721; obtained from centroids of all occurrence records from GBIF), 3) phylogenetic trees with branch lengths (n = 5,911 [30]), 4) IUCN Red List human use categories (n = 1,472; a binary categorial variable where a species was categorized into at least one of 19 human uses), 5) Wikipedia list of domesticated species (n = 159; a 3-level categorical variable: domesticated, partially domesticated & wild), 6) IUCN Red List status (n = 5,934; an ordinary variable with 5 levels: 'Least Concern, 'Vulnerable', 'Endangered', 'Critically Endangered', and 'Extinct in the Wild' excluding extinct and data deficient; there were no 'Near Threatened' species after combining and cleaning the data, likely removed during synonym matching), and 7) Google Trends index (n = 7,521; see Appendix Fig. S1 for a summary of the data completeness and data processing details and see the Supplementary information). Synonym matching was performed automatically with

rotl::tnrs_match_names(), before combining the categories and the list from OTL to form 1 dataset. Duplicated names were removed using the functions *unique()* and *duplicate()*. A total of 7,521 unique species remained on the final species list. We obtained the Google Trends index after finalising the list of species names.

Discussion

This study has four major limitations. Firstly, the data sources included varying lists of mammals with available information, resulting in missing values in some of our predictors (body mass, latitude, and IUCN Red List status) (Appendix Fig. S1). Although this issue was mitigated by imputing values, the results of our study would be more reliable if complete data was available. Further, some species may have been dropped from the analyses as their binomial names were spelled differently from the current consensus name. Although we attempted to incorporate synonyms and remove species that went extinct during the prehistorical and historical times, some synonyms with different spellings and extinct species might still be present in the dataset. This can potentially explain why the sample size of this study is 7,521 species of mammals, much higher than Burgin et al.'s [56] resolved list with only 6,495 species. The issue of unresolved taxonomy is likely going to affect similar studies that attempt to gather high volumes of data for multiple species from other taxa [57].

Line 102: Was Near Threatened excluded or just missed from the list? If it was not included then this needs an explanation

Reply 14

We have clarified in the methods sections, that there were no NT species left after cleaning up the data. This is likely due to the species being removed after synonym matching:

"6) IUCN Red List status (n = 5,934; an ordinary variable with 5 levels: 'Least Concern, 'Vulnerable', 'Endangered', 'Critically Endangered', and 'Extinct in the Wild' excluding extinct and data deficient; there were no 'Near Threatened' species after combining and cleaning the data, likely removed during synonym matching)."

Line 132: in which fields were the binomial names searched?

Reply 15

We have now clarified this in the methods section, as quoted below:

"Articles containing binomial names of mammals in their title, abstract, or keywords, were extracted."

Line 140: we were surprised that the Red List status was only 69.72% complete when all mammals have been assessed. Is there an explanation for this?

Reply 16

We suspect that this is an issue caused by dropping species with names spelt differently. Nonetheless, we imputed missing status data using the data that was available. Although the results could differ, they likely are not going to vary significantly. We have clarified this in the discussion as one of our limitations:

"Firstly, the data sources included varying lists of mammals with available information, resulting in missing values in some of our predictors (body mass, latitude, and IUCN Red List status) (Appendix Fig. S1). Although this issue was mitigated by imputing values, the results of our study would be more reliable if complete data was available. Further, some species may have been dropped from the analyses as their binomial names were spelled differently from the current consensus name. Although we attempted to incorporate synonyms and remove species that went extinct during the prehistorical and historical times, some synonyms with different spellings and extinct species might still be present in the dataset. This can potentially explain why the sample size of this study is 7,521 species of mammals, much higher than Burgin et al.'s [56] resolved list with only 6,495 species. The issue of unresolved taxonomy is likely going to affect similar studies that attempt to gather high volumes of data for multiple species from other taxa [57]."

Results

Line 195-6: Given the early focus of these studies is there value in looking at more recent trends in the data set so as to not be skewed by changes in disciplines? Also, how did the authors deduce this – were there fields from the publication search that were used to do this?

Reply 17

After checking our data, we found that most of the publications (83.3%) were published fairly recently, after the 1980s. Hence, the dataset should still reflect recent trends and it is not likely that the older publications are skewing our results.

We were able to deduce the contents of earlier papers by skimming their titles. We have further clarified this in the manuscript text:

"Upon skimming the titles of some articles, we noted that early publications in these species were mostly comparative anatomy studies. "

Line 188-190: this seemed like a huge result and possibly more important than some others. Incredible that almost a third of species have an h-index of 0, implying that there were no publications about these species that had been cited even once in the time period covered. It is unclear in the method which fields were searched. Could older papers that are scanned and so the main text is not searchable contribute to this? If only title and abstract are searched this should perhaps be mentioned as a limitation as some species could be missed.

Reply 18

We searched for the binomial names in the title, abstract, and keywords, but not the main text. We excluded the main text to avoid the papers that would mention species that were not the study's main species. We also did not screen the main texts since we had thousands of publications included in this study. Doing whole text search for all the papers would take too long. We have added the following sentence in the methods section:

"Articles containing binomial names of mammals in their title, abstract, or keywords, were extracted."

Discussion

Line 242-244: Can this be concluded if there was no significant interaction in the model between body size and Red List status?

Reply 19

We believe that we could as both predictors were tested; that means larger and less endangered mammals are more likely to have higher species h index values than smaller and more endangered species. We believe that our sentence does not necessarily imply the interaction, and even if it may, we could still speculate (see our wording). We note that we did not test for any interactions in this study, since it was not part of our initial questions. Also, interactions without hypotheses may lead to spurious findings.

Line 245-247: This seems slightly contradictory to the previous conclusion - this one suggests that large mammals (a higher proportion of which are threatened with extinction) have received more research interest

Reply 20

Apologies for the confusion, we have reworded the paragraph as follows:

"We also found that species h-index is positively related to increasing body mass (Fig. 4a). These findings could jointly indicate that larger mammals that are less endangered could be attracting more research attention than smaller mammals that are severely endangered. However, since taxa with larger mammals, such as the big cats and African megafauna, are typically considered more charismatic [8,42] and have higher proportions of threatened species than that of smaller ones [9]. Hence, larger mammals may receive more research interest than smaller mammals, regardless of whether or not they are threatened (Fig. 3b). We found that taxa with smaller mammals in the IUCN Red List categories 'Endangered' and 'Critically Endangered' were likely to have slightly lower species h-indices. This indicates a lack of research focus on smaller species, especially those endangered, possibly because they are rarer in the wild and comparatively harder to research."

Lines 263-267: Is this comparison a fair one given that the disciplines and funding that underpin research on the tree-kangaroo and sheep/cattle are very different?

Reply 21

Since our original question is quite broad, with a focus on all species of mammals, this comparison is not necessarily unfair. However, if the study was more conservation-focused, then this would not be a good comparison. Here, we wanted to show that a species' uses, location of habitat, and funding availability could all affect their research interest (please also see our Reply 7)

Figures

Figure 3: use of symbol size seems odd for a categorical variable - could the symbol and colours be swapped for the other variable? It might be clearer

Reply 22

Thank you for the suggestion. We have replaced the previous figure with the one shown below. This new figure separates the LC species (a) from the ones in threatened classes (b), with a new figure caption.

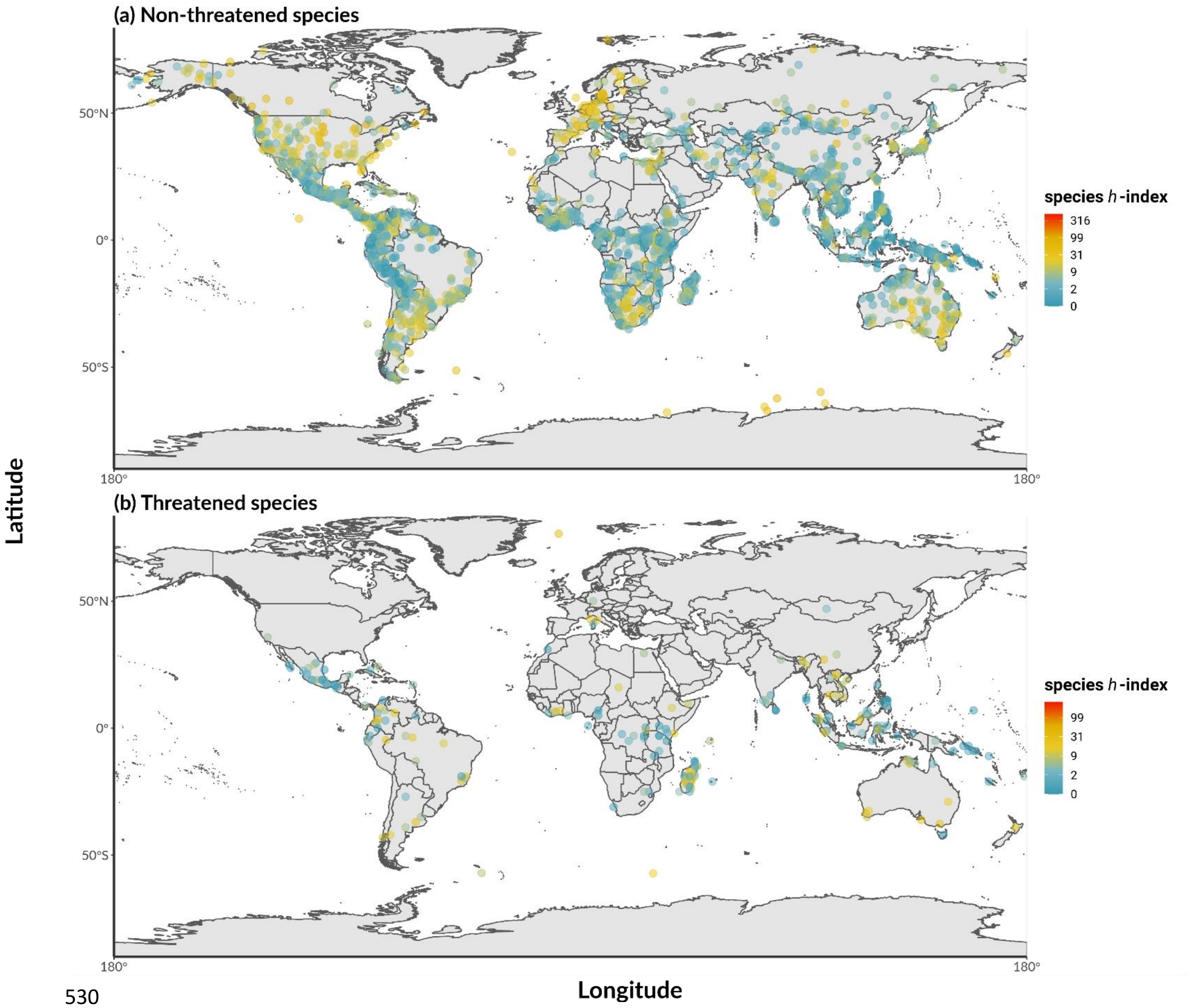

FIGURE 3 Centroids of global distributions of 4,435 mammalian species. (a) The distribution of non-threatened species listed as 'Least Concern'. (b) The distribution of threatened species listed as 'Vulnerable', 'Endangered', 'Critically Endangered', and 'Extinct in the Wild'. The species' corresponding *h*-index values are illustrated by dot colour.

Co-reviewers: Charlotte Benham, Hannah Puleston, Kate Scott-Gatty, Louise McRae
