# Supplementary material for: Quantifying research interests in 7,521 mammalian species with h-index: a case study
Source: Gigascience. 2022 Aug 13;11:giac074. doi: 10.1093/gigascience/giac074 (PMC9375528; doi:10.1093/gigascience/giac074)
Supplement: giac074_GIGA-D-21-00396_Revision_2 [file giac074_giga-d-21-00396_revision_2.pdf]

GigaScience

Quantifying research interests in 7,521 mammalian species with h-index: a case study

--Manuscript Draft--

**Manuscript Number:**	GIGA-D-21-00396R3
**Full Title:**	Quantifying research interests in 7,521 mammalian species with h-index: a case study
**Article Type:**	Research
**Funding Information:**	
**Abstract:**	Background<br>Taxonomic bias is a known issue within the field of biology, causing scientific knowledge to be unevenly distributed across species. However, a systematic quantification of the research interest that the scientific community has allocated to individual species remains a big data problem. Scalable approaches are needed to integrate biodiversity datasets and bibliometric methods across large numbers of species. The outputs of these analyses are important for identifying understudied species and directing future research to fill these gaps.<br>Findings<br>In this study, we used the species h -index to quantity the research interest in 7,521 species of mammals. We tested factors potentially driving species h -index, by using a Bayesian phylogenetic generalised linear mixed model (GLMM). We found that a third of the mammals had a species h -index of zero, while a select few had inflated research interest. Further, mammals with higher species h -index had larger body masses, were found in temperate latitudes, had more humans uses, including domestication, and were in lower risk IUCN Red List categories. These results surprisingly suggested that critically endangered mammals are understudied. A higher interest in domesticated species suggested that human use rather than conservation drives mammalian scientific literature.<br>Conclusion<br>Our study has demonstrated a scalable workflow and systematically identified understudied species of mammals, as well as identified the likely drivers of this taxonomic bias in the literature. This case study can become a benchmark for future research that asks similar biological and meta-research questions for other taxa.
**Corresponding Author:**	Jessica Tam<br>University of New South Wales - Kensington Campus: University of New South Wales<br>Kensington, New South Wales AUSTRALIA
**Corresponding Author Secondary Information:**	
**Corresponding Author's Institution:**	University of New South Wales - Kensington Campus: University of New South Wales
**Corresponding Author's Secondary Institution:**	
**First Author:**	Jessica Tam
**First Author Secondary Information:**	
**Order of Authors:**	Jessica Tam
	Malgorzata Lagisz
	Will Cornwell
	Shinichi Nakagawa
**Order of Authors Secondary Information:**	
**Response to Reviewers:**	Attached are the word documents of the main text and supplementary material. I have now properly cited the databases required.
**Additional Information:**	
**Question**	**Response**

Powered by Editorial Manager® and ProduXion Manager® from Aries Systems Corporation

Are you submitting this manuscript to a special series or article collection?	No
**Experimental design and statistics**<br><br>Full details of the experimental design and statistical methods used should be given in the Methods section, as detailed in our Minimum Standards Reporting Checklist. Information essential to interpreting the data presented should be made available in the figure legends.<br><br>Have you included all the information requested in your manuscript?	Yes
**Resources**<br><br>A description of all resources used, including antibodies, cell lines, animals and software tools, with enough information to allow them to be uniquely identified, should be included in the Methods section. Authors are strongly encouraged to cite Research Resource Identifiers (RRIDs) for antibodies, model organisms and tools, where possible.<br><br>Have you included the information requested as detailed in our Minimum Standards Reporting Checklist?	Yes
**Availability of data and materials**<br><br>All datasets and code on which the conclusions of the paper rely must be either included in your submission or deposited in publicly available repositories (where available and ethically appropriate), referencing such data using a unique identifier in the references and in the "Availability of Data and Materials" section of your manuscript.<br><br>Have you have met the above requirement as detailed in our Minimum	Yes

Standards Reporting Checklist?	

Powered by Editorial Manager® and ProduXion Manager® from Aries Systems Corporation

Manuscript
Click here to access/download;Manuscript;specieshindex_forproduction.docx

Quantifying research interests in 7,521 mammalian species with *h*-index: a case study

Jessica Tam[1]*, Malgorzata Lagisz[1%], Will Cornwell[1%] and Shinichi Nakagawa[1%]

Add affiliation

1 Evolution & Ecology Research Centre and School of Biological, Earth and Environmental Sciences, University of New South Wales, Sydney, Australia

*Correspondence: jessicatin-ying.tam@unsw.edu.au

%: these authors shared supervision responsibilities equally

ORCID IDs:

Jessica Tam [0000-0003-3655-1974]; Malgorzata Lagisz [0000-0002-3993-6127]; Will Cornwell [0000-0003-4080-4073]; Shinichi Nakagawa [0000-0002-7765-5182]

Abstract

Background

Taxonomic bias is a known issue within the field of biology, causing scientific knowledge to be unevenly distributed across species. However, a systematic quantification of the research interest that the scientific community has allocated to individual species remains a big data problem. Scalable approaches are needed to integrate biodiversity datasets and bibliometric

methods across large numbers of species. The outputs of these analyses are important for identifying understudied species and directing future research to fill these gaps.

Findings

In this study, we used the species *h*-index to quantity the research interest in 7,521 species of mammals. We tested factors potentially driving species *h*-index, by using a Bayesian phylogenetic generalised linear mixed model (GLMM). We found that a third of the mammals had a species *h*-index of zero, while a select few had inflated research interest. Further, mammals with higher species *h*-index had larger body masses, were found in temperate latitudes, had their humans uses documented, including domestication, and were in lower risk IUCN Red List categories. These results surprisingly suggested that critically endangered mammals are understudied. A higher interest in domesticated species suggested that human use is a major driver and focus in mammalian scientific literature.

Conclusion

Our study has demonstrated a scalable workflow and systematically identified understudied species of mammals, as well as identified the likely drivers of this taxonomic bias in the literature. This case study can become a benchmark for future research that asks similar biological and meta-research questions for other taxa.

KEYWORDS: bibliometrics, research bias, meta-research, scientific mapping, research on research, topic modeling

Introduction

Effective conservation of the earth's amazing biodiversity requires sound knowledge of species' biology and ecology, with the addition of adequate communication from scientists [1]. However, such knowledge is often not only missing [2], but also biased. Some species receive disproportionally more research interest while others very little, reflected in scientific publications – known as taxonomic bias [3]. Although taxonomic bias in the scientific literature is prevalent [4,5], there has been little effort to rectify the problem. Even worse, this problem seemed to have become more extreme in the last few decades [6,7]. To work towards reducing the gaps of knowledge in the literature, one first need to understand what is causing such inequality in research interest among species.

Many potential drivers exist for taxonomic bias. For instance, there is a human preference to study and conserve iconic or 'charismatic' taxa, which are usually large mammals such as the African bush elephant (*Loxodonta africana*) and black rhinoceros (*Diceros bicornis*) [8]. Indeed, large mammalian vertebrates are over-represented in the conservation literature [9,10]. Of relevance, the anthropomorphic stimuli hypothesis posits that humans are attracted to species that are more phylogenetically related to us [11]. Such human tendencies likely explain the inflated research effort towards vertebrate taxa [5]. This hypothesis is also related to the reason why we have much (bio)medical research, using rodent model systems such as rats (*Rattus norvegicus* and mice *Mus musculus*), because of our shared physiological traits [12]. Studying species closer to scientists' proximity [5,13], where the animals live in accessible locations, and for economic reasons, such as agriculture and aquaculture research, can also exacerbate taxonomic bias in the literature. Consequently, these drivers have over time created strong unevenness in the taxonomic distribution of scientific knowledge.

Researchers have investigated such taxonomic bias in the academic literature, but these studies appeared to have two main shortcomings. First, because of the previous difficulties constructing scalable workflows, the coverage of these studies is often not comprehensive. While several studies have quantified species-level bias among plants [14], mammals [15–18], birds [19], fish [20], and amphibians [21] respectively, their sample sizes remain no more than a few hundred species, encompassing only small portions of species in a given taxonomic group. Until now, only two studies have evaluated species-level taxonomic bias for the thousands of species and across multiple clades [4,22]. However, these studies focused solely on species included on the International Union for Conservation of Nature (IUCN) Red List, therefore, potentially failing to provide more comprehensive and holistic understanding of the drivers of taxonomic bias in research.

Second, there are currently no standardized methods to quantify taxonomic bias at the level of individual species. Publication count is one of the most commonly used proxy to gauge taxonomic bias [4,5,7,15,18,20–24]. However, while the total number of publications could capture the total research effort on a given species, it does not capture research interest per se (i.e., how much attention from research community these publications received). A logical alternative would be to use citation count [25], as it captures the total research interest. Nonetheless, high impact papers can easily inflate this number [26] and give a false impression that a species is receiving more interest than in reality. Hirsch's *h*-index [26] kills two birds with one stone by taking into account both the number of publications and number of citations. So far, there exist only a handful of studies that have adapted the 'species' *h*-index' for measuring and comparing research interests among different species [14,16,17,19,27].

This study seeks to quantify the research interest in mammals, using the species *h*-index [14,16,17,19]. We introduce a workflow demonstrating how to obtain species *h*-index for any species and how to ask relevant meta-science as well as biological questions on research interest. As a case study, we choose the class Mammalia, which consists of over 7,500, species, since they are one of the most well-studied taxonomic groups, with extensive data readily available. Then, we test how our surrogate for research interest, species *h*-index, could be related to the following six potential drivers: 1) body size, 2) location of natural habitat, 3) phylogenetic relatedness, 4) human uses and domestication, 5) (IUCN Red List status, and 6) general interest (encompassing drivers 1 - 5, quantified via Google Trends; see below). We outline our hypothesis and rationale for each potential driver in Table 1.

Methods

Data collection and processing

For much of data collection and cleaning as well as all statistical analyses (see below), we used the R language version 4.0.2 [28] in the RStudio environment version 1.3.1093 [29]. The source code of this article can be found on Github [30] and the data can be found on Zenodo [31,32].

We first collected a list of mammalian species from the Open Tree of Life (OTL) database [33] using the R package *rotl* version 3.0.12 [34] to create a complete mammalian species list. We removed sub-species from the list and only kept species with binomial names, resulting in 6,952 species. Next, we obtained lists characteristics of mammalian species represented as 7 statistical surrogate of the 6 potential drivers of research interest (Table 1): 1) body mass (n =

5,400; in grams, log_{10} transformed) 2) median latitude of species range (n = 4,721; obtained from centroids of all occurrence records from GBIF), 3) phylogenetic trees with branch lengths (n = 5,911 [35]), 4) IUCN Red List human use categories (n = 1,472; a binary categorial variable where a species was categorized into at least one of 19 human uses), 5) Wikipedia list of domesticated species (n = 159; a 3-level categorical variable: domesticated, partially domesticated & wild), 6) IUCN Red List status (n = 5,584; an ordinary variable with 6 levels: 'Least Concern, 'Near Threatened', 'Vulnerable', 'Endangered', 'Critically Endangered', and 'Extinct in the Wild' excluding 'Extinct' and 'Data Deficient'; there was some discrepancy with the IUCN Red List statuses when filling in missing data in this category, we suspect this is an issue caused by a mismatch between the data on the IUCN Red List website and their API through the *rredlist* R package, we suggest using data from the website as the package might not be regularly maintained), and 7) Google Trends index (n = 7,521; see Appendix Fig. S1 for a summary of the data completeness and data processing details and see the Supplementary information). Synonym matching was performed automatically with *rotl::tnrs_match_names()*, before combining the categories and the list from OTL to form 1 dataset. Duplicated names were removed using the functions *unique()* and *duplicate()*. A total of 7,521 unique species remained on the final species list. We obtained the Google Trends index after finalising the list of species names.

Notably, we added higher taxonomic clades to condense the 30 orders to 5 major clades according to molecular tree reconstructions [35,36]. These five high-lever taxa are: 1) Afrotheria representing an African lineage, including sea cows and elephants, 2) Xenarthra representing an American lineage that includes sloths and armadillos, 3) Euarchontoglires representing widely distributed species such as rodents and primates, 4) Laurasiatheria

representing species such as whales, carnivores, and bats, and finally 5) Marsupials & Monotremes representing the non-eutherian mammals. We used these higher taxonomic groupings in visualizations of the results.

Data sources and species *h*-index

We extracted the bibliometric records from Scopus (data collection on 28 April 2021) and calculated the *h*-index of individual mammal species with the R package *specieshindex* [37]. The package connects to the Scopus, Web of Science, and Bielefeld Academic Search Engine (BASE) literature databases. Using either binomial or genus names, the package can count the number of relevant bibliometric records for each species or genus on each database and extract them for local processing and analysis. Bibliometric information that can be extracted include citation count, publication date, authors, and more. *specieshindex* can then calculate the species *h*-index of individual species applying Hirsch's *h*-index [26]. The *h*-index is defined as the largest number of publications (n) cited a minimum of the same number (n) of times (Appendix Fig. S2). The *h*-index in this scenario quantifies the research interest each individual species has received. The package has also implemented the calculation of other indices, such as the *m*-index, and *h5* index, and plotting functionality.

We used binomial names in Scopus database searches because of the ambiguity and lack of common names for uncommon species. We tackled the issue of species name synonyms by using the Boolean term 'OR' between each synonymous binomial name (collected from Open Tree of Life) in the search string. Articles containing binomial names of mammals in their title, abstract, or keywords, were extracted. Since the distribution of *h*-index was right-skewed with

more species having a lower species h-index, we applied the formula $log_{10}(h+1)$ for visualization purposes, but we used the original count data for modeling (see below).

Imputing missing data

The coverage of data is lower for some predictors (Appendix Fig. S1) as a result of synonym matching and cleaning. Since some data was missing for body mass, latitude, and IUCN Red List status (Appendix Fig. S1), we imputed missing values for 5,497 species that were include in the model, to match the shorter length of the phylogenetic tree. We used the multiple imputation approach implemented in the R package *mice* [38]. Multiple imputation creates multiple sets of imputed values before aggregating them to create a single set of data [39]. This is preferred over deletions of data records with missing values, as the latter can result in lowered statistical power and biases in the parameter estimates [40]. We used binomial name, *h*-index, human use, domestication, and Google Trends index to impute 3 variables with missing values (body mass, latitude, and IUCN Red List status), creating 10 complete datasets for statistical analyses.

Statistical analysis and phylogenetic 'heritability'

We ran two Bayesian phylogenetically controlled Poisson mixed models with the log link function and the additive dispersion term [41], implemented in the R package *MCMCglmm* version 2.33 [42] and ran using the computation cluster Katana at UNSW Sydney [43]. The first model followed the predictions stated in the hypotheses (Table 1), and used the dataset with the sample size of 5,497 species, and 50 identical phylogenetic trees with branch lengths chosen randomly from Upham et al. [35]. Fifty trees were selected since it is the minimum number of trees needed to account for uncertainties in phylogenetic data [44]. The second

model was the same as the first one but with only 5,343 species after removing domesticated and semi-domesticated species (i.e., one less predictor or fixed effect than the first two models; see formulae below). We added this model because (semi-)domesticated species are likely to have inflated species *h*-index values which may not be comparable to those of wild species.

We ran 130,000 iterations for the chain with 30,000 burn-ins, drawing 1,000 samples from the imputed data in each iteration, and using a non-informative prior for both fixed and random effects. To obtain more accurate precision of model estimates, we repeated the same model for the 10 imputed datasets and 50 phylogenetic trees, resulting in a total of 500 model runs for each model respectively. The last 100 of the total 1,000 samples of each model were extracted for the calculation of the model results.

In the first model, we used the following predictor variables: body mass value on $\log_{10}$ scale (continuous), the absolute value of median latitude (continuous; converted to absolute value for linear distribution), human use (binomial), domestication (ordinal), IUCN Red List status (ordinal), and Google Trends index on ($\log_{10}$ + 1) scale (binomial) to model the outcome variable species *h*-index (count), as in the following formula:

$$h \sim log_{10}(Body\ mass) + |Latitude| + Human\ use + Domestication$$

$$+ IUCN\ Red\ List\ status + (IUCN\ Red\ List\ status)^2 + log_{10}(Google\ Trends + 1).$$

The second model in the following formula (without Domestication):

$$h \sim log_{10}(Body\ mass) + |Latitude| + Human\ use$$

$$+ IUCN\ Red\ List\ status + (IUCN\ Red\ List\ status)^2 + log_{10}(Google\ Trends + 1).$$

During our preliminary analysis, we checked for variance inflation factor (VIF) to make sure that the regressors were not correlated to each other. The VIF values ranged between 1.0 – 1.7 (Appendix Table S3). Low VIF values meant that the predictor variables are not co-linear and will not lead to inflated correlations.

We estimated phylogenetic heritability (H^2; [41]) to check for phylogenetic correlations among species, which is equivalent to Pagel's *lambda* (λ). Values of H^2 fall between 0 and 1. The output of the Bayesian model provided the values needed for H^2 calculation using the following formula, from Nakagawa et al. [45]:

$$H^2 = \frac{var(species)}{var(species) + var(overdispersion) + ln\left(1 + \frac{1}{mean(h)}\right)}$$

where *var*(*species*) and *var*(*overdispersion*) are the variance components for phylogenetic effects and the additive overdispersion term, which is equivalent to the residual term in a normal regression and *mean*(*h*) represents the average *h*-index values.

Results

General trends of species' *h*-index across taxa

We calculated the species *h*-index for 7,521 species of mammals in total. A species *h*-index of 0 was common in mammals with 32.26% (n = 2,426; Fig. S4) failing to have even one paper cited one time (Fig. 1). On the other hand, mammals with a species *h*-index of 100 and higher only included 34 species from across 6 orders (Fig. 1a). The median and mean of the h-index for all the species were h_{median} = 2 and h_{mean} = 7.08 respectively. After removing domesticated (and

semi-domesticated) species from the dataset (remaining n = 7,360), mammals with a *h*-index of 100 and higher only included 17 species from Carnivora and Primates (7 and 10 species respectively; Fig. 1b). The median and mean of the species *h*-index without the domesticated mammals are h_{median} = 2 and h_{mean} = 6.16 respectively.

There were also pronounced shifts in research interest through time. Publications in the early 1940s were largely on the orders Hyracoidea (hyraxes), Proboscidea (elephants), Soricomorpha (dissolved paraphyletic taxa of shrews – combined with Erinaceidae to form Eulipotyphla), and Didelphimorphia (opossums) (Fig. 2b). Upon skimming the titles of some articles (around 10 titles), we noted that early publications in these species appeared to be mostly comparative anatomy studies. In the 1950's, the mammalian literature took on its modern structure, with research focused largely on 6 orders (Fig. 2a) – rodents (Rodentia, 1950-2021 mean = 30.94% of the yearly article count), Primates (1950-2021 mean = 13.98%), bats (Chiroptera, 1950-2021 mean = 11.16%), carnivores (Carnivora, 1950-2021 mean = 11.61%), pigs, sheep, cattle and other even toed ungulates (Artiodactyla, 1950-2021 mean = 11.83%), and whales and dolphins (Cetacea, 1950-2021 mean = 3.15%). Higher species *h*-index was generally associated with larger body sizes (Fig. 4a), intermediate latitudes (Fig. 3, Fig. 4b), more human uses (Fig. 4c) and domestication (Fig. 4d), lower extinction risk (Fig. 4e), and higher general interest (Fig. 4f).

Statistical predictors of species' *h*-index and phylogenetic signal

We included 5,497 species of mammals in the first (Table 2) and 5,343 species (after excluding domesticated animals) in the second (Table 3) Bayesian generalized linear mixed model (BGLMM). In both models, body size positively and significantly predicted species *h*-index (Tables 2 & 3; Fig. 4a). While mammalian diversity was highest in the tropics, species found

here had significantly lower species *h*-indices compared to those in the temperate regions and near the poles, which was again supported in the models (Tables 2 & 3; Fig. 3; Fig. 4b). Although most mammals had a Google Trends index of 0, species *h*-index significantly increased with the Google Trends index in all models (Tables 2 & 3; Fig. 4f). There was a u-shaped distribution across the IUCN Red List statuses (Fig. 4e) with a statistically significant quadratic effect in both models (i.e., with and without domesticated animals; Tables 2 & 3; see also Appendix Fig. S5 for IUCN Red List statuses not included in the model). These models also showed a statistically significant linear decline of species *h*-index with increasing extinction risk (IUCN Red List status). Further, species *h*-index significantly increased with human use in both models (Tables 2 & 3; Fig. 4c; see Appendix Fig. S6 for all human use categories). The first two models showed that domestication status was a significant positive predictor of species *h*-index (Tables 2 & 3; Fig. 4d). Finally, phylogenetic signal was present in species *h*-index in both models (Tables 2 & 3; see Appendix Fig. S7 for the phylogenetic tree).

Discussion

Scientific research is not spread evenly across mammal species: we found strong bias in 'research interest' in the literature, quantified by species *h*-index. A small group of species (n = 34 with all species and n = 17 without domestication species) had a species *h*-index above 100, while one-third of the species (n = 2,426 with or without domestication species) received no scientific interest at all (h = 0) (Fig. 1). The modern mammalian literature was dominated by the orders Rodentia, Primates, Carnivora, Artiodactyla, Chiroptera, and Cetacea (Fig. 2), which resulted in a high value of phylogenetic heritability in the model (H^2 = 64%; Table 2). Overall, our analyses confirmed our predictions (Table 1). The bias towards a few orders also appeared

in species with high species *h*-indices (Fig. 1) and these commonly found in the high latitudes (Fig. 3). Mammals with high species *h*-indices were more likely to be large, less endangered, and have their utility documented (Fig. 4). These 'research superstars' include farmed animals, pets, and laboratory small mammals, as expected.

Low research interest in endangered small mammals

The relationship between IUCN Red List status and species *h*-index (Fig. 4d) resembled a u-shaped distribution and this trend was statistically significant in both models with and without domesticated animals (Table 3). Also, we found a significant decline (a significant linear effect) in research interest (species *h*-index) with conservation status (i.e., for more endangered mammals). Collectively, these quadratic and linear effects indicate that some endangered species may enjoy higher species *h*-indices, such as the lion (*Panthera leo*) and the orangutan (*Pongo pygmaeus*) (Fig. 1).

We also found that species *h*-index is positively related to increasing body mass (Fig. 4a). These findings could jointly indicate that larger mammals that are less endangered could be attracting more research attention than smaller mammals that are severely endangered. Since taxa with larger mammals, such as the big cats and African megafauna, are typically considered more charismatic [8,46], larger mammals may receive more research interest than smaller mammals, regardless of whether or not they are threatened (Fig. 3b). We found that taxa with smaller mammals in the IUCN Red List categories 'Endangered' and 'Critically Endangered' were likely to have slightly lower species *h*-indices. This indicates a lack of research focus on smaller species, especially those endangered, possibly because they are rarer in the wild and comparatively harder to research.

High research interest with domestication and phylogenetic relatedness

Domesticated species were among the top ranks of mammals with the highest species *h*-indices (Fig. 1a, Fig. 4d). Mammals with human uses documented also had higher species *h*-indices than species with no documented human uses (Fig. 4c). However, some species lack documentation on human their uses because the data on human uses are patchy and not reliable for locally used species. The strong focus on pets and livestock animals can be explained by their global proximity to humans as well as our needs and preferences. Among all mammals on earth, wild mammals only make up 4% of the total mammalian biomass, while humans and livestock combine to form the other 96% [50], and this corresponds with their widespread occurrence due to the globalization of a small number of animal husbandry systems [51]. Our need to make our animal use more efficient has clearly driven high volumes of research on these animals.

For example, the literature on cattle or sheep can have contributions and interested readers from all over the world. The broad readership creates academic rewards for researchers and thus positive feedback towards an ever-expanding literature on these animals. In contrast, the research on the grizzled tree-kangaroo, a vulnerable wild species, can only be done on New Guinea and surrounding islands, severely limiting both the pool of potential researchers and potential readers of that research. Thus, not only is it financially and logistically difficult to research grizzled tree-kangaroos, but the readership and academic rewards for doing research in species without any direct human uses are very limited.

We also found phylogenetic signals in species *h*-indices (Tables 2 & 3), meaning some taxonomic groups usually had higher *h*-indices than others (Fig. 1). Many livestock animals are phylogenetically related, such as the pig (Sus scrofa), the sheep (*Ovis Aries*), and the cow (*Bos taurus*) (Fig 1a), all of which belong to the order Artiodactyla. Furthermore, several primates had relatively high species *h*-indices compared to those from other taxa. Indeed, when we removed the domesticated species, around 65% of the species with *h* = 100 or more were primates (Fig 1b). This finding strongly supports the anthropomorphic stimuli hypothesis [11], where humans tend to be more attracted to species that are phylogenetically similar to us.

Geographical bias towards species in developed countries

We found that mammals with higher species *h*-indices were congregated in clusters centered at the temperate latitudes (Fig. 3, Fig. 4b). Some of these locations – in the USA, Europe, and Australia – are regions with high gross domestic product values, GDP [52], characteristic for developed countries. Not only are scientists in developed countries able to carry out more research activities with better funding, but they have better access to the infrastructure, such as laboratories, transport, and equipment. Higher education is also better implemented in these regions, which is largely lagging in developing countries [53,54]. Developing countries not often require even more research funding to compensate for the scarcity of resources [55]. Since developed countries dominate global publication output [56], the geographical biases revealed in our analyses therefore reflect the research interests of scientists in wealthier countries.

Academic preferences towards certain mammal species also suggest that convenience is often prioritized. This trend is evident in Fig. 3, where species near the tropics had much lower

species *h*-indices than those in temperate zones, regardless of their extinction risk. Such preference towards species in the temperate zone is not unique to Mammalia. Scientific literature on species across all taxa, both vertebrates and invertebrates, is biased towards the temperate environment [57]. This is alarming given that 55% of species in the tropics are at risk of local extinctions from climate change, which is higher than that of temperate species, at 39% [58]. At the same time, tropical regions are biodiversity hotspots because of their high species richness [59]. However, considering that funding in science is often limited, projects that yield the best results with the lowest cost may receive more resources and support.

Potential limitations and future perspectives

This study has four major limitations. Firstly, the data sources included varying lists of mammals with available information, resulting in missing values in some of our predictors (body mass, latitude, and IUCN Red List status) (Appendix Fig. S1). Although this issue was mitigated by imputing values, the results of our study would be more reliable if complete data was available. Further, some species may have been dropped from the analyses as their binomial names were spelled differently from the current consensus name. Although we attempted to incorporate synonyms and remove species that went extinct during the prehistorical and historical times, some synonyms with different spellings and extinct species might still be present in the dataset. This can potentially explain why the sample size of this study is 7,521 species of mammals, much higher than Burgin et al.'s [60] resolved list with only 6,495 species. The issue of unresolved taxonomy is likely going to affect similar studies that attempt to gather high volumes of data for multiple species from other taxa [61].

Secondly, we used the *h*-index [26] as a measurement of research interest since it takes into account both number of publications and numbers of citations. However, there are other similar indices that can be used to quantify research output and influence, including the *h5* index, *m*-index, and *i10* index. The *h5* index is the *h*-index of publications that were published in the past 5 years [62]. The *m*-index is the *h*-index divided by the number of years since the first publication [26], which directly scales for time (Appendix Fig. S8). Indirectly, the *h*-index can also indicate the time dimension, assuming that more time associates with more publications and more citations. The *i10* index is the total number of articles with 10 or more citations; it is currently used by Google Scholar [63]. Future studies can compare these indices and investigate how they differ with *specieshindex* R package, which can calculate these other indices.

Third, we used species *h*-index here to characterize the distribution of research interest across mammalian species. More research interest does not inform us on the kinds of research that has been done for a given species. Text mining could be used on full-text publications to single out studies with a given topic (e.g., conservation, behaviour, ecology or biomedical use) in future studies, although such endeavor would require access to full-texts.

Finally, although a proxy for general interest in species, presence in Google searches, was a strong and statistically significant predictor of species *h*-index (Fig. 4c, Appendix Table 2), members of the public, in general, are unlikely to use binomial names of species, which we used in this study. We decided against the use of common names for our analyses as many species have multiple common names and many common species names are often used the name of products or companies, and our searches would result in very messy data. Therefore, we require a better proxy for quantifying public interest in different species.

Conclusion

This study has quantified species *h*-index for all available mammalian species as a case study and asked meta-scientific and biological questions. We have elucidated the current patchiness and biases in the mammalian research landscape using potential drivers of such biases that have been hypothesized before, but perhaps at the largest and finest scale than previously done. More importantly, we have demonstrated potential of addressing meta-research and biological questions by combining available online datasets and species *h*-indices calculated from a bibliometric database. Therefore, future studies can ask a rich set of similar and extended questions to quantify the research landscape of any taxa.

Acknowledgements

We are grateful for the comments from Prof. Ian Suthers and A/Prof. Tracy Ainsworth from UNSW Sydney. This research includes computations using the computational cluster Katana supported by Research Technology Services at UNSW Sydney.

Data availability

The source code of this article can be found on Github [30] and the data can be found on Zenodo [31,32]. Additional information is available at the end of the article as supplementary material. An archival copy of the datasets and GitHub Repository is available via the GigaScience database, GigaDB [64].

References

1. Rudd MA. How Research-Prioritization Exercises Affect Conservation Policy. Conserv Biol. 2011; doi: 10.1111/j.1523-1739.2011.01712.x.

2. Gerlach J, Samways MJ, Hochkirch A, Seddon M, Cardoso P, Clausnitzer V, et al.. Prioritizing non-marine invertebrate taxa for Red Listing. J Insect Conserv. 2014; doi: 10.1007/s10841-014-9660-6.

3. Bonnet X, Shine R, Lourdais O. Taxonomic chauvinism. Trends Ecol Evol. 2002; doi: 10.1016/S0169-5347(01)02381-3.

4. Donaldson MR, Burnett NJ, Braun DC, Suski CD, Hinch SG, Cooke SJ, et al.. Taxonomic bias and international biodiversity conservation research. FACETS. 2017; doi: 10.1139/facets-2016-0011.

5. Titley MA, Snaddon JL, Turner EC. Scientific research on animal biodiversity is systematically biased towards vertebrates and temperate regions. PLOS ONE. 2017; doi: 10.1371/journal.pone.0189577.

6. Troudet J, Grandcolas P, Blin A, Vignes-Lebbe R, Legendre F. Taxonomic bias in biodiversity data and societal preferences. Sci Rep. 2017; doi: 10.1038/s41598-017-09084-6.

7. Rosenthal MF, Gertler M, Hamilton AD, Prasad S, Andrade MCB. Taxonomic bias in animal behaviour publications. Anim Behav. 2017; doi: 10.1016/j.anbehav.2017.02.017.

8. Berti E, Monsarrat S, Munk M, Jarvie S, Svenning J-C. Body size is a good proxy for vertebrate charisma. Biol Conserv. 2020; doi: 10.1016/j.biocon.2020.108790.

9. Ripple WJ, Wolf C, Newsome TM, Hoffmann M, Wirsing AJ, McCauley DJ. Extinction risk is most acute for the world's largest and smallest vertebrates. Proc Natl Acad Sci. 2017; doi: 10.1073/pnas.1702078114.

10. Seddon PJ, Soorae PS, Launay F. Taxonomic bias in reintroduction projects. Anim Conserv. 2005; doi: 10.1017/S1367943004001799.

11. Miralles A, Raymond M, Lecointre G. Empathy and compassion toward other species decrease with evolutionary divergence time. Sci Rep. 2019; doi: 10.1038/s41598-019-56006-9.

12. Bryda EC. The Mighty Mouse: The Impact of Rodents on Advances in Biomedical Research. Mo Med. 2013; 110:207–112013.

13. Di Marco M, Chapman S, Althor G, Kearney S, Besancon C, Butt N, et al.. Changing trends and persisting biases in three decades of conservation science. Glob Ecol Conserv. 2017; doi: 10.1016/j.gecco.2017.01.008.

14. Adamo M, Chialva M, Calevo J, Bertoni F, Dixon K, Mammola S. Plant scientists' research attention is skewed towards colourful, conspicuous and broadly distributed flowers. Nat Plants. 2021; doi: 10.1038/s41477-021-00912-2.

15. dos Santos JW, Correia RA, Malhado ACM, Campos-Silva JV, Teles D, Jepson P, et al.. Drivers of taxonomic bias in conservation research: a global analysis of terrestrial mammals. Anim Conserv. 2020; doi: 10.1111/acv.12586.

16. Fleming PA, Bateman PW. The good, the bad, and the ugly: which Australian terrestrial mammal species attract most research? Mammal Rev. 2016; doi: 10.1111/mam.12066.

17. Robertson PA, McKenzie AJ. The scientific profiles of terrestrial mammals in Great Britain as measured by publication metrics: Publication metrics of mammals in Great Britain. Mammal Rev. 2015; doi: 10.1111/mam.12038.

18. Tensen L. Biases in wildlife and conservation research, using felids and canids as a case study. Glob Ecol Conserv. 2018; doi: 10.1016/j.gecco.2018.e00423.

19. McKenzie AJ, Robertson PA. Which Species Are We Researching and Why? A Case Study of the Ecology of British Breeding Birds. PLOS ONE. 2015; doi: 10.1371/journal.pone.0131004.

20. Ducatez S. Which sharks attract research? Analyses of the distribution of research effort in sharks reveal significant non-random knowledge biases. Rev Fish Biol Fish. 2019; doi: 10.1007/s11160-019-09556-0.

21. Schiesari L, Grillitsch B, Grillitsch H. Biogeographic Biases in Research and Their Consequences for Linking Amphibian Declines to Pollution. Conserv Biol. 2007; doi: 10.1111/j.1523-1739.2006.00616.x.

22. Trimble MJ, van Aarde RJ. Species Inequality in Scientific Study. Conserv Biol. 2010; doi: 10.1111/j.1523-1739.2010.01453.x.

23. da Silva AF, Malhado ACM, Correia RA, Ladle RJ, Vital MVC, Mott T. Taxonomic bias in amphibian research: Are researchers responding to conservation need? J Nat Conserv. 2020; doi: 10.1016/j.jnc.2020.125829.

24. Watkins HV, Yan HF, Dunic JC, Côté IM. Research biases create overrepresented "poster children" of marine invasion ecology. Conserv Lett. 2021; doi: https://doi.org/10.1111/conl.12802.

25. Wang Z, Zeng J, Meng W, Lohman DJ, Pierce NE. Out of sight, out of mind: public and research interest in insects is negatively correlated with their conservation status. Insect Conserv Divers. 2021; doi: 10.1111/icad.12499.

26. Hirsch JE. An index to quantify an individual's scientific research output. Proc Natl Acad Sci. 2005; doi: 10.1073/pnas.0507655102.

27. Cox R, McIntyre KM, Sanchez J, Setzkorn C, Baylis M, Revie CW. Comparison of the h-Index Scores Among Pathogens Identified as Emerging Hazards in North America. Transbound Emerg Dis. 2016; doi: 10.1111/tbed.12221.

28. R Core Team (2020). R (Version 4.0.2) https://www.R-project.org

29. RStudio Development Team (2020). RStudio (Version 1.3.1093) https://rstudio.com

30. Tam J. jessicatytam/biases_in_mammalian_research: GigaScience publication code & data. Zenodo. 2022. https://doi.org/10.5281/zenodo.6672954.

31. Tam J. Quantifying research interests in 7,521 mammalian species with h-index: a case study (scopus output). Zenodo. 2021. https://doi.org/10.5281/zenodo.5711932.

32. Tam J. Quantifying research interests in 7,521 mammalian species with h-index: a case study (model output). Zenodo. 2022. https://doi.org/10.5281/zenodo.6644032.

33. McTavish EJ, Hinchliff CE, Allman JF, Brown JW, Cranston KA, Holder MT, et al.. Phylesystem: a git-based data store for community-curated phylogenetic estimates. Bioinformatics. 2015; doi: 10.1093/bioinformatics/btv276.

34. Michonneau F, Brown JW, Winter DJ. rotl: an R package to interact with the Open Tree of Life data. Fitzjohn R, editor. Methods Ecol Evol. 2016; doi: 10.1111/2041-210X.12593.

35. Upham NS, Esselstyn JA, Jetz W. Inferring the mammal tree: Species-level sets of phylogenies for questions in ecology, evolution, and conservation. PLOS Biol. 2019; doi: 10.1371/journal.pbio.3000494.

36. dos Reis M, Inoue J, Hasegawa M, Asher RJ, Donoghue PCJ, Yang Z. Phylogenomic datasets provide both precision and accuracy in estimating the timescale of placental mammal phylogeny. Proc R Soc B Biol Sci. 2012; doi: 10.1098/rspb.2012.0683.

37. Tam J (2020). specieshindex (Version 0.2.1) https:// https://github.com/jessicatytam/specieshindex

38. van Buuren S van, Groothuis-Oudshoorn K. mice: Multivariate Imputation by Chained Equations in R. J Stat Softw. 2011; doi: 10.18637/jss.v045.i03.

39. Nakagawa S. Missing data: mechanisms, methods, and messages. In: Fox GA, Negrete-Yankelevich A, Sosa VJ, editors. Ecological Statistics: Contemporary Theory Application. Oxford: Oxford University Press; 2015. p. 81–105.

40. Rubin DB. Inference and missing data. Biometrika. 1976; doi: 10.1093/biomet/63.3.581.

41. Hadfield JD, Nakagawa S. General quantitative genetic methods for comparative biology: phylogenies, taxonomies and multi-trait models for continuous and categorical characters. J Evol Biol. 2010; doi: 10.1111/j.1420-9101.2009.01915.x.

42. Hadfield JD. MCMC Methods for Multi-Response Generalized Linear Mixed Models: The MCMCglmm R Package. J Stat Softw. 2010; doi: 10.18637/jss.v033.i02.

43. PVC (Research Infrastructure) . (2010). Katana. https://doi.org/10.26190/669x-a286

44. Nakagawa S, De Villemereuil P. A General Method for Simultaneously Accounting for Phylogenetic and Species Sampling Uncertainty via Rubin's Rules in Comparative Analysis. Syst Biol. 2019; doi: 10.1093/sysbio/syy089.

45. Nakagawa S, Johnson PCD, Schielzeth H. The coefficient of determination R2 and intra-class correlation coefficient from generalized linear mixed-effects models revisited and expanded. J R Soc Interface. Royal Society; 2017; doi: 10.1098/rsif.2017.0213.

46. Albert C, Luque GM, Courchamp F. The twenty most charismatic species. PLOS ONE. 2018; doi: 10.1371/journal.pone.0199149.

47. Driscoll CA, Macdonald DW, O'Brien SJ. From wild animals to domestic pets, an evolutionary view of domestication. Proc Natl Acad Sci. 2009; doi: 10.1073/pnas.0901586106.

48. Perri AR, Feuerborn TR, Frantz LAF, Larson G, Malhi RS, Meltzer DJ, et al.. Dog domestication and the dual dispersal of people and dogs into the Americas. Proc Natl Acad Sci. 2021; doi: 10.1073/pnas.2010083118.

49. vonHoldt BM, Shuldiner E, Koch IJ, Kartzinel RY, Hogan A, Brubaker L, et al.. Structural variants in genes associated with human Williams-Beuren syndrome underlie stereotypical hypersociability in domestic dogs. Sci Adv. 2017; doi: 10.1126/sciadv.1700398.

50. Bar-On YM, Phillips R, Milo R. The biomass distribution on Earth. Proc Natl Acad Sci. 2018; doi: 10.1073/pnas.1711842115.

51. Diamond JM. Guns, germs, and steel: the fates of human societies. 1st ed. New York: W.W. Norton & Co; 1997.

52. The World Bank, World Development Indicators: GDP (current US$). https://data.worldbank.org/indicator/NY.GDP.MKTP.CD (2019). Accessed 27 Jun 2021.

53. Harris E. Building scientific capacity in developing countries. EMBO Rep. 2004; doi: 10.1038/sj.embor.7400058.

54. Vose PB, Cervellini A. Problems of scientific research in developing countries. IAEA Bull. 1983; 25:37–40.

55. van Helden P. The cost of research in developing countries. EMBO Rep. 2012; doi: 10.1038/embor.2012.43.

56. Jaffe K, Horst E ter, Gunn LH, Zambrano JD, Molina G. A network analysis of research productivity by country, discipline, and wealth. PLOS ONE. Public Library of Science; 2020; doi: 10.1371/journal.pone.0232458.

57. Culumber ZW, Anaya-Rojas JM, Booker WW, Hooks AP, Lange EC, Pluer B, et al.. Widespread Biases in Ecological and Evolutionary Studies. BioScience. 2019; doi: 10.1093/biosci/biz063.

58. Wiens JJ. Climate-Related Local Extinctions Are Already Widespread among Plant and Animal Species. PLOS Biol. 2016; doi: 10.1371/journal.pbio.2001104.

59. Ceballos G, Ehrlich PR. Global mammal distributions, biodiversity hotspots, and conservation. Proc Natl Acad Sci. 2006; doi: 10.1073/pnas.0609334103.

60. Burgin CJ, Colella JP, Kahn PL, Upham NS. How many species of mammals are there? J Mammal. 2018; doi: 10.1093/jmammal/gyx147.

61. Remsen D. The use and limits of scientific names in biological informatics. ZooKeys. 2016; doi: 10.3897/zookeys.550.9546.

62. Crotty D. Other Metrics: beyond the Impact Factor. Eur Heart J. 2017; doi: 10.1093/eurheartj/ehx446.

63. Noruzi A. Impact Factor, h-index, i10-index and i20-index of Webology. Webology. 2016; 13:1–4.

64. Tam J, Lagisz M, Cornwell W, Nakagawa S. Supporting data for “Quantifying research interests in 7,521 mammalian species with h-index: a case study.” GigaScience Database. 2022. https://doi.org/10.5524/102237.

65. Wilman H, Belmaker J, Simpson J, Rosa C de la, Rivadeneira MM, Jetz W. EltonTraits 1.0: Species-level foraging attributes of the world’s birds and mammals. Ecology. 2014; doi: https://doi.org/10.1890/13-1917.1.

66. Global Biodiversity Information Facility: GBIF. https://www.gbif.org/ (2022). Accessed 2 May 2021.

67. Borges R, Machado JP, Gomes C, Rocha AP, Antunes A. Measuring phylogenetic signal between categorical traits and phylogenies. Bioinformatics. 2019; doi: 10.1093/bioinformatics/bty800.

68. Sulzner K, Fiorello C, Ridgley F, Garelle D, Deem SL. Conservation medicine and One Health in zoos: Scope, obstacles, and unrecognized potential. Zoo Biol. 2021; doi: 10.1002/zoo.21572.

69. Tuck N. Animals in Moral Limbo: How Literary Pigs May Help Lab-Generated Ones. Animals. 2020; doi: 10.3390/ani10040629.

70. IUCN: The IUCN Red List of Threatened Species. Version 2021-1. IUCN Red List Threat. Species. https://www.iucnredlist.org/en (2021). Accessed 2 May 2021.

71. Wikipedia: List of domesticated animals. https://en.wikipedia.org/w/index.php?title=List_of_domesticated_animals&oldid=1020505988 (2021). Accessed 2 May 2021.

72. Chamberlain S (2020). rredlist (Version 0.7.0) https://github.com/ropensci/rredlist

73. Colléony A, Clayton S, Couvet D, Saint Jalme M, Prévot A-C. Human preferences for species conservation: Animal charisma trumps endangered status. Biol Conserv. 2017; doi: 10.1016/j.biocon.2016.11.035.

74. Google: Google Trends. Google Trends. https://trends.google.com/trends/?geo=AU (2021). Accessed 2 May 2021.

75. Massicotte P, Eddelbuettel D (2020). gtrendsR (Version 1.4.7) https://github.com/PMassicotte/gtrendsR

Tables

TABLE 1 Details of hypotheses.

Potential driver	Hypothesis and rationale	Statistical surrogate	Data source
Size of species	We predict that higher body masses correlate with higher species *h*-index. Larger mammals, i.e. megafaunal species such as elephants and rhinoceroses, receive more research interest because they are generally considered as more 'charismatic' [8,46].	Body mass (transformed with $\log_{10}$)	Wilman et al. [65]
Location of natural habitat	We predict that species found in temperate latitudes have higher species *h*-index. Mammals near the temperate zones attract more research interest as more researchers originate from these areas, such as North America, Europe, Australia, New Zealand, and southern Africa [5]. Thus, mammals whose natural habitat are within these regions are better studied.	Median latitude	GBIF [66]
Phylogenetic relatedness	We predict that there are phylogenetic signals present in the dataset. Mammals that are more phylogenetically related receive similar species *h*-index because related species share similar traits that may influence the propensity of researchers to study members of a given clade [67]. Furthermore, species closer to humans will be over-represented in species with high *h*-index values [11].	Branch lengths of phylogenetic tree	Upham et al. [35]

Human use & Domestication	We predict that mammals with more human uses and domesticated mammals have higher species *h*-index. Some examples of human uses include transportation (e.g. horses and elephants), companionship (e.g. cats and dogs), food products (e.g. sheep and cattle), etc. Lab animals (e.g. rabbits and rodents) are likely to receive most research interest since the main purpose of keeping these animals is for scientific research [68,69].	IUCN Red List human use categories & Wikipedia list of domesticated species	IUCN Red List [70], & Wikipedia [71]
Demography	We predict a u-shaped distribution of species *h*-index, where species in the 'Least Concern' and 'Critically Endangered' categories receive higher species *h*-index. Previous studies showed no correlations between the mammals' IUCN Red List status and their research interest [16,17,19].	IUCN Red List status	IUCN Red List [70]; cleaned with *rredlist* [72]
General interest	We predict that more general interest correlates with higher species *h*-index. Research and general interests are highly correlated since we tend to be more attracted to 'charismatic' species, such as lions and elephants [46], and are more willing to donate for their conservation causes [73], resulting in more research interest.	Google Trends index	Google Trends [74]; extracted with *gtrendsR* [75]

We predicted that species *h*-index can be influenced by body sizes, location of natural habitat, phylogeny, human uses and domestication, demography, and general interest.

TABLE 2 Summary of statistical results from the Bayesian generalized linear mixed model (BGLMM).

**Estimate**	**Mean**	**95% Credible Interval (CI)**
Fixed effects		
Intercept	1.310	-0.119, 2.743
$\log_{10}$(Body mass)	0.097	0.037, 0.155
Latitude (absolute value)	0.022	0.019, 0.025
IUCN Red List status (1st degree polynomial)	-16.397	-19.323, -13.569
IUCN Red List status (2nd degree polynomial)	2.759	0.323, 5.206
Human use	0.268	0.166, 0.371
Domestication status	-0.376	-0.547, -0.206
$\log_{10}$(Google Trends)	0.490	0.457, 0.522
Random effects		
Phylogeny	1.601	1.080, 2.230
Non-phylogeny	0.807	0.745, 0.871
Phylogenetic heritability (H^2)	0.637 (*0.642)	0.000, 0.660 (*0.516, 0.660)

*Phylogenetic signal after removing 1124 species (20.4%) from the tree that showed no signals.

The distributions here follow the distributions stated in the hypothesis.

TABLE 3 Summary of statistical results from the Bayesian generalized linear mixed model (BGLMM).

Estimate	Mean	95% Credible Interval (CI)
Fixed effects		
Intercept	0.149	-1.163, 1.451
$\log_{10}$(Body mass)	0.105	0.043, 0.165
Latitude (absolute value)	0.022	0.019, 0.025
IUCN Red List status (1st degree polynomial)	-16.539	-19.453, -13.692
IUCN Red List status (2nd degree polynomial)	3.021	0.581, 5.469
Human use	0.277	0.171, 0.382
$\log_{10}$(Google Trends)	0.501	0.469, 0.534
Random effects		
Phylogeny	1.573	1.055, 2.209
Non-phylogeny	0.818	0.755, 0.882
Phylogenetic heritability (H^2)	0.626 (*0.633)	0.000, 0.652 (*0.510, 0.653)

*Phylogenetic signal after removing 1124 species (21.0%) from the tree that showed no signals.

The distributions here follow the distributions stated in the hypothesis, except we removed domestication status and domesticated and partially-domesticated species.

Figures

FIGURE 1 Species h-index of mammals.

Plot (a) shows 34 mammals with h = 100 or more, representing 6 different orders marked by dots of different colours. Figure in the inset shows the distribution of species h-index of all mammals, with the species scoring above h = 100 or more marked by the red box. Plot (b) also shows the mammals with h = 100 or more, but removes domesticated species, with 17 species left.

FIGURE 2 The changes in mammalian literature from 1940 to 28 April 2021.

(a) The number of publications per year for 30 mammalian orders and the proportion of species per order from the collated mammalian dataset represented by the doughnut chart, and (b) change in the frequency of publications on 30 mammalian orders present in the dataset. Total number of mammalian species analysed is 7,521.

FIGURE 3 Centroids of global distributions of 4,744 mammalian species.

(a) The distribution of non-threatened species listed as 'Least Concern'. (b) The distribution of threatened species listed as 'Vulnerable', 'Near Threatened', 'Endangered', 'Critically Endangered', and 'Extinct in the Wild'. The species' corresponding h-index values are illustrated by dot colour.

FIGURE 4 Relationship between predictor variables and species h-index values.

(i) (a) Species average body mass (n = 5,158 species, fitted curves represent 50% quantile for each clade), (b) Median latitude of species geographical distribution (n = 4,435 species, fitted

curve from generalised additive model (GAM) with shaded grey area representing 95% confidence interval; density bar on top of the plot illustrates the number of species at each latitude). (c) Human use categories (n = 7,521, nNo documented use = 6,124, and nUse documented = 1,397). (d) Domestication status (n = 7,521 species, nDomesticated = 12, nPartially-domesticated = 136, and nWild = 7373). (e) IUCN Red List status (n = 5,584 species, nLeast Concern = 3,152, nNear Threatened = 340, nVulnerable = 530, nEndangered = 512, nCritically Endangered = 208, and nExtinct in the Wild = 2). (f) Google Trends Index summed for each species (n = 7,521 species, nGoogle Trends Index > 0 = 1,323, and nGoogle Trends Index = 0 = 6,124 species). Box plots in (c), (d), and (e) show the median, 25th and 75th percentiles, and lower and upper extremes. (ii) showing the same data as (i), but each species is coloured according to their domestication status.

Click here to access/download;Figure;Figure 1.pdf

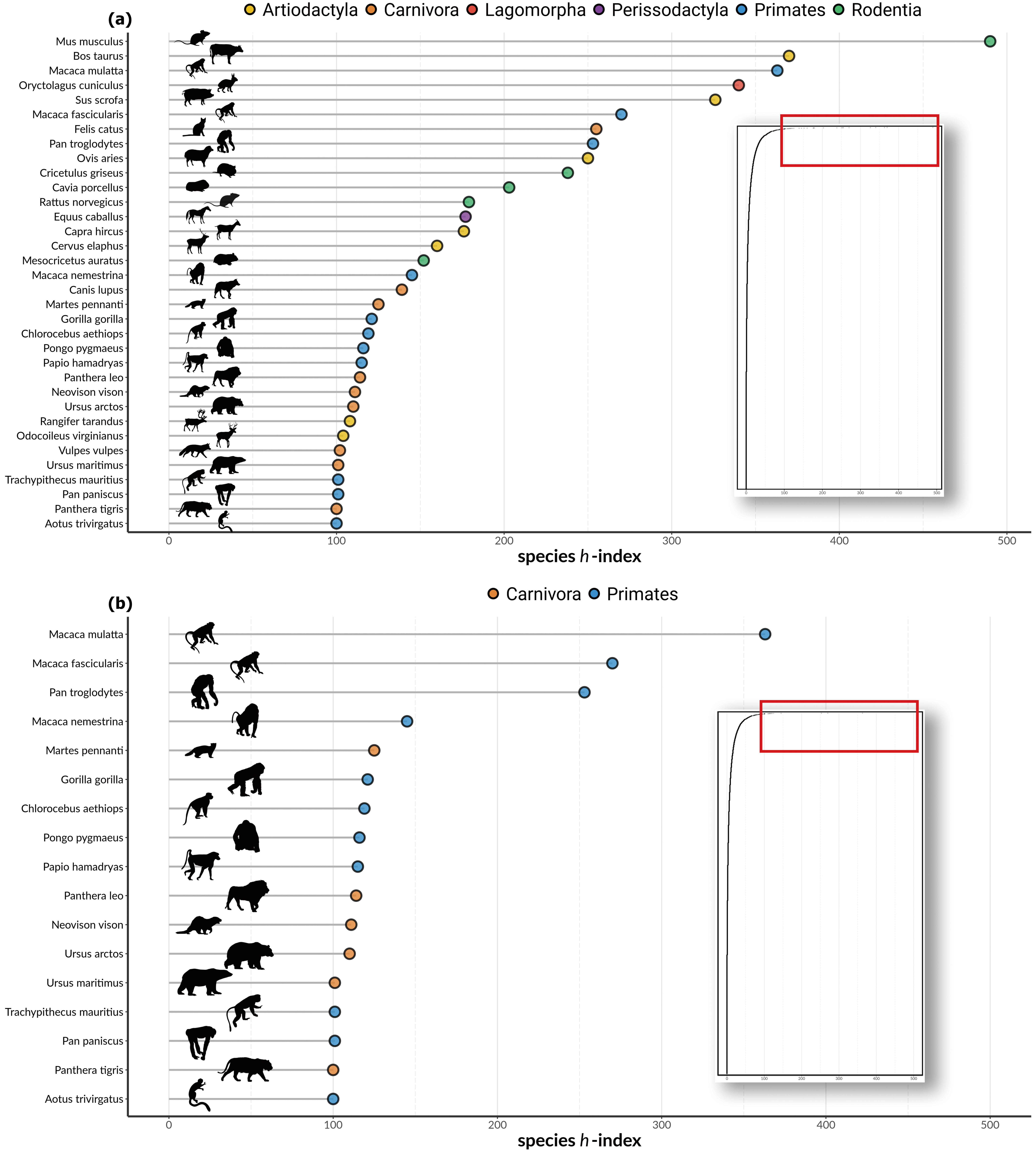

Click here to access/download;Figure;Figure 2.pdf

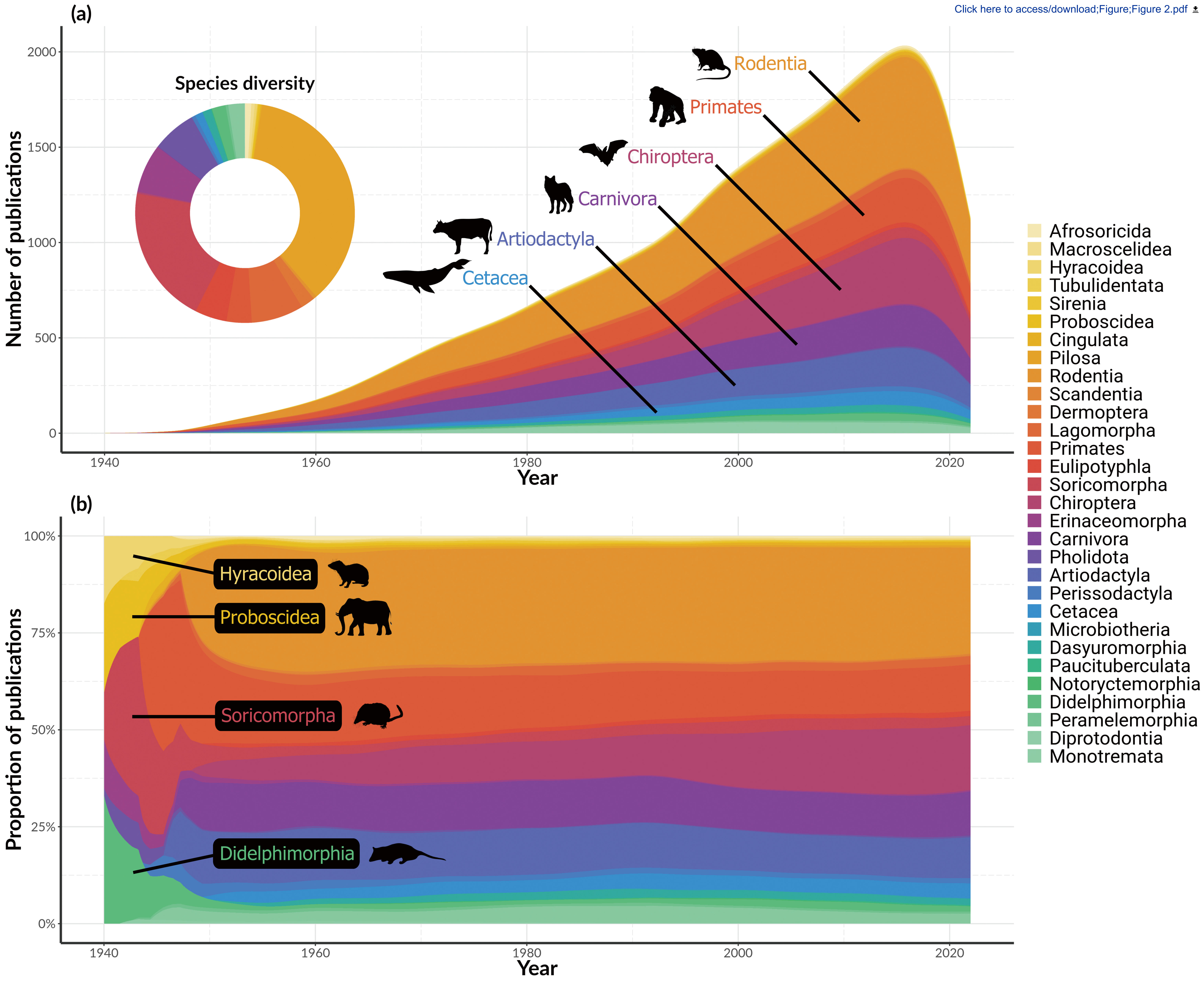

Click here to access/download;Figure;Figure 3.png

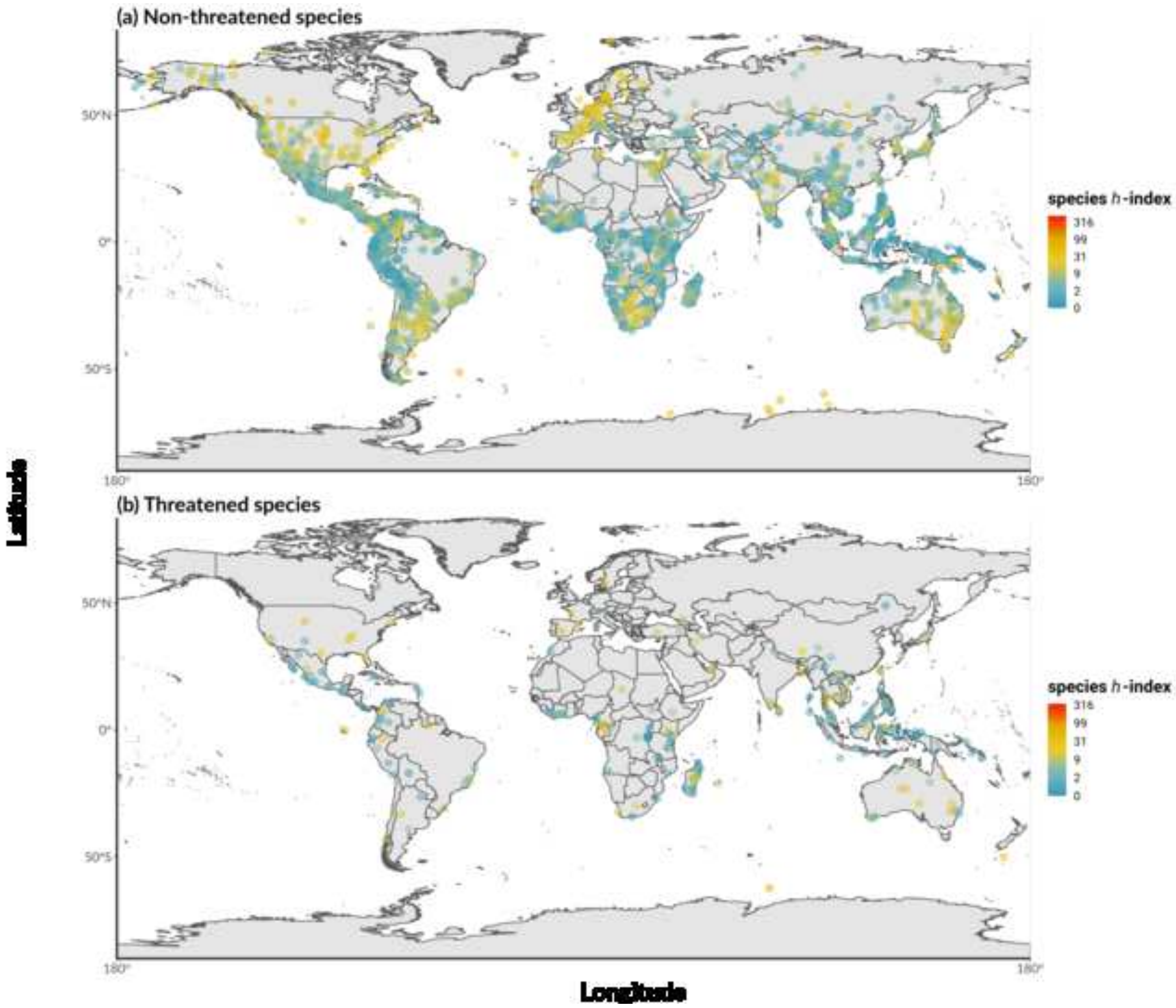

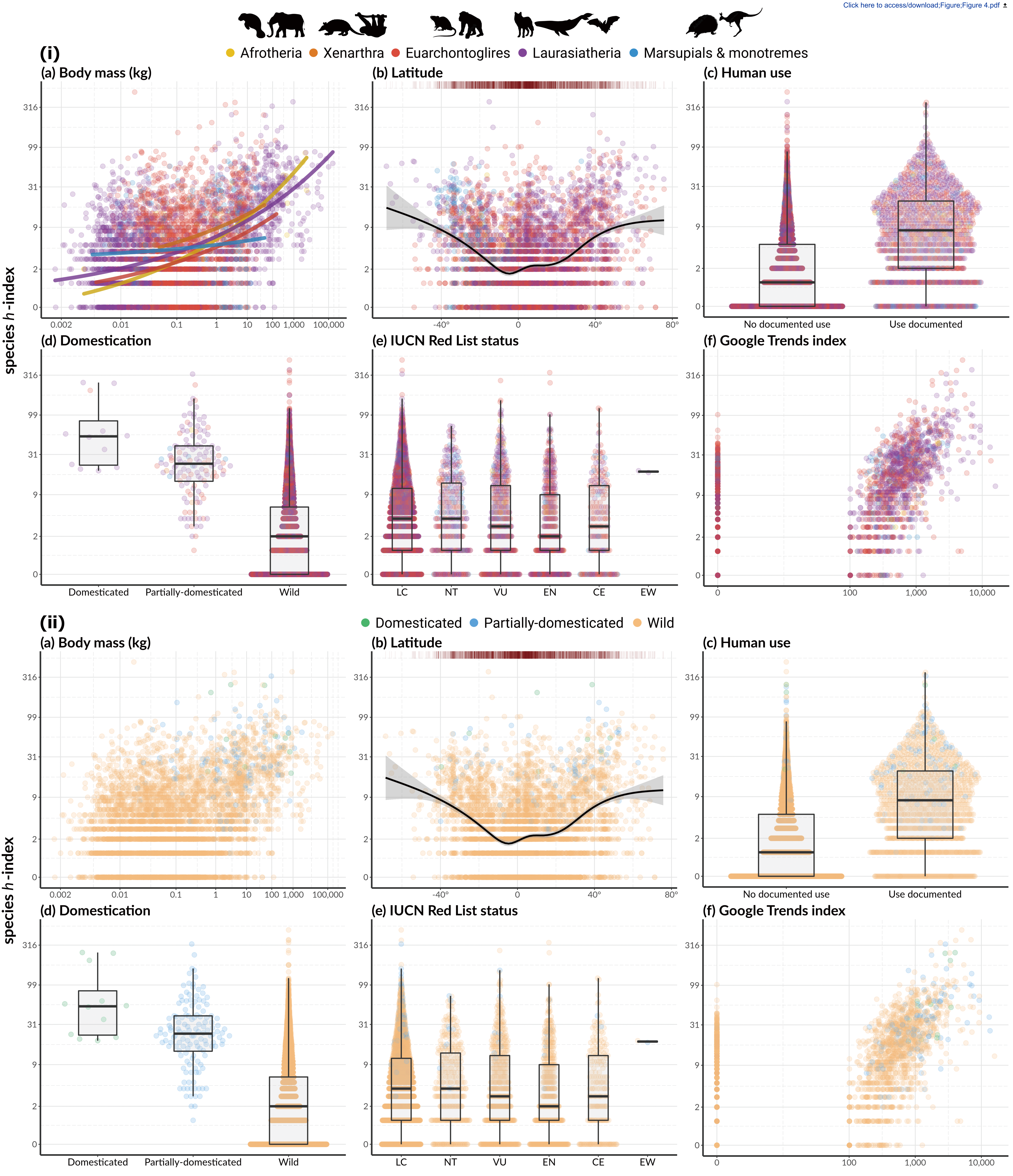
(i)
Afrotheria
Xenarthra
Euarchontoglires
Laurasiatheria
Marsupials & monotremes
(a) Body mass (kg)
(b) Latitude
(c) Human use
(d) Domestication
(e) IUCN Red List status
(f) Google Trends index
species h -index
No documented use
Use documented
Domesticated
Partially-domesticated
Wild
LC
NT
VU
EN
CE
EW
(ii)
Domesticated
Partially-domesticated
Wild
(a) Body mass (kg)
(b) Latitude
(c) Human use
(d) Domestication
(e) IUCN Red List status
(f) Google Trends index
species h -index

Click here to access/download
Supplementary Material
Supplementary Material text.docx

Click here to access/download
Supplementary Material
Figure S1_supplementary material.png

Click here to access/download
Supplementary Material
Figure S2_supplementary material.pdf

Click here to access/download
Supplementary Material
Figure S4_supplementary material.png

Click here to access/download
Supplementary Material
Figure S5_supplementary material.pdf

Click here to access/download
Supplementary Material
Figure S6_supplementary material.pdf

Click here to access/download
Supplementary Material
Figure S7_supplementary material.pdf

Click here to access/download
Supplementary Material
Figure S8_supplementary material.pdf

Click here to access/download
Supplementary Material
Supplementary Material text.pdf
