# Supplementary material for: Quantifying research interests in 7,521 mammalian species with h-index: a case study
Source: Gigascience. 2022 Aug 13;11:giac074. doi: 10.1093/gigascience/giac074 (PMC9375528; doi:10.1093/gigascience/giac074)
Supplement: giac074_Supplemental_Files [file giac074_supplemental_files.zip › Figure S8_supplementary material.pdf]

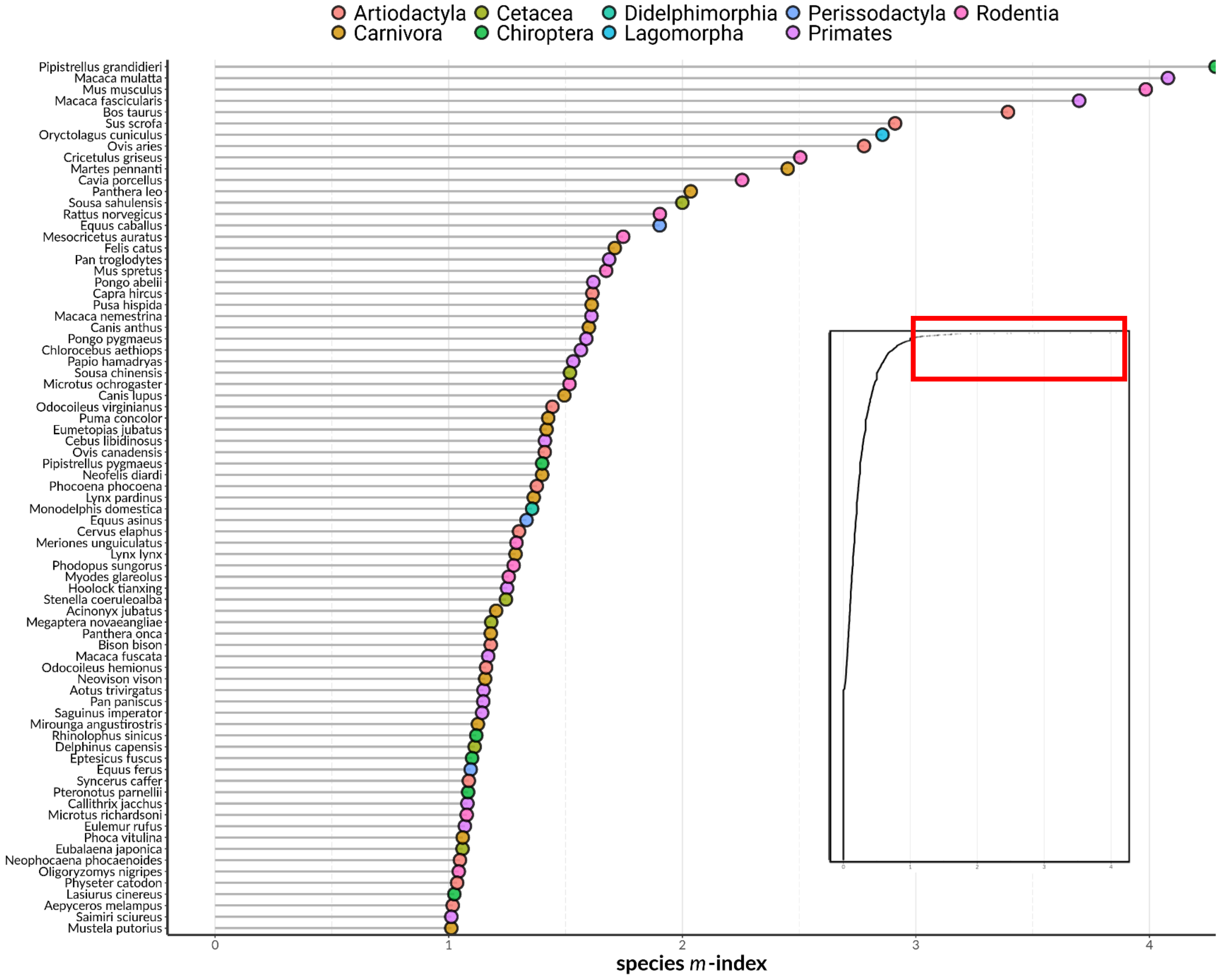
Artiodactyla
Carnivora
Cetacea
Chiroptera
Didelphimorphia
Lagomorpha
Perissodactyla
Primates
Rodentia
Pipistrellus grandidieri
Macaca mulatta
Mus musculus
Macaca fascicularis
Bos taurus
Sus scrofa
Oryctolagus cuniculus
Ovis aries
Cricetulus griseus
Martes pennanti
Cavia porcellus
Panthera leo
Sousa sahulensis
Rattus norvegicus
Equus caballus
Mesocricetus auratus
Felis catus
Pan troglodytes
Mus spretus
Pongo abelii
Capra hircus
Pusa hispida
Macaca nemestrina
Canis anthus
Pongo pygmaeus
Chlorocebus aethiops
Papio hamadryas
Sousa chinensis
Microtus ochrogaster
Canis lupus
Odocoileus virginianus
Puma concolor
Eumetopias jubatus
Cebus libidinosus
Ovis canadensis
Pipistrellus pygmaeus
Neofelis diardi
Phocoena phocoena
Lynx pardinus
Monodelphis domestica
Equus asinus
Cervus elaphus
Meriones unguiculatus
Lynx lynx
Phodopus sungorus
Myodes glareolus
Hoolock tianxing
Stenella coeruleoalba
Acinonyx jubatus
Megaptera novaeangliae
Panthera onca
Bison bison
Macaca fuscata
Odocoileus hemionus
Neovison vison
Aotus trivirgatus
Pan paniscus
Saguinus imperator
Mirounga angustirostris
Rhinolophus sinicus
Delphinus capensis
Eptesicus fuscus
Equus ferus
Syncerus caffer
Pteronotus parnellii
Callithrix jacchus
Microtus richardsoni
Eulemur rufus
Phoca vitulina
Eubalaena japonica
Neophocaena phocaenoides
Oligoryzomys nigripes
Physeter catodon
Lasiurus cinereus
Aepyceros melampus
Saimiri sciureus
Mustela putorius
0
1
2
3
4
species m-index
