# Supplementary material for: Quantifying research interests in 7,521 mammalian species with h-index: a case study
Source: Gigascience. 2022 Aug 13;11:giac074. doi: 10.1093/gigascience/giac074 (PMC9375528; doi:10.1093/gigascience/giac074)
Supplement: giac074_Supplemental_Files [file giac074_supplemental_files.zip › Supplementary Material text.docx]

# **Supplementary Material**

**FIGURE S1** Summary of the data collected, and treatments assigned for analysis.

Completeness of data is the proportion of available data out of n = 7,521.

**FIGURE S2** Graphical explanation of the *h*-index.

The *h*-index is obtained by ranking the citations of each paper in descending order and then finding the number of papers (*h*) with *h* number of citations. The 6^th^ most cited publication has been cited at least 6 times, as indicated by the blue dotted lines. Hence, the *h*-index is 6.

**TABLE S3** Variance inflation factors of the moderators from the quasi-Poisson generalised linear model (GLM).

| **Moderator** | **Variance inflation factor** |
| --- | --- |
| log_10_(Body mass) | 1.670 |
| Latitude (absolute value) | 1.177 |
| log_10_(Google Trends index) | 1.565 |
| (IUCN Red List status)^2^ | 1.085 |
| Human use | 1.491 |
| Domestication | 1.122 |

**FIGURE S4** Frequency of species with each species *h*-index. n_all_ = 7,521, n*_h_* _= 0_ = 2,426.

**FIGURE S5** Distribution of individual species *h*-index for all IUCN Red List categories.

Box plots show the median, 25^th^ and 75^th^ percentiles, and lower and upper extremes.

**FIGURE S6** Distribution of individual species *h*-index for all human use categories.

Box plots show the median, 25^th^ and 75^th^ percentiles, and lower and upper extremes.

**FIGURE S7** Phylogenetic tree of 5,497 mammalian species included in the analyses.

5 major clades are shown in different colours. Silhouettes representing the top 9 mammals with the highest species *h*-index.

**FIGURE S8** Species *m*-index of mammals.

The plot shows the mammals with *m* > 1, representing 9 different orders marked by dots of different colors. Figure in the inset shows the distribution of species *m*-index of all mammals, with the species scoring above *m* > 1 or more marked by the red box.
