# Supplementary material for: Quantifying research interests in 7,521 mammalian species with h-index: a case study
Source: Gigascience. 2022 Aug 13;11:giac074. doi: 10.1093/gigascience/giac074 (PMC9375528; doi:10.1093/gigascience/giac074)
Supplement: giac074_Supplemental_Files [file giac074_supplemental_files.zip › Supplementary Material text.pdf]

Supplementary Material

FIGURE S1 Summary of the data collected, and treatments assigned for analysis.

Completeness of data is the proportion of available data out of n = 7,521.

FIGURE S2 Graphical explanation of the *h*-index.

The *h*-index is obtained by ranking the citations of each paper in descending order and then finding the number of papers (*h*) with *h* number of citations. The 6th most cited publication has been cited at least 6 times, as indicated by the blue dotted lines. Hence, the *h*-index is 6.

TABLE S3 Variance inflation factors of the moderators from the quasi-Poisson generalised linear model (GLM).

Moderator	Variance inflation factor
log_{10}(Body mass)	1.670
Latitude (absolute value)	1.177
log_{10}(Google Trends index)	1.565
(IUCN Red List status)^2	1.085
Human use	1.491
Domestication	1.122

FIGURE S4 Frequency of species with each species *h*-index. n_{all} = 7,521, $n_{h=0}$ = 2,426.

FIGURE S5 Distribution of individual species *h*-index for all IUCN Red List categories.

Box plots show the median, 25th and 75th percentiles, and lower and upper extremes.

FIGURE S6 Distribution of individual species *h*-index for all human use categories.

Box plots show the median, 25th and 75th percentiles, and lower and upper extremes.

FIGURE S7 Phylogenetic tree of 5,497 mammalian species included in the analyses.

5 major clades are shown in different colours. Silhouettes representing the top 9 mammals with the highest species *h*-index.

FIGURE S8 Species *m*-index of mammals.

The plot shows the mammals with $m > 1$, representing 9 different orders marked by dots of different colors. Figure in the inset shows the distribution of species *m*-index of all mammals, with the species scoring above $m > 1$ or more marked by the red box.
