# Supplementary figures and images for: Quantifying research interests in 7,521 mammalian species with h-index: a case study
Source: Gigascience. 2022 Aug 13;11:giac074. doi: 10.1093/gigascience/giac074 (PMC9375528; doi:10.1093/gigascience/giac074)

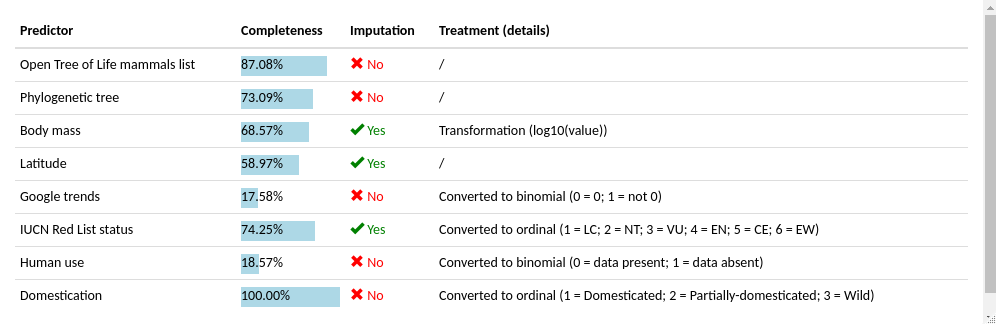

Supplement: giac074_Supplemental_Files [file giac074_supplemental_files.zip › Figure S1_supplementary material.png]

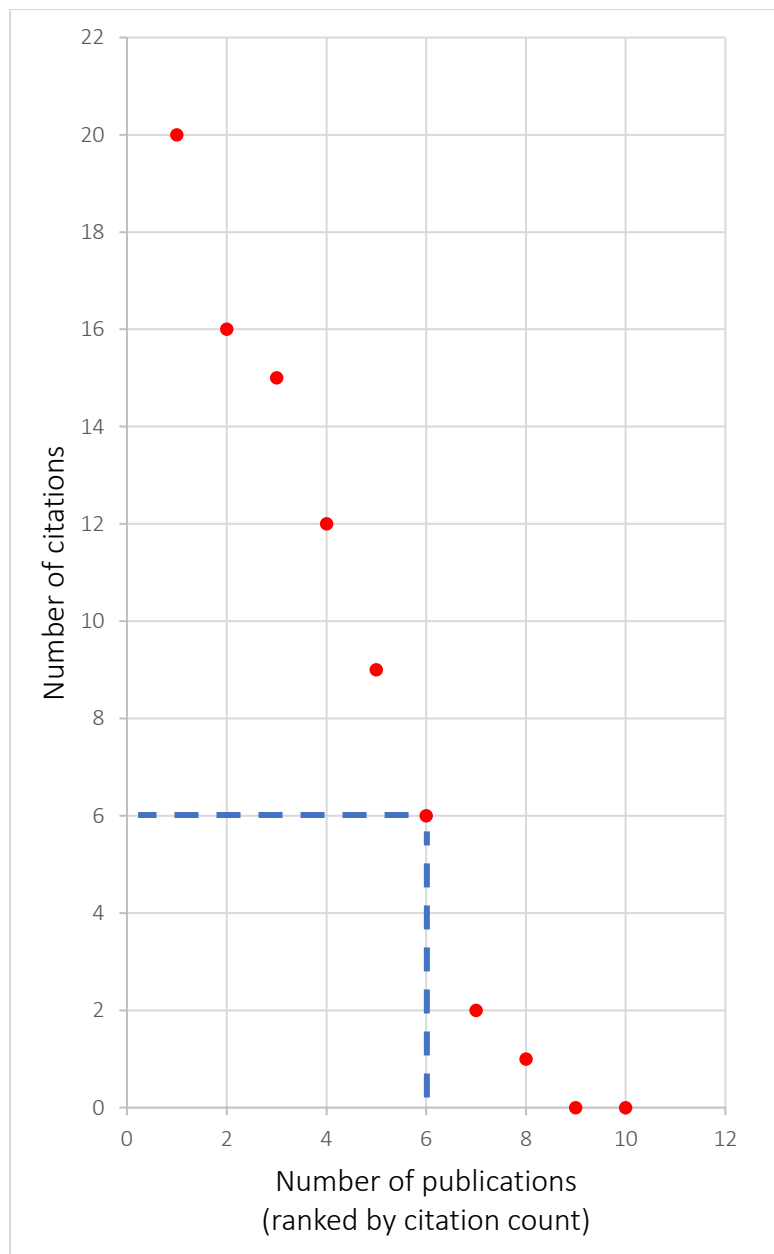

Supplement: giac074_Supplemental_Files [file giac074_supplemental_files.zip › Figure S2_supplementary material.pdf]

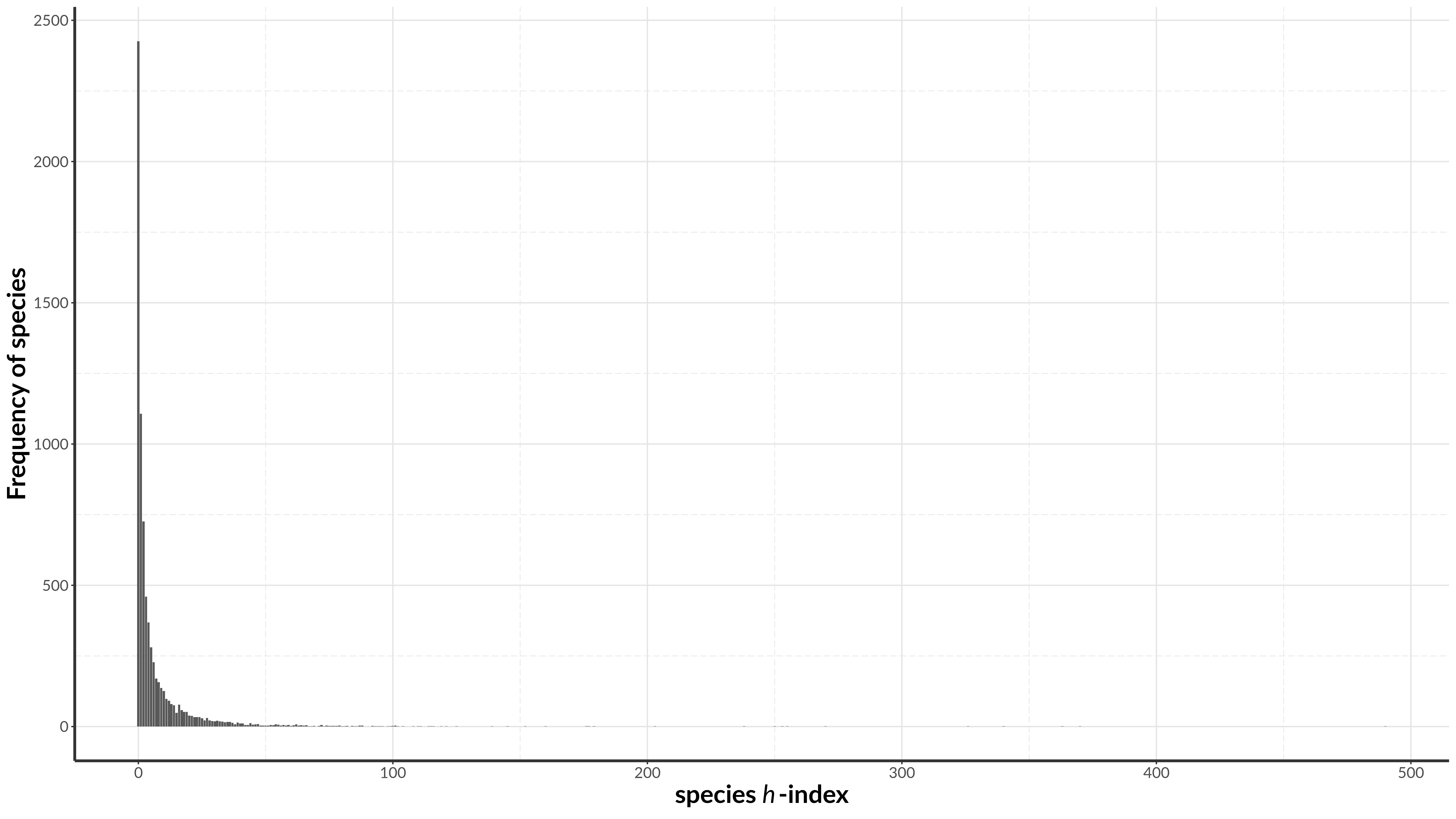

Supplement: giac074_Supplemental_Files [file giac074_supplemental_files.zip › Figure S4_supplementary material.png]

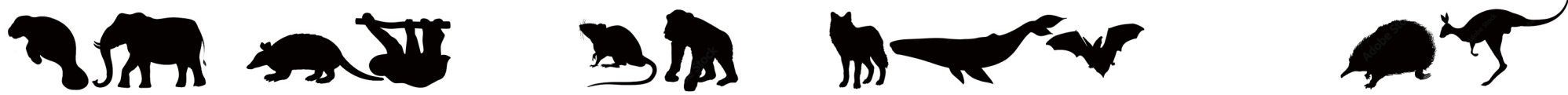

● Afrotheria ● Xenarthra ● Euarchontoglires ● Laurasiatheria ● Marsupials & monotremes

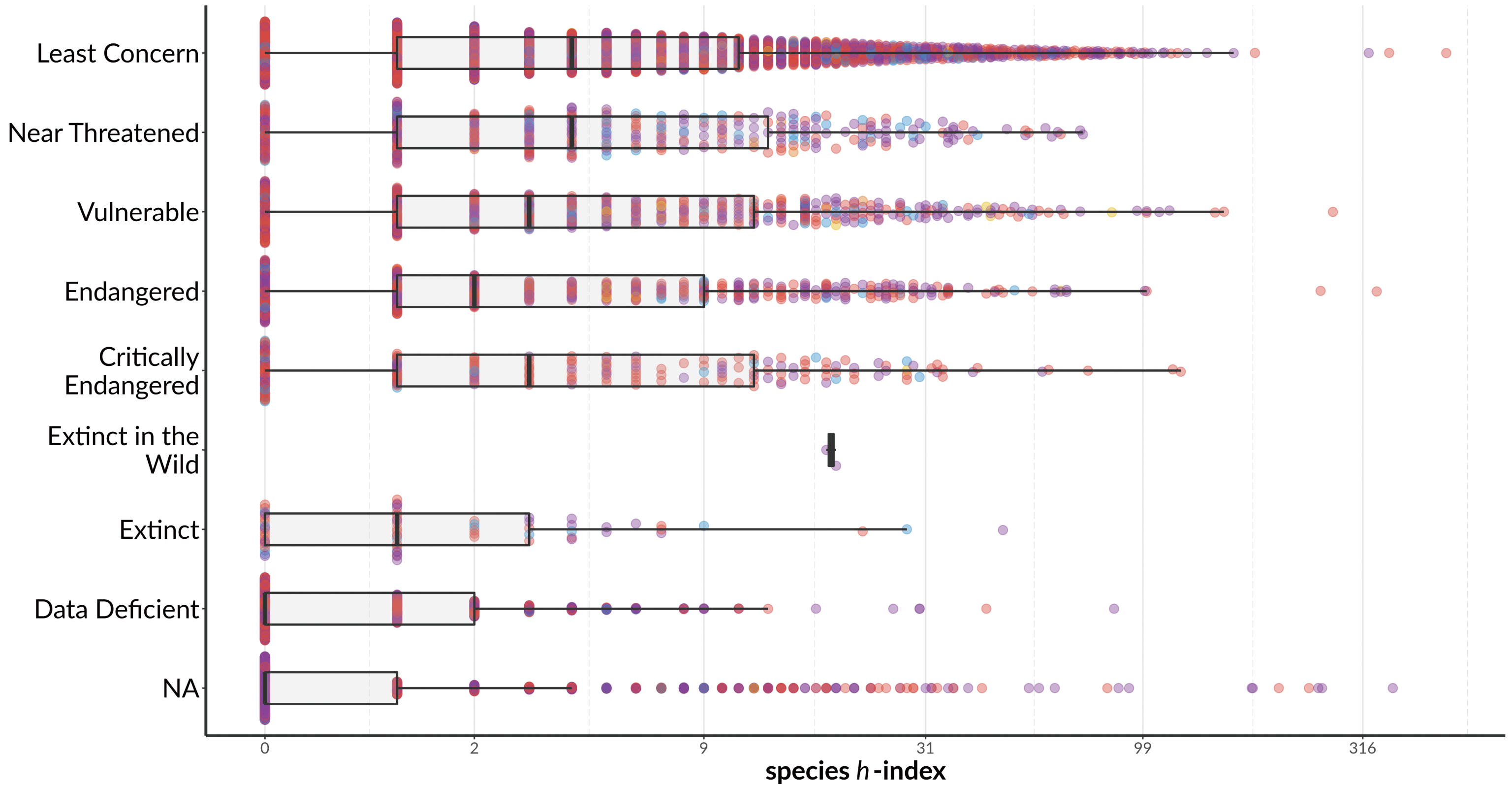

Supplement: giac074_Supplemental_Files [file giac074_supplemental_files.zip › Figure S5_supplementary material.pdf]

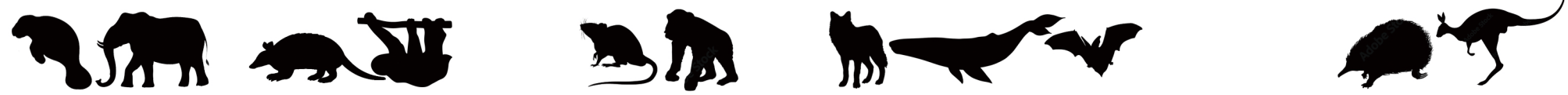

● Afrotheria ● Xenarthra ● Euarchontoglires ● Laurasiatheria ● Marsupials & monotremes

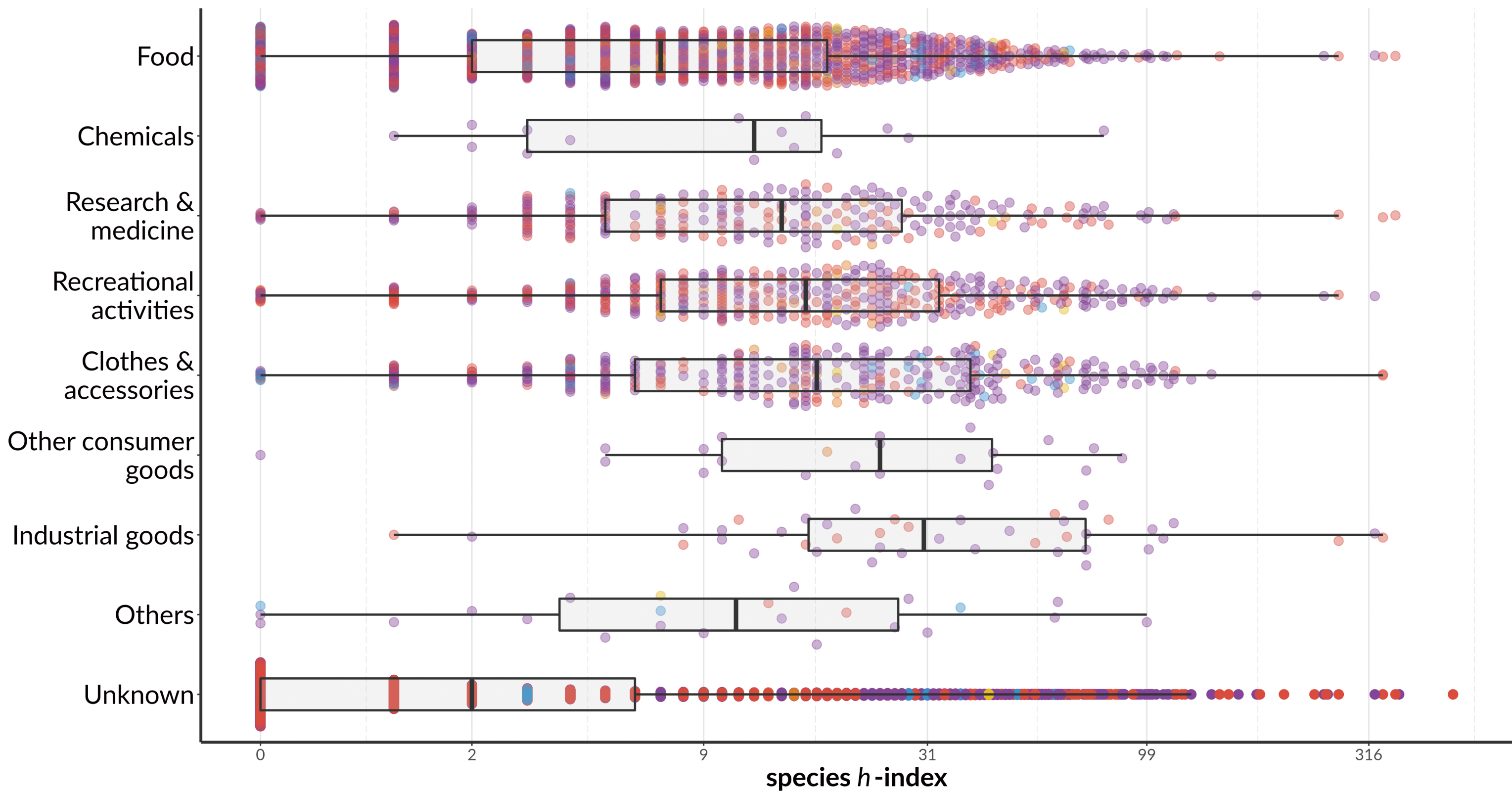

Supplement: giac074_Supplemental_Files [file giac074_supplemental_files.zip › Figure S6_supplementary material.pdf]

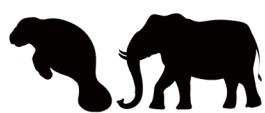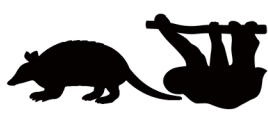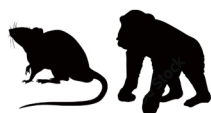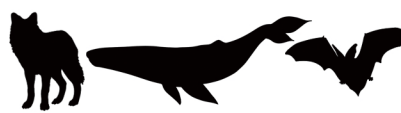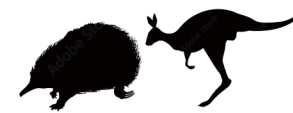

● Afrotheria ● Xenarthra ● Euarchontoglires ● Laurasiatheria ● Marsupials & monotremes

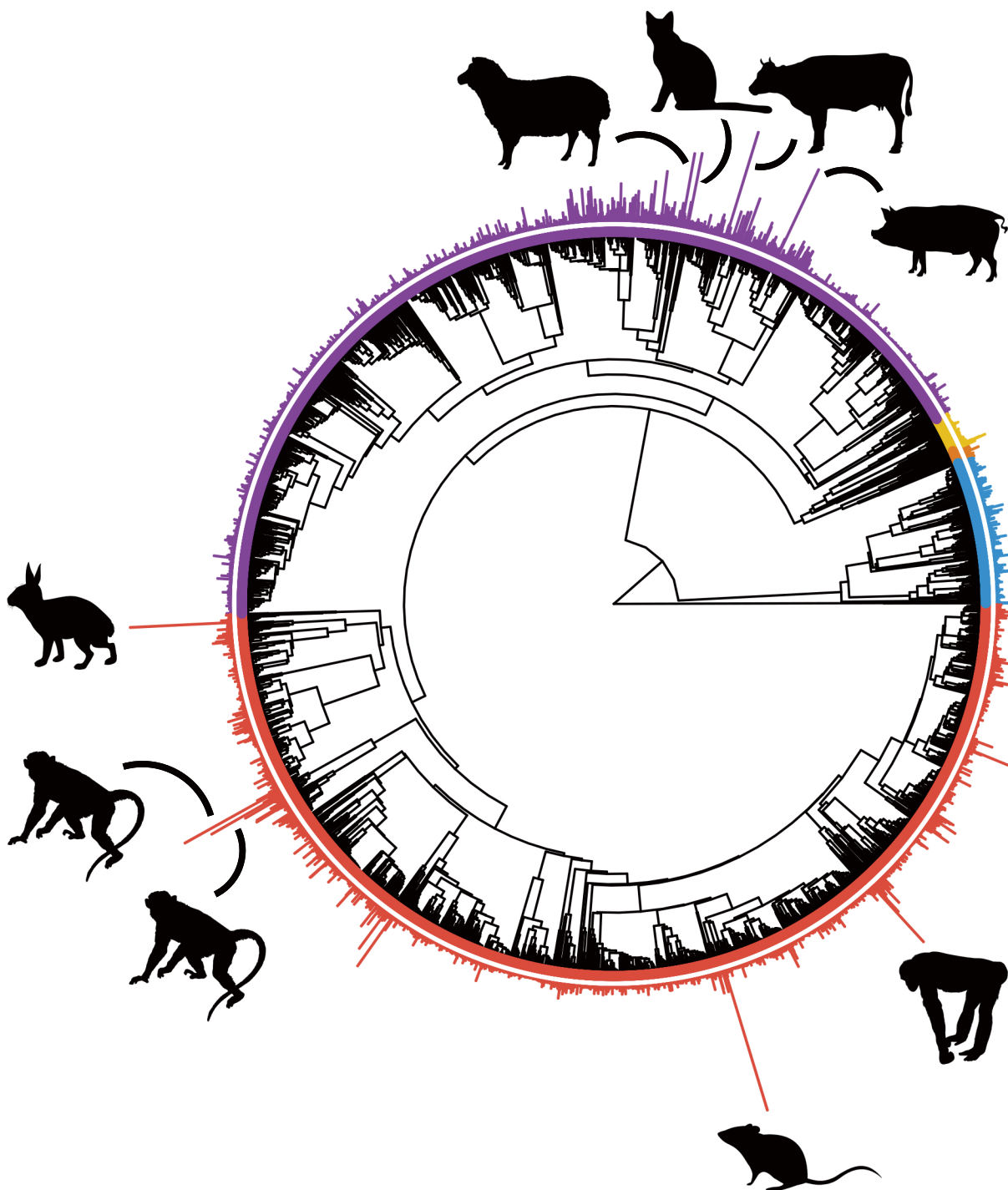

Supplement: giac074_Supplemental_Files [file giac074_supplemental_files.zip › Figure S7_supplementary material.pdf]
