# Supplementary material for: Quantifying research interests in 7,521 mammalian species with h-index: a case study
Source: Gigascience. 2022 Aug 13;11:giac074. doi: 10.1093/gigascience/giac074 (PMC9375528; doi:10.1093/gigascience/giac074)
Supplement: giac074_Response_to_Reviewer_Comments_Original_Submission [file giac074_response_to_reviewer_comments_original_submission.pdf]

Author's Response To Reviewer Comments

Close

We have compiled the comments into a PDF documented. It has been uploaded as a 'Personal cover', with the description of 'Addressing reviewers' comments', and document name 'Manuscript comments'.

Close
