# Supplementary material for: Quantifying research interests in 7,521 mammalian species with h-index: a case study
Source: Gigascience. 2022 Aug 13;11:giac074. doi: 10.1093/gigascience/giac074 (PMC9375528; doi:10.1093/gigascience/giac074)
Supplement: giac074_Response_to_Reviewer_Comments_Revision_1 [file giac074_response_to_reviewer_comments_revision_1.pdf]

Author's Response To Reviewer Comments

Close

Our reply to reviewers comments is attached as item type 'Personal Cover' with the description of 'Reviewers comments reply letter'.

Close
