# Supplementary material for: Quantifying research interests in 7,521 mammalian species with h-index: a case study
Source: Gigascience. 2022 Aug 13;11:giac074. doi: 10.1093/gigascience/giac074 (PMC9375528; doi:10.1093/gigascience/giac074)
Supplement: giac074_Response_to_Reviewer_Comments_Revision_2 [file giac074_response_to_reviewer_comments_revision_2.pdf]

# Author's Response To Reviewer Comments

Close

Attached are the word documents of the main text and supplementary material. I have now properly cited the databases required.

Close
