# Supplementary material for: Quantifying research interests in 7,521 mammalian species with h-index: a case study
Source: Gigascience. 2022 Aug 13;11:giac074. doi: 10.1093/gigascience/giac074 (PMC9375528; doi:10.1093/gigascience/giac074)
Supplement: giac074_Reviewer_1_Report_Original_Submission — Emilio Berti -- 12/17/2021 Reviewed [file giac074_reviewer_1_report_original_submission.pdf]

Reviewer Report

Title: Quantifying research interests in 7,521 mammalian species with h-index: a case study

Version: Original Submission **Date:** 12/17/2021

Reviewer name: Emilio Berti

Reviewer Comments to Author:

I have finished the review of the manuscript entitled "Quantifying research interests in 7,521 mammalian species with h-index: a case study". I found this to be a great contribution that advances current knowledge and provides a much needed approach to study issues related to taxonomic bias and their implications for research and conservation.

In particular, I can see two major contributions: 1) the study adds to current knowledge of taxonomic biases in mammals, highlighting the problems in quantifying and studying such biases and delivering new results that highlight how widespread such biases are and what they imply for basic research and conservation of biodiversity. 2) the study provides a very well-thought and flexible approach, together with code to perform similar analyses and to expand them. I can see this approach to set a new standard on how to investigate taxonomic biases for future studies.

The manuscript is very well-written and I enjoyed reading it very much. All conclusions are supported by the data and all statistical analyses are clear and fit well the questions asked. In particular, I really enjoyed how the authors structured the Discussion, which clearly put the results in a broad context and that is easy to understand. The authors also acknowledged all potential limitations of the approach used very honestly, provided several alternatives to account for them in future studies and proposed many interesting and important suggestions to advance the field.

I do not have any major suggestion that could add to the quality and importance of the study. I have few minor suggestions, listed below, to improve small parts of the manuscript.

I have been studying taxonomic harmonization quite a lot lately and I was very glad to see that the authors are also concerned of the issues with taxonomic backbones. In particular, the authors acknowledge in the Discussion that taxonomic backbones are not fixed and that they change over time and have sometimes unresolved synonyms. I think this is a very well-written paragraph and address the major potential issues with taxonomic backbones. I believe these potential biases to be largely negligible for this study, considering its aims and the thourough workflow that seemed to have been put in this step. I would, however, expand briefly in the Method section on how taxonomic names of species were obtained in order to give more details of this step. This would help to understand better how this was performed, which may also help many readers to perform similar analyses. Specifically, names were obtained from the Open Tree of Life (OTL) accessed through the R package rcol (Catalogue of Life). I would suggest to briefly expand here on how this search was performed and how synonyms were resolved, e.g. by stating which functions were used and how synonyms were combined (e.g. is there a direct function to retrieve an accepted name, or was this done with a custom function?). I would also include the version of rcol used and the access time (year/month) to OTL (through COL?), as taxonomic backbones can change over time and results from queries can also change depending on the version of

the package.
In the first paragraph of the Results, the authors give some interesting statistics, namely how many species have an h-index = 0 and how many species have an h-index >= 100. These are very intuitive statistics that tell much to the reader. I wonder if including also the median would be a nice addition, as it will give an additional metric that is also intuitive to understand and that would complement the two already given.
In the Results, I think 'Appendix Table 2' was moved to the main text and should be 'Table 2'.
I found Figure 3 (map plot with species h-index and IUCN status) to be a bit hard to follow. In particular, I cannot distinguish very well among IUCN status, which are here represented by different point sizes. As points often overlap, the size is sometimes hard to understand. Perhaps faceting it by IUCN status could help, maybe also grouping VU, EN, CE into a 'threaten' class to avoid having five maps.
Sincerely,
Emilio Berti
German Centre for Integrative Biodiversity Research (iDiv) Halle-Jena-Leipzig
Theory in Biodiversity Science
04103 Leipzig, Germany
emilio.berti@idiv.de

Methods

Are the methods appropriate to the aims of the study, are they well described, and are necessary controls included? Choose an item.

Conclusions

Are the conclusions adequately supported by the data shown? Choose an item.

Reporting Standards

Does the manuscript adhere to the journal's guidelines on minimum standards of reporting? Choose an item.

Choose an item.

Statistics

Are you able to assess all statistics in the manuscript, including the appropriateness of statistical tests used? Choose an item.

Quality of Written English

Please indicate the quality of language in the manuscript: Choose an item.

Declaration of Competing Interests

Please complete a declaration of competing interests, considering the following questions:

- Have you in the past five years received reimbursements, fees, funding, or salary from an organisation that may in any way gain or lose financially from the publication of this manuscript, either now or in the future?
- Do you hold any stocks or shares in an organisation that may in any way gain or lose financially from the publication of this manuscript, either now or in the future?
- Do you hold or are you currently applying for any patents relating to the content of the manuscript?
- Have you received reimbursements, fees, funding, or salary from an organization that holds or has applied for patents relating to the content of the manuscript?
- Do you have any other financial competing interests?
- Do you have any non-financial competing interests in relation to this paper?

If you can answer no to all of the above, write 'I declare that I have no competing interests' below. If your reply is yes to any, please give details below.

I declare that I have no competing interests

I agree to the open peer review policy of the journal. I understand that my name will be included on my report to the authors and, if the manuscript is accepted for publication, my named report including any attachments I upload will be posted on the website along with the authors' responses. I agree for my report to be made available under an Open Access Creative Commons CC-BY license (http://creativecommons.org/licenses/by/4.0/). I understand that any comments which I do not wish to be included in my named report can be included as confidential comments to the editors, which will not be published.

Choose an item.

To further support our reviewers, we have joined with Publons, where you can gain additional credit to further highlight your hard work (see: https://publons.com/journal/530/gigascience). On publication of this paper, your review will be automatically added to Publons, you can then choose whether or not to claim your Publons credit. I understand this statement.

Yes Choose an item.
