# Supplementary material for: Quantifying research interests in 7,521 mammalian species with h-index: a case study
Source: Gigascience. 2022 Aug 13;11:giac074. doi: 10.1093/gigascience/giac074 (PMC9375528; doi:10.1093/gigascience/giac074)
Supplement: giac074_Reviewer_2_Report_Original_Submission — Louise McRae -- 1/14/2022 Reviewed [file giac074_reviewer_2_report_original_submission.pdf]

Reviewer Report

Title: Quantifying research interests in 7,521 mammalian species with h-index: a case study

Version: Original Submission **Date:** 1/14/2022

Reviewer name: Louise McRae

Reviewer Comments to Author:

Tam et al tackle an interesting and important topic of taxonomic bias in research and extend the research by including more species than in previous studies and use a different metric for assessing the amount of research. They use bibliometric records to assemble a large data set of mammal publications and assess research interest using the h-index as a metric. They identify 6 potential drivers affecting research interest and used a Bayesian generalised linear mixed model to assess the importance of these factors. They found higher h-index was associated with larger body size, temperate latitudes, lower extinction risk, with higher human uses. Bringing together large data sets in this way is hugely valuable and challenging, but we had a few concerns and questions on the process and conclusions drawn.
General points
Narrative around research interest
We were confused by the focus of the manuscript at times. The results seem to have a broad scope of any research but the introduction and discussion seemed to take a conservation slant to some degree. Given that a lot of the research is on mammals used for biomedical research, pets and other domesticated species (human focussed), the conservation narrative does not seem like it should be as relevant in explaining the patterns observed (species focussed). For example, the prevalence of certain mammal species (e.g. Mus musculus) in the literature arguably has more to do with what research gets funded, what the priorities are in terms of medical research, rather than the interest of the individual researcher themselves. However, the argument about charismatic species and logistics of geography (needing to conduct fieldwork) would seem to only be relevant to wild species and not domesticated ones. Would it be useful to divide the data set up to scrutinise some of the findings further e.g. separate by broad discipline or by removing domesticated species and/or ones which are commonly used in research? You mention in limitations about looking at topics of research if full text was available, so perhaps the first suggestion would be hard, but perhaps journal title could help with that. That would be interesting to see the biases in the remaining species which would give a better picture of biases in conservation and ecological research, if that is the main interest of the authors.
Temporal component
Have the authors considered how time may influence their results? Firstly, species which have historically been well studied have had longer to accrue more papers/citations and therefore a higher h-index so could including publications from that far back introduce some bias? Also there have been changes in disciplines from over time particularly within ecology and conservation which could influence the research focus from good model species for ecology (e.g. predator-prey dynamics) to species which are important from a conservation perspective. Is it useful/possible to put year of publication in the model to address this? You mention in limitations that other similar indices, which have temporal

components, could be used in the analyses, but do not explain why the h-index was chosen in this study, instead of these other similar indices.

Finally, the Red List status is a snapshot and a fairly recent one so how might that affect the conclusions drawn about extinction risk? Could it be more appropriate to look at papers in the last 30 years or so to make conclusions about red list status and conservation generally?

Workflow documentation

We found some aspects missing or hard to follow e.g. number of species in the data set, how the species searches were done and a note on how the workflow could be scalable. Why did the authors use GBIF for the centroids when actual species ranges are available from the Red List, or are these less comprehensive in terms of species coverage?

Specific comments:

Findings

Line 24: The authors may need to be more circumspect with regards to the conclusion about human use. Whilst it is accurately defined as 'use documented' versus 'no use documented', the Red List information recorded is not comprehensive and that particular field on use has been a recommended one so whilst it is a very believable result, it might need to be caveated that those with no use documented are not necessarily not used.

Introduction

Line 49: this is an interesting hypothesis to introduce here but it isn't picked up again in the discussion. It seems like it should be relevant to come back to

Method

Line 94 and Line 105: how did the number of species change from 6,952 to 7,521? This is implied later in the manuscript as being partly to do with synonyms but should be explicit here in the methods. It is a larger number than known mammal species totals so we think that should be introduced here

Line 102: Was Near Threatened excluded or just missed from the list? If it was not included then this needs an explanation

Line 132: in which fields were the binomial names searched?

Line 140: we were surprised that the Red List status was only 69.72% complete when all mammals have been assessed. Is there an explanation for this?

Results

Line 195-6: Given the early focus of these studies is there value in looking at more recent trends in the data set so as to not be skewed by changes in disciplines? Also, how did the authors deduce this - were there fields from the publication search that were used to do this?

Line 188-190: this seemed like a huge result and possibly more important than some others. Incredible that almost a third of species have an h-index of 0, implying that there were no publications about these species that had been cited even once in the time period covered. It is unclear in the method which fields were searched. Could older papers that are scanned and so the main text is not searchable contribute to this? If only title and abstract are searched this should perhaps be mentioned as a limitation as some species could be missed.

Discussion

Line 242-244: Can this be concluded if there was no significant interaction in the model between body size and Red List status?

Line 245-247: This seems slightly contradictory to the previous conclusion - this one suggests that large mammals (a higher proportion of which are threatened with extinction) have received more research interest

Lines 263-267: Is this comparison a fair one given that the disciplines and funding that underpin research on the tree-kangaroo and sheep/cattle are very different?

Figures

Figure 3: use of symbol size seems odd for a categorical variable - could the symbol and colours be swapped for the other variable? It might be clearer

Co-reviewers: Charlotte Benham, Hannah Puleston, Kate Scott-Gatty, Louise McRae

Methods

Are the methods appropriate to the aims of the study, are they well described, and are necessary controls included? Choose an item.

Conclusions

Are the conclusions adequately supported by the data shown? Choose an item.

Reporting Standards

Does the manuscript adhere to the journal's guidelines on minimum standards of reporting? Choose an item.

Choose an item.

Statistics

Are you able to assess all statistics in the manuscript, including the appropriateness of statistical tests used? Choose an item.

Quality of Written English

Please indicate the quality of language in the manuscript: Choose an item.

Declaration of Competing Interests

Please complete a declaration of competing interests, considering the following questions:

- Have you in the past five years received reimbursements, fees, funding, or salary from an organisation that may in any way gain or lose financially from the publication of this manuscript, either now or in the future?
- Do you hold any stocks or shares in an organisation that may in any way gain or lose financially from the publication of this manuscript, either now or in the future?

- Do you hold or are you currently applying for any patents relating to the content of the manuscript?
- Have you received reimbursements, fees, funding, or salary from an organization that holds or has applied for patents relating to the content of the manuscript?
- Do you have any other financial competing interests?
- Do you have any non-financial competing interests in relation to this paper?

If you can answer no to all of the above, write 'I declare that I have no competing interests' below. If your reply is yes to any, please give details below.

We declare that we have no competing interests

I agree to the open peer review policy of the journal. I understand that my name will be included on my report to the authors and, if the manuscript is accepted for publication, my named report including any attachments I upload will be posted on the website along with the authors' responses. I agree for my report to be made available under an Open Access Creative Commons CC-BY license (http://creativecommons.org/licenses/by/4.0/). I understand that any comments which I do not wish to be included in my named report can be included as confidential comments to the editors, which will not be published.

Choose an item.

To further support our reviewers, we have joined with Publons, where you can gain additional credit to further highlight your hard work (see: https://publons.com/journal/530/gigascience). On publication of this paper, your review will be automatically added to Publons, you can then choose whether or not to claim your Publons credit. I understand this statement.

Yes Choose an item.
