# Supplementary material for: Quantifying research interests in 7,521 mammalian species with h-index: a case study
Source: Gigascience. 2022 Aug 13;11:giac074. doi: 10.1093/gigascience/giac074 (PMC9375528; doi:10.1093/gigascience/giac074)
Supplement: giac074_Reviewer_2_Report_Revision_1 — Louise McRae -- 5/18/2022 Reviewed [file giac074_reviewer_2_report_revision_1.pdf]

Reviewer Report

Title: Quantifying research interests in 7,521 mammalian species with h-index: a case study

Version: Revision 1 **Date:** 5/18/2022

Reviewer name: Louise McRae

Reviewer Comments to Author:

Thank you to the authors for addressing the review comments and providing many helpful clarifications. We have a few minor points that might be worth considering as you finalise your manuscript. We have made reference to the reply number in the manuscript comments file.
Reply 14: It might be worth double checking the Red List match-up as it seems unusual that none of the NT species would be included (there are 377 of them). Perhaps there is an explanation for it that we have missed. We did look at the final data set on GitHub and thought there were some NT species e.g. Bison bison which had NA for Red List category but that is the binomial on the Red List. We could be mistaken in how the data sets were matched up And possibly because we're not familiar with the packages used, but just wanted to mention this in case you wanted to check further. Although, appreciate that reconciling taxonomy is no easy task!
Reply 17: Thank you for addressing our comment about the temporal aspect in the data set. For clarity, the authors could mention how many titles were skimmed. Understandably it needs to be a spot check of a sample of the titles. Either that or perhaps reword slightly to be more speculative e.g. "Upon skimming the titles of some articles, we noted that early publications in these species appeared to be mostly comparative anatomy studies."
Reply 19 and 20: We think the rewording here is a better reflection of the results but that the sentence needs clarifying a bit - e.g. the part starting with however sounds like it is meant to contradict the previous sentence. Some suggested wording (assuming we have understood the meaning):
"These findings could jointly indicate that larger mammals that are less endangered could be attracting more research attention than smaller mammals that are severely endangered. Since taxa with larger mammals, such as the big cats and African megafauna, are typically considered more charismatic [8,42], larger mammals may receive more research interest than smaller mammals, regardless of whether or not they are threatened"
Group reviewers: Louise McRae, Charlotte Benham and Hannah Puleston, Institute of Zoology (ZSL)

Methods

Are the methods appropriate to the aims of the study, are they well described, and are necessary controls included? Choose an item.

Conclusions

Are the conclusions adequately supported by the data shown? Choose an item.

Reporting Standards

Does the manuscript adhere to the journal's guidelines on minimum standards of reporting? Choose an item.

Choose an item.

Statistics

Are you able to assess all statistics in the manuscript, including the appropriateness of statistical tests used? Choose an item.

Quality of Written English

Please indicate the quality of language in the manuscript: Choose an item.

Declaration of Competing Interests

Please complete a declaration of competing interests, considering the following questions:

- Have you in the past five years received reimbursements, fees, funding, or salary from an organisation that may in any way gain or lose financially from the publication of this manuscript, either now or in the future?
- Do you hold any stocks or shares in an organisation that may in any way gain or lose financially from the publication of this manuscript, either now or in the future?
- Do you hold or are you currently applying for any patents relating to the content of the manuscript?
- Have you received reimbursements, fees, funding, or salary from an organization that holds or has applied for patents relating to the content of the manuscript?
- Do you have any other financial competing interests?
- Do you have any non-financial competing interests in relation to this paper?

If you can answer no to all of the above, write 'I declare that I have no competing interests' below. If your reply is yes to any, please give details below.

'I declare that I have no competing interests'

I agree to the open peer review policy of the journal. I understand that my name will be included on my report to the authors and, if the manuscript is accepted for publication, my named report including any attachments I upload will be posted on the website along with the authors' responses. I agree for my report to be made available under an Open Access Creative Commons CC-BY license (http://creativecommons.org/licenses/by/4.0/). I understand that any comments which I do not wish to be included in my named report can be included as confidential comments to the editors, which will not be published.

Choose an item.

To further support our reviewers, we have joined with Publons, where you can gain additional credit to further highlight your hard work (see: https://publons.com/journal/530/gigascience). On publication of this paper, your review will be automatically added to Publons, you can then choose whether or not to claim your Publons credit. I understand this statement.

Yes Choose an item.
